# A systematic review of research investigating the physiological and psychological effects of combining *Ginkgo biloba* and *Panax ginseng into a single treatment* in humans: Implications for research design and analysis

**DOI:** 10.1002/brb3.1217

**Published:** 2019-02-06

**Authors:** Jonathon Lee Reay, Paul van Schaik, Christopher James Wilson

**Affiliations:** ^1^ Department of Psychology, School of Social Sciences, Humanities and Law Teesside University Middlesbrough UK

**Keywords:** cognitive function, Gincosan, *Ginkgo biloba*, memory, *Panax ginseng*

## Abstract

**Background and Purpose:**

The traditional herbal supplements *Panax ginseng *and *Ginkgo biloba* are self‐medicated by members of the general public and prescribed by healthcare professionals in some EU countries for numerous health complaints. Clinical evidence is mixed and mechanisms of action are not fully understood. There is clinical interest into the synergistic effects of combining both herbs.

**Methods:**

We systematically review the literature investigating the effects of combination treatments on physiological and psychological outcomes in humans. We identified all studies meeting inclusion criteria: (a) written in English; (b) peer‐reviewed; (c) conducted in humans; (d) including either a proprietary *Panax ginseng*/*Ginkgo biloba* treatment or a study preparation containing both; (e) placebo‐controlled; (f) utilizing standardized extracts. We critically discuss each trial; calculate standardized effect sizes where possible and provide recommendations for research design and analysis.

**Results:**

Eight studies were identified and all investigated a proprietary combination treatment, Gincosan^®^. Studies are of high quality and robust; however, practice effects, choice of statistical model, and reliance upon null‐hypothesis significance testing hinder generalized estimates of effect. The most consistent results are benefits to aspects of the circulatory/cardiovascular system in patient populations and “secondary memory” performance in patient and healthy populations. Two studies demonstrate synergy in healthy populations following a single dose; however, synergy in patient populations and following repeated dosing has not yet been directly tested.

**Conclusions:**

A *Panax ginseng* and *Ginkgo biloba* combination treatment can improve aspects of physiological and cognitive function in humans; however, evidence for synergy requires further investigation and future research should directly investigate synergy following repeated dosing.

## 1 INTRODUCTION

The traditional herbal supplements *known as Panax ginseng *and *Ginkgo biloba* have been ingested by humans for millennia for their purported health benefits (Lee, Chu, Sim, Heo, & Kim, 2008) and in the 21st century it is now common for members of the general public to use herbal supplements in their treatment programs for physiological and psychological disorders (Benzie & Wachtel‐Galor, 2011). Indeed, both herbs often feature in the list of most commonly purchased over‐the‐counter (OTC) extracts and in some western countries (e.g., Germany, Sweden) are also prescribed by healthcare professionals for the treatment of numerous medical conditions (e.g., asthenia, dementia, diabetes, tinnitus, and vertigo) (Isah, 2015; Patel & Rauf, 2017).

Despite this popularity, the evidence to support the clinical efficacy of both *Panax ginseng *and *Ginkgo biloba* is limited and the results and conclusions drawn from the available research studies are mixed and are undoubtedly linked to a number of factors related to study design and analysis. For example, individual studies have assessed different extracts, administered different treatment doses for different periods of time, studied different populations of interest, and measured different outcomes making generalized estimates of effect more difficult. Indeed, it is clear that very few herbal supplements have been exposed to systematic investigation but rather individual studies have been conducted.

We would argue that the best evidence comes from those studies that have implemented randomized control methods and studied high‐quality standardized extract.^1^ Such evidence has demonstrated that standardized extracts of both *Ginkgo biloba* and *Panax ginseng* can benefit cognitive function in healthy and patient populations (e.g., Gauthier & Schlaefke, 2014; Lee et al., 2008; Scaglione, Pannacci, & Petrini, 2005; Yakoot, Salem, & Helmy, 2013). It is worth noting that the biological mechanisms of action are still poorly understood;^2^ however, both in vivo* and *in vitro studies have identified biological effects of the individual active chemicals when studied in isolation that may underpin behavioral change; however, much less is known about how the individual active chemicals impact the biological system concomitantly (Lü, Yao, & Chen, 2009; Smith et al., 2014). For example, Smith, Williamson, Putnam, Farrimond, and Whalley (2014) and Nah (2014) have shown that the active constituents of *Panax Ginseng* (triterpenoid glycosides) have numerous effects upon the structural integrity and neurotransmitter pathways of the central nervous system (CNS) and Rokot et al. (2016); Rudakewich, Ba, and Benishin (2001) and Li et al. (2016) have shown prevention of ß‐amyloid aggregation shown to be important for neurodegenerative disease. Similarly, Cho (2012) & Smith and Luo (2004) have shown numerous effects of the active compounds of *Ginkgo biloba* (ginkgolides, bilobalides, and flavonoids) upon the structural integrity and neurotransmitter pathways of the CNS and to reliably modulate blood flow in both the peripheral nervous system (PNS) and CNS. In addition, Kehr et al. (2012); Ribeiro et al. (2016) and Mashayekh et al. (2011) have demonstrated the modulation of biological pathways related to a number of psychological disorders.

Taken together, this evidence suggests a clinical benefit for both *Ginkgo biloba* and *Panax ginseng* when consumed in isolation. However, over the last 20 years there has also been clinical interest and enquiry into the synergistic effect of combining *Ginkgo biloba* and *Panax ginseng* into a single “treatment.” A significant challenge for such research will be to establish an understanding of the specific quantities of each extract^3^ needed to produce synergy, if indeed, synergy can be accomplished.

The aim of the current review were to systematically summarize and critically discuss the findings from research investigating the physiological and psychological effects of combining *Ginkgo biloba* and *Panax ginseng* into a single treatment, in humans.

## 2 METHODS

Here, we describe the criteria we employed to select studies for inclusion in this systematic review. A data search was conducted using the search terms “*Panax ginseng*” and “*Ginkgo biloba*” coupled with “mood,” “cognitive function,” “mental performance,” “memory,” and “attention.” Abstracts were read and manuscripts were selected for further reading^4^ if they met the following criteria: (a) written in English; (b) peer‐reviewed; (c) conducted in human participants; (d) included, either, a proprietary *Panax ginseng*/*Ginkgo biloba* treatment or a study preparation containing both *Panax ginseng* and *Ginkgo biloba*; (e) included a placebo control arm; (f) used high‐quality standardized extracts. Eight manuscripts satisfied all six of the inclusion criteria and are included in this review. The eight studies all used a commercially available standardized product—Gincosan^®^.

### 2.1 Our approach to reporting

In this review, we provide a summary and critical discussion of each trial (section 3) and a tabularized summary of all studies (Appendix S1) and a tabularized summary of the clinical effects reported for predefined primary outcomes (Table S1). We provide additional data (effect sizes—Cohen's *d*; Cohen's *d*_z_) for those studies that have provided enough detail^5^ in their manuscript to allow this calculation (section 3.3, Appendices S2 and S3) and we provide discussion/evaluation and recommendations for research design and analysis (section 4).

## 3 RESULTS

### 3.1 Description of the studies

Each of the reviewed studies assessed the efficacy of a standardized product—Gincosan^®^. The product was registered in Switzerland in 1989 and contains a standardized *Panax ginseng* extract G115^6^ and standardized *Ginkgo biloba* extract GK501.^7^ Studies have tested effects on the same day as treatment ingestion (referred to as an acute effect), on the day(s) following treatment cessation (referred to as a chronic effect) and/or on the same day as treatment ingestion but following repeated dosing (referred to a superimposed effects). The earliest study summarized in this review was published in 1992 and the most recent was published in 2004. The study designs are robust and authors have used a range of statistical techniques to explore their research questions; however, none report effect sizes to allow exploration of their data and all rely upon null‐hypothesis significance testing. A range of treatment doses and outcome measures have been used between trials, with some focusing upon psychological outcomes (majority of them being cognitive outcomes) and some focusing upon physiological outcomes (all related to the circulatory/cardiovascular system). Two of the studies compared their combination treatment directly with its constituent parts in isolation (*Panax ginseng* and *Ginkgo biloba) to allow direct discussion of synergy.* Five of the studies were conducted by the same research group. All studies are described in Appendix S1 and the effects on the predefined primary endpoints are summarized in Table 1.

**Table 1:** Summary of results reported for those outcome measures and endpoints identified as of primary interest by authors, following a single dose (SD) and repeated dose (RD)

	80 mg		160 mg		320 mg		640 mg		960 mg	
	SD	RD	SD	RD	SD	RD	SD	RD	SD	RD
Blood pressure	—	—	↑p	—	↑p	—	—	—	—	—
Heart rate	—	↑pb.i.d	—	—	↑p	—	—	—	—	—
Spontaneous platelet aggregation	—	—	↑p	—	↑p	—	—	—	—	—
Cutaneous erythrocyte velocity in capillaries	—	—	—	—	↑p	—	—	—	—	—
Cerebral blood flow	—	—	—	↑pb.i.d	—	—	—	—	—	—
Concentration	—	—	—	↑pb.i.d	—	—	—	—	—	—
Visual scanning	—	—	—	↑pb.i.d	—	—	—	—	—	—
Quality‐of‐memory Index (accuracy)	—	—	—	—	—	↑pb.i.d/↑h	—	—	↑h	—
Quality‐of‐memory Index (speed)	—	—	—	↑pb.i.d	—	—	—	—	—	—
Secondary memory sub‐factor	—	—	—	—	—	—	—	—	↑h	—
Speed of attention	—	—	—	—	↓h	—	↓h	—	—	—
Mental arithmetic	—	—	—	—	↑h	—	↑h	—	↑h	—

Note

Upward arrow indicated benefit for treatment over placebo whereas a downward arrow indicates a decrement for treatment. Sample population is indicted by “p” (patient) and “h” (healthy). Dose was taken in a single ingestion unless stated (b.i.d—twice per day). As an example, 80 mg, consumed twice per day (daily dose equates to 160 mg) of repeated ingestion improved heart rate, in a patient population, relative to placebo.

### 3.2 Study results by trial

We present the published manuscripts in chronological order for ease of reading.

#### 3.2.1 *Acute effect of Gincosan^®^*versus* placebo in a clinical sample (*Kiesewetter, Jung, Mrowietz, & Wenzel, 1992 *)*

The earliest report highlighting the potential clinical efficacy of Gincosan^®^ comes from Kiesewetter et al. (1992) who detail the results of two small trials. The first trial does not meet a satisfactory level of methodological robustness as it fails to incorporate a placebo control and for this reason is not included further in this review. The second trial used a double‐blind, placebo‐controlled cross‐over design to investigate, in 10 volunteers suffering rheological abnormalities, the physiological effects of treatment 60 min after ingesting a single dose of 160 and 320 mg of Gincosan^®^. Results confirmed the safety and tolerability of treatment and demonstrated improvements in blood pressure, heart rate, spontaneous platelet aggregation, and cutaneous erythrocyte velocity in capillaries. The larger dose (320 mg) demonstrated the stronger pattern of effect. These results were encouraging and gave researchers their first clinical evidence of the potential for combining *Panax ginseng* and *Ginkgo Biloba*. As Kiesewetter et al. (1992) focused purely upon physiological effects and did not assess any behavioral outcomes, there was a clear need to investigate the potential for Gincosan^®^ to modulate human behavioral/cognitive process.

#### 3.2.2 *Chronic or superimposed chronic/acute effect of Gincosan^®^*versus* placebo in a clinical sample (*Kwiecinski, Lusakowska, & Mieszkowski, 1997 *)*

The first study to investigate the clinical effects of Gincosan^®^ for human behavior was reported by Kwiecinski et al. (1997) who used a double‐blind, randomized, placebo‐controlled, between‐subjects design. Eighty‐five volunteers (age range 43–72 years) all presenting with at least one symptom of cerebrovascular disorder enrolled in a 12‐week trial consisting of a 4‐week placebo run‐in phase and an 8‐week treatment phase. During the latter phase, participants ingested 160 mg b.i.d. and completed clinical assessments at 4 and 8 weeks. However, it is not clear from the paper if testing on week 4 and week 8 was completed in the absence or presence of that day's treatment dose; therefore, the results could relate to “pure” chronic effects^8^ or superimposed chronic/acute effects.^9^

Despite this uncertainty, the results demonstrated for the first time that Gincosan^®^ can modify behavior in a patient population, specifically showing improved concentration and forgetfulness at the 8‐week assessment point.^10^ In addition, results also report improved cognitive processing at the same assessment point specifically reporting improved (faster) visual scanning ability. However, this latter result should be viewed with some caution, as it ought to be noted that, firstly, the effect was evident in only the more difficult version of the visual scanning task and, secondly, the statistical test underpinning the effect was a within‐group comparison rather than a between‐group comparison. In fact, if one considers the “actual” processing speed of each group (Table S3 in Kwiecinski et al., 1997) it is clear that the placebo group outperformed the treatment group at baseline and at the 8‐week assessment point;^11^ therefore, highlighting the need for a between‐group comparison whilst controlling for baseline performance. Despite the above cautionary concern, it is now commonplace, some two decades later, to find frequent reports of complex interactions between task, task demand, and treatment efficacy. With this in mind, Kwiecinski et al. (1997) may have provided the first tentative evidence of the interplay between task demand and the behavioral efficacy of Gincosan^®^. In addition to these behavioral effects, Kwiecinski et al. (1997) also report increased mean blood flow velocity in the middle cerebral artery. This effect has clear clinical relevance to Kwiecinski et al. (1997)’s specific study population as they all suffer cerebrovascular problems; however, it also provides the first tentative evidence that the well‐documented ability of *Ginkgo biloba* 12 to improve the vasoregulating activities of arteries, capillaries, and veins when consumed in isolation is maintained when consumed in conjunction with *Panax ginseng*;^13^ however, further research is clearly needed to allow any firm conclusions to be made with regard to the effects of Gincosan^®^ on blood flow. One limitation of the study is that it did not systematically investigate cognitive function using a standardized testing platform(s); therefore, at this point in time it was difficult to fully comment upon the effects of Gincosan^®^ on human behavior. In addition, it is not clear if the week of testing was consistent across participants and therefore how many days of treatment each participant completed. Our assumption may be that each participant completed assessments on the last day of the fourth and eighth week of treatment; therefore, the fourth week testing point corresponds to day 28 and the eighth week testing point corresponds to day 56. In addition, the study did not explicitly test the effects following a single dose,^14^ nor was there any attempt to investigate dose response effects, as Kwiecinski et al. (1997) used a design with only one treatment arm.

#### 3.2.3 *Superimposed chronic/acute dose‐response effect doses of Gincosan^®^*versus* placebo in clinical sample (*Wesnes, Faleni, & Hefting, 1997 *)*

Published in the same year as Kwiecinski et al. (1997), a third study conducted by Wesnes et al. (1997) goes some way to address some of the limitations of Kwiecinski et al. (1997). Wesnes et al. (1997) implemented a double‐blind, placebo‐controlled, between‐subjects design and randomly allocated sixty‐four older adults (mean age 54 years) suffering of neurasthenic complaints to receive one of three treatment dosing regimens for 90 days (80 mg b.i.d., 160 mg b.i.d. or 320 mg b.i.d.). Wesnes et al. (1997) employed a gold‐standard computerized assessment battery (Cognitive Drug Research) to assess two fundamental cognitive constructs (memory and attention) and some elements of subjective mood. In addition, information‐processing speed (Vienna Determination Test) and heart rate during maximum exercise were assessed.^15^ Clinical efficacy was measured after an acute dose (day 1) and at two further time points following repeated dosing (day 30 and day 90). On all three assessment days, clinical efficacy was assessed 1 hr after a morning dose and again 1 hr after an afternoon dose. Although the experimental design allows consideration of the effects following a single dose (e.g., effects on day 1) and following repeated dosing (effects on day 30 and day 90), the design does not allow for consideration of “pure” chronic effects (i.e., the effects on day 30 and day 90 before that day's treatment). In addition, despite the complexity and robustness of the experimental design, Wesnes et al. (1997) stipulated one primary time point of interest and three specific outcomes as primary focus. The former was 1 hr after the morning dose on day 90 and the latter were (a) a composite memory score labeled “quality‐of‐memory index”^16^ derived from the Cognitive Drug Research battery, (b) performance on the Vienna Determination Test, and (c) heart rate during maximum exercise load. Starting with the primary time point of interest, results revealed a clear dose‐dependent and domain‐specific effect. The middle (160 mg) and larger (320 mg) dose led to benefits to memory performance;^17^ however, there was no effect of the lowest dose (80 mg). In contrast, the lowest dose (80 mg) revealed benefits to participants’ physiological response to exercise in the guise of lower heart rate (HR) at maximum effort, whereas the middle (160 mg) and larger dose (320 mg) had no effect on HR. Results reveal no effect of any dose on the Vienna Determination Test.^18^

Although it is essential to assess the efficacy of treatments at the primary time points of interest defined by the authors (as such time points are chosen based upon the best evidence to date), it is nevertheless important to consider any effect reported at earlier and later time points (referred to as secondary time points of interest). This will allow for consideration of any therapeutic “window” to be considered (e.g., when does an effect start? How long does it last?) and any adverse effects that may occur before any therapeutic effects become apparent and after treatment is stopped. Aside from the primary time point stipulated (1 hr after treatment ingestion on day 90), the current study revealed a number of effects at secondary time points worthy of consideration and discussion, particularly those effects revealed at the same assessment point (i.e., 1 hr after the morning dose) on day 1 and day 30. The first and arguably the most important effect at these secondary time points of interest, given the profile of effects at day 90, is that all three doses improved accuracy of the quality‐of‐memory index on day 1 and day 30 1 hr after the morning dose. This clearly demonstrates that all three doses of Gincosan^®^ improved memory performance following a single dose and following repeated dosing for 30 days. Consideration of Figure 1 in Wesnes et al. (1997) clearly shows continued improvement of the lower and middle dose on day 90, relative to predosing (thereby ruling out the possibility that habituation/tolerance to treatment has occurred) and clearly demonstrates that the effect is “lost” at day 90, for the lowest and middle dose, because of a “gain” in placebo performance. We would argue that this highlights the need for researchers to keep robust control over practice effects. Although Wesnes et al. (1997) did implement some control for practice effects (training sessions were conducted prior to baseline assessment), the design could have benefitted from a placebo run‐in phase, similar to that used in Kwiecinski et al. (1997), as well as a placebo run‐out phase to assess the longevity of the therapeutic effect. Interestingly, and rather unexpectedly, the study also revealed a biphasic effect of treatment dosing time, as all three doses demonstrated impaired memory performance following the afternoon dose. This biphasic effect was unexpected but has clear implications for clinical application with regard to daily dose and timing of dose. The unexpected biphasic effect was further investigated, 3 years later.

#### 3.2.4 *Chronic, superimposed chronic/acute, and dose‐response effect of various doses of Gincosan^®^*versus* placebo in a nonclinical sample (*Wesnes, Ward, McGinty, & Petrini, 2000 *)*

In a fourth trial, utilizing healthy volunteers, Wesnes et al. (2000) address many of the methodological limitations of Wesnes (1997) and specifically tested the robustness of the unexpected biphasic effect reported in Wesnes et al. (1997). Wesnes et al. (2000) conducted a multi‐center trial utilizing a double‐blind, placebo‐controlled, between‐subjects design and randomly allocated 256 healthy middle‐aged adults to receive 160 mg b.i.d. or 320 o.d. The experimental protocol was exceptionally robust, spanning a 16‐week period (~112 days) requiring all participants to complete a two‐week placebo run‐in phase, a twelve‐week treatment phase, and a further two‐week treatment washout phase. Testing was conducted before and after the placebo run‐in phase (study days 1 and 2, respectively), at four (~28 days of treatment), eight (~56 days of treatment), and twelve weeks (~84 days of treatment) during the treatment phase (study days 3, 4, and 5, respectively) and at 2 weeks after treatment cessation (study day 6). Treatment commenced after study day 2 and ceased after study day 5. On each study day, participants completed assessments 1 hr before dose and 1, 3, and 6 hr after dose, utilizing the CDR battery. Unlike Wesnes et al. (1997), Wesnes et al. (2000) did not specify a primary time point of interest; however, four specific outcome measures of primary focus were identified ([1]quality‐of‐memory index; [2]speed of memory; [3]power of attention; [4]continuity of attention). Despite the elegant and robust methods used in this study, the authors have chosen a rather conservative analytical approach to explore the effects of Gincosan^®^. We would argue that the approach taken does not allow the authors the ability to fully explore the clinical efficacy of Gincosan^®^. Wesnes et al. (2000) relied upon an omnibus four‐way (2 × 2 × 4 × 4) ANOVA to explore their research questions rather than the more “powerful” planned contrasts used in Wesnes (1997) or alternatively a more conservative post hoc analysis plan.

Despite this, the ANOVA revealed a significant main effect of treatment on the primary outcome measure of “quality‐of‐memory index” showing direct replication of domain specificity highlighted in Wesnes et al. (1997). The interpretation of results from the ANOVA suggests that Gincosan^®^ can improve memory performance at all postdose time points (1, 3, and 6 hr after dose) across all testing weeks (4, 8, 12, and 14 weeks) (see Figure 1 in Wesnes et al. (2000)). In addition, the main effect of treatment, coupled with an absence of a main effect of, or interaction with, dosing regimen was taken as confirmation that the biphasic effect reported in Wesnes et al. (1997) was not present in Wesnes et al. (2000). Finally, as there was no interaction with assessment day, the main effect of treatment was taken as an indication that treatment effects were still present 2 weeks after treatment cessation, providing the first evidence of the longevity of Gincosan^®^’s memory‐enhancing effects. Although this pattern of results is clear and the ANOVA confirmed an absence of any significant higher‐order interaction effect (and hence ruling out any necessity to statistically explore the main effect of treatment further) we argue that it would have been informative to explore the main effect further. To highlight this point, the protocol and analysis plan used by Wesnes et al. (2000) provides the first tentative suggestion that there is no dissociation between “pure” chronic effect and superimposed acute/chronic effect, as the protocol included a predose testing session on each testing day and the analysis included predose testing time as a factor in the analysis. As mentioned previously, the analysis did not find any significant interaction with testing point (hence the conclusion that there is no dissociation of effect); however, consideration of Figure 1 in Wesnes et al. (2000) it is perfectly clear that there was no benefit of treatment over placebo at the predose testing session.^19^ We would argue that this implies a dissociation of effect between “pure” chronic and superimposed effects and clearly warrants further investigation. Indeed, although the analysis did not show a significant interaction effect it did report a trend (*p* = 0.08) toward an interaction with time of testing (page 357). However, the authors argue in their discussion that this is “driven” by the pattern of results at the postdose testing point. In contrast, we would argue that it is driven by the lack of effect at this predose testing point and we would argue that this highlights the need to explore the data in greater depth to allow further understanding of the treatment effects. Similarly, we would argue that any direct comparison between placebo and treatment on any discrete testing day (4, 8, 12, or 14) would be unlikely to reveal a significant difference between treatment and placebo at the 3‐hr testing point (see Table S2 in Wesnes, 2000). Both of these issues have obvious impacts upon our understanding of treatment efficacy and practical application, which is lost in the authors’ choice of statistical approach and analysis plan. Despite this, the general results of Wesnes et al. (2000) are consistent with those of Wesnes et al. (1997). Both studies demonstrate Gincosan^®^’s therapeutic efficacy for improved memory performance after only 4 weeks (~30 days) of repeated ingestion and provide evidence to suggest that repeated ingestion does not lead to treatment tolerance at 12 weeks (~90 days). A final point to raise is that as participants were still ingesting placebo on study day 2 (subsequently used for baseline adjustment of postdose assessment points) and therefore Wesnes et al. (2000) was not able to assess the acute effects following a single dose further. However, a further series of trials have subsequently and systematically investigated the effects of acute dosing with Gincosan^®^ on cognitive function, providing further insight into dose and domain specificity. They were the first to compare Gincosan^®^ directly with its constituent parts (*Ginkgo biloba* and *Panax ginseng*) in the same trial or using the same population, protocol, and analysis plan across trials. These acute trials will now be discussed.

#### 3.2.5 *Acute effect of Gincosan^®^*versus* placebo in a nonclinical sample (*Kennedy, Scholey, & Wesnes, 2001 *)*

The first of a series of acute studies was reported in 2001 by Kennedy et al. who implemented a single‐center trial and used a placebo‐controlled, double‐blind, balanced, cross‐over design. Twenty healthy young adults (mean age 20.6 years) attended three study days, each separated by a seven‐day washout period, and were randomly allocated to receive 320, 640, and 960 mg in a specific order defined by Latin square. Treatment was ingested in the morning following an overnight fast and testing was completed before treatment (baseline) and 1, 2.5, 4, and 6 hr after treatment. The CDR battery was used and the authors stipulated six primary outcomes of focus ([a] quality of memory; [b] secondary‐memory sub‐factor, [c] working‐memory sub‐factor; [d] speed of memory; [e] speed of attention; [f] accuracy of attention);^20^ however, the authors did not stipulate specific time points of primary interest. For the primary outcomes of focus, results revealed a clear dose and domain specificity of effect. The larger dose (960 mg) improved “quality of memory” at the 1‐ and 6‐hr postdose testing point, demonstrating for a third time Gincosan^®^’s cognition enhancing effect is specific to memory‐processing and not attentional‐processing. Consideration of Figure 2 in Kennedy et al. (2001) suggests the result could more accurately be described as an amelioration/protection against the natural decline in performance throughout the day seen in the placebo group, whereas in Wesnes et al.’s (1997) and Wesnes et al.’s (2000) results suggest enhanced performance beyond predose levels.^21^ Perhaps surprisingly in Wesnes et al. (1997) all three of the doses tested (80, 160, and 320 mg) revealed improved “quality‐of‐memory” performance 1 hr postdose on day 1 whereas only the largest dose (which Wesnes et al. did not investigate) revealed the treatment effect at the same time point in Kennedy et al. (2001). Therefore, Kennedy et al. (2001) failed to directly replicate the positive effects of the 320 mg dose demonstrated in Wesnes et al. (1997) at 1 hr post‐dose testing point on day 1. This may imply that lower doses show efficacy at this time point in patient populations only and a larger dose is needed for healthy participants to detect benefits. With regard to domain specificity, Kennedy et al. (2001) provide further insight. As discussed earlier, the “quality‐of‐memory” index is a composite score derived from the CDR battery and is a result of performance on a number of individual tasks. Kennedy et al. further sub‐categorized the tasks to form two additional composite outcomes of focus ([a] secondary‐memory sub‐factor and [b] working memory sub‐factor). Interrogation of the sub‐factors, allows Kennedy et al. (2001) to conclude that the “quality‐of‐memory” effect in their study is “driven” by performance of the secondary‐memory sub‐factor and not the working memory sub‐factor. Again, this clearly warrants further investigation. In addition to the memory‐enhancing effects, Kennedy et al. also report an unexpected decrement in performance on attentional tasks as evidenced by the speed of “speed of attention” being significantly slowed by 320 mg dose at the 4‐ and 6‐hr postdose testing point and following the 640 mg dose at the 4‐hr postdose testing point.

#### 3.2.6 *Acute effect of Gincosan^®^*versus* ginkgo, ginseng, and placebo in a nonclinical sample (*Kennedy, Scholey, & Wesnes, 2002 *)*

In the second of the series, Kennedy et al. (2002) conducted another single‐center trial utilizing a placebo‐controlled, double‐blind, balanced, cross‐over design. However, commendably, in this trial Kennedy et al. compared the Gincosan^®^ arm to its constituent parts^22^ in the same study to allow for the first time consideration of any synergistic effects to be directly analyzed. Twenty young healthy participants (mean age 21.2 years) attended three study days, each separated by a seven‐day washout period and were randomly allocated to receive 360 mg *Ginkgo biloba* GK501^®^, 400 mg *Panax ginseng* G115^®^, 960 mg combination Gincosan^®^, and placebo in a specific order defined by Latin square. Treatment was ingested in the morning following an overnight fast and testing was completed before treatment (baseline) and 1, 2.5, 4, and 6 hr after treatment. The CDR battery was used and the authors stipulate the same six primary outcomes of focus as Kennedy et al. (2001) and again did not stipulate specific time points of primary interest. In addition, and to further explore the apparent domain specificity of Gincosan^®^ Kennedy et al. added a further outcome measure (mental arithmetic) to their study to explore the impact of Gincosan^®^ upon more complex cognitive processing (i.e., tasks that draw upon both memory and attentional resources for successful completion, rather than one).

For the six primary outcomes of focus, results revealed once again a clear treatment and domain specificity effect. Kennedy et al. (2002) showed that 960 mg Gincosan^®^ improved “quality of memory” performance in healthy young adults following an acute dose. Additionally, they also confirm the effect is “driven” by performance of the secondary‐memory sub‐factor.^23^ In addition to the replication of enhanced memory performance Kennedy et al. (2002) also partially replicated the decrements (slowing) in “speed of attention” at the 4‐hr postdose testing point, initially reported in Kennedy et al. (2001). However, it should be noted that Kennedy et al. (2001) reported the slowing of performance following the lower dose (320 mg) and not the higher dose (960 mg), while Kennedy et al. (2002) reports the slowing of performance following the higher dose (960 mg). Clearly, more research is needed to further understand the effects of Gincosan^®^ upon attentional processes.

Moving back to the effects on “quality of memory,” further comparisons between Kennedy et al. (2001) and (2002) reveal some similarities and disparities in the treatment‐related effect on “quality of memory” at specific postdose time points. Firstly, and starting with the most consistent effect, both studies clearly demonstrate that Gincosan^®^ can improve “quality of memory” 1 hr after dose in healthy volunteers (Kennedy et al., 2001, 2002).^24^ However, through further scrutiny of the postdose time effects it becomes clear that Kennedy et al. (2001) and (2002) report postdose time effects that are isolated to each study. The most parsimonious explanation for this, despite the similarity and rigor of the methods used and the population tested is that slight variations in study protocol may account for the isolated effects.^25^

We would like to consider one potential variation in some detail—practice effects—and argue that robust experimental design can “cope” with practice effects when each study is considered in isolation. However, if a standardized approach is not implemented between trials, interpretation becomes challenging and this variation may explain disparities between studies. For example, although both studies used a practice day, neither study provide evidence that their participants had reached their individual optimal level of cognitive performance prior to baseline assessments and subsequent intervention. Consequently, neither study provided any reassurance to the reader that simply being more familiar with a task will not lead to further improvements in the performance of that task (and hence any treatment effect may include an element of practice). As stated above, this is not an issue for robust experimental designs as used by Kennedy et al. (2001) and (2002) when considered in isolation and obviously the assumption being made here is that a “stable” base level of performance can be achieved.^26^ However, in Kennedy et al. (2001) and (2002) (as well as Wesnes et al., 1997; Wesnes et al., 2000), participants are completing memory tasks and we argue that performance of such tasks will have a stable base level, at which point no further improvement will be seen in future task completion without an effective intervention. This base level will be achieved when participants habituate to the novel lab environment, understand the specific demands of the task, and stabilize any strategy (e.g., chunking and visualization) used to complete a task. To illustrate this point, we can consider the predose performance levels across Kennedy et al. (2001) and (2002). The assumption we are making here is that two groups, randomly sampled from the same population (i.e., young healthy adults) should not differ in their base performance of a memory task once their stable level has been achieved (i.e., the memory performance of 20 young adults should not differ from the memory performance of a different set of 20 young adults drawn from the same population). However, if one considers the base level performance of Kennedy et al. (2001) and (2002) one can clearly see that performance is different. As an example, “quality of memory” in the placebo condition is reported at 422.79 in Kennedy et al. (2001) compared to a placebo condition of 384.15 in Kennedy et al. (2002) and performance subsequently falls by 13.39% and 41.53%, respectively at 1 hr after dose. As stated above, when we consider the trials in isolation, the robust design used by Kennedy et al. will accommodate for this; however, when we start to compare across trials, it makes interpretation (more) difficult. For example, Kennedy et al. (2002) report enhanced memory performance at all postdose time points except the final time point (6 hr), whereas Kennedy at al. (2001) report enhanced memory effects at the first (1 hr) and last time point (6 hr) only. In addition, the general pattern in Kennedy et al. (2001) is one of an amelioration/protection against a natural fall in performance throughout the day. However, the general pattern in Kennedy et al. (2002) suggests an “actual” improvement from base performance level at the 1 hr and 2.5 hr after dose, rather than an amelioration/protection against a natural decline in performance. This pattern of results could suggest that the results of Kennedy et al. (2002) are being influenced to a greater extent by practice effects because participants had not yet reached their base level of performance during the practice day. Obviously, this does not detract from the treatment effect (both studies showing positive effects of treatment relative to placebo) but it may explain treatment‐related time point disparities between two studies that have implemented the same protocol. It may also explain the lack of effect at the 6‐hr testing point in Kennedy et al. (2002) as one can see that the effect may have been “lost” due to a “gain” in placebo groups performance. Wesnes et al. (1997) and Wesnes et al. (2000) also show “actual” improvements above base levels in the same memory index (i.e., demonstrating practice effects during the treatment phase) in both the placebo and treatment group. Again, this does not lessen the clear treatment‐related benefit, but does raise the issue of controlling for practice effects across studies to allow a clearer “picture” of treatment‐related effects to emerge.

In addition to memory enhancing effects, Kennedy et al. (2002) was also the first to report Gincosan^®^’s effects on a mental arithmetic task. Two versions of the task were administered ([1] serial‐three subtraction task; [2] serial‐seven subtraction task). Results revealed a single time point improvement for the serial‐three task (6 hr) and improvements at two postdose testing points for the serial‐seven task (4 and 6 hr after dose). As the effect is more pronounced on the serial‐seven task, it may be appropriate to tentatively suggest a differential effect of treatment on tasks that require a greater level of mental effort. This is consistent with Kwiecinski (1997) who reported Gincosan^®^ effects were only apparent on the more difficult version of a letter cancellation task used in their study.^27^ With regard to subjective mood, Kennedy et al. (2002) is the first to demonstrate positive effects of treatment on one dimension of mood (content) at 2.5, 4, and 6 hr.^28^

As Kennedy et al. (2002) was the first to compare Gincosan^®^ efficacy to that of its constituent parts (*Ginkgo biloba* and *Panax ginseng*) in a single trial, the results allow direct discussion of the synergistic effect. With regard to the effect on “quality of memory,” results show that both *Ginkgo biloba *and *Panax ginseng* can also improve quality of memory following a single dose; however, the effect was restricted to one postdose time point for *Ginkgo biloba* (6‐hr time point) and *Panax ginseng* (4‐hr time point). This pattern of results provides the first direct evidence to support the notion that a combination of *Ginkgo biloba* (GK501) and *Panax ginseng* (G115) leads to a more powerful/sustained improvement in quality of memory performance across a day and also suggests that the effects start more quickly (i.e., 60 min after ingestion) providing the clearest evidence to date of a synergistic effect.

#### 3.2.7 *Acute dose‐response effect of Gincosan^®^*versus* ginkgo, ginseng, and placebo in nonclinical samples (Scholey et al., 2002)*

In the third and final study of this series of acute trials, Scholey et al. (2002) reports the results of three studies conducted independently of each other in the same lab and provide further insight into the dose response and potential synergistic effects on the completion of a mental‐arithmetic task, first highlighted in Kennedy et al. (2002). All three studies implemented a placebo‐controlled, double‐blind, balanced, cross‐over design and tested efficacy at 1, 2.5, 4, and 6 hr after dose. Study 1 investigated the effects of *Ginkgo bilobo* (GK501) (120, 240, and 360 mg), Study 2 investigated the effects of *Panax ginseng* (G115) (200, 400, and 600 mg) and Study 3 investigated the effects of Gincosan^®^ (320, 640, and 960 mg). In each study, treatment order was determined by Latin square and treatment dose was ingested in the morning following an overnight fast and separated by a seven‐day washout period. Results show for the second time that Gincosan^®^ can improve performance of a mental arithmetic task and clearly demonstrate that the effect of Gincosan^®^ cannot be predicted from its constituent parts, providing further evidence of synergy. However, the evidence for a differential effect of treatment on tasks that require a greater level of mental effort is somewhat mixed. To substantiate these conclusions, we can see that the results of the Gincosan^®^ trial demonstrate a clear dose‐dependent effect on performance of the serial‐three task (easier task). The lower and middle dose improved performance at one time point (4 and 2.5 hr, respectively) whereas the higher dose improved performance at all postdose time points (1, 2.5, 4, and 6). In comparison to the Gincosan^®^ trial, the results of *Ginkgo biloba* (GK501) trial and *Panax ginseng* (G115) trial demonstrated no benefit of any dose of *Panax ginseng* (G115) and a single time point improvement for all three doses of *Ginkgo biloba* (GK501). This pattern of results would suggest a synergistic effect of Gincosan^®^ with the larger dose showing the strongest pattern of effects across the day for this task. In contrast to the dose‐dependent effects revealed for the serial‐three task, results for the serial‐seven task (more difficult task) are in the reverse dose‐dependent direction. For this task, the lower and middle dose of Gincosan^®^ demonstrated the strongest effects across the day with improved performance at 1, 2.5, 4, and 6 and 2.5, 4, and 6, respectively. However, the higher dose demonstrated a weaker pattern of results across the day with the effects limited to two postdose assessment points (2.5 and 6). Nevertheless, again, consideration of the *Ginkgo biloba* (GK501) and *Panax ginseng* (G115) trial provides further support for a synergistic effect as it is clear that the effects were weaker following *Ginkgo biloba* (GK501) and *Panax ginseng* (G115) and in one case the latter treatment led to decrements in performance across the day. These results replicate the findings of Kennedy et al. (2002) who demonstrated the beneficial effect of 960 mg of Gincosan^®^. However, Scholey et al. (2002) failed to replicate the differential effect of the higher treatment dose on tasks that require a greater level of mental effort; although, Scholey et al. (2002) provide evidence that a lower/middle dose may be more beneficial in a healthy population. Finally, if the lower dose is considered in isolation, the results reveal a pattern that once again suggests a differential effect of treatment on tasks that require differing levels of mental effort. Further research is clearly warranted into the relationship between task complexity, treatment dose, and study population.

#### 3.2.8 *Chronic effect of Gincosan^®^*versus* placebo in a female sample (*Hartley, Elsabagh, & File, 2004 *)*

In the final study that has investigated the effect of Gincosan^®^ Hartley et al. (2004) implemented a placebo‐controlled between‐subjects design to investigate Gincosan^®^’s effects upon human memory performance. Seventy (13 withdrew) healthy older (age range 51–66 year) women defined as post‐menopausal were randomly allocated to ingest 320 mg Gincosan^®^ or placebo daily for 12 weeks. Efficacy was measured at baseline and at 6 and 12 weeks. Hartley et al. (2004) used a number of assessment tools taken from a number of standardized tests (e.g., Weschler, 1987; CANTAB CeNes Ltd) and developed a number of in‐house tasks. Results revealed no effect of treatment. Many of our previous discussion points also apply to Hartley (2004). For example, the rationale to investigate a treatment effect in post‐menopausal women could be clearer. At that time, there were no available data to support treatment efficacy in the target sample population and it was known that there are discrete stages to the cycle.^29^ This make it more difficult for Hartley (2004) to establish a treatment effect as sample size issues will have impacted upon the power of the inferential statistical model used to test any interaction with menopausal stage.^30^

An additional point to discuss is the assessment tools used and protocol implemented. With regard to the former, previous research (Wesnes 1997; Wesnes, 2000; Kennedy et al., 2001, 2002) have demonstrated dose‐dependent and domain specificity of effect (i.e., enhanced “Secondary Memory”) and it is not clear why Hartley et al. (2004) did not assess secondary memory in a same way utilizing the same tasks in a new sample population. In addition, our previous discussion regarding practice effects and control over testing point is relevant too. For example, Hartley et al. (2004) implemented their training on the same day immediately prior to their baseline assessment; therefore, practice effects will be evident at baseline and treatment phase. Finally, Hartley et al. (2004) state that efficacy was measured between 2–4 hr after ingestion; therefore, we assume there was a lack of control over the postdose time of testing between participants.

### 3.3 Effect sizes calculated from the studies

The reviewed studies have reported the results of null‐hypothesis statistical significance tests and to a lesser extent confidence intervals to evaluate the magnitude and significance (statistical probability as well as then inferred clinical significance) of treatment effects. To enable further exploration of the magnitude of treatment‐related effects, we calculate and report effects sizes of the reviewed studies (see Appendices S2 and S3) where this is possible based on the reported results presented in the publications. In particular, we report standardized effect sizes that can be interpreted within statistical frameworks for Hartley et al. (2004) and Kennedy et al. (2001 and 2002) (e.g., Cohen, 1988). It was not possible to calculate effect sizes for any other publication.

#### 3.3.1 Effect Size Calculation

The method implemented to calculate effect size varied between papers depending upon the information available. For Hartley et al. (2004), standard deviation values for the data were calculated using the reported standard error of the mean (*SEM*) and reported *N* values. When calculating Cohen's *d* related to the effect of dose (Gincosan^®^ v Placebo) at different time points (Weeks 0, 6, or 12), standard deviation of the baseline score was used as the denominator (see Appendix S1: Tables S1–S4). When calculating Cohen's *d* related to the effect of time (e.g., Week 6 vs. Week 0) within each dose (Gincosan^®^ or Placebo) the pooled standard deviation was used as the denominator (see Appendix S2: Tables S5–S16). Kennedy et al.,(2001) and (2002), only report the “actual” score at baseline with all subsequent time points reported as change from baseline. For this reason, we first calculated an “actual” score for each of the time points using the reported baseline and change from baseline values. Standard deviation values for the data were calculated using the reported standard error and *N* values. The same calculation principles used for Hartley et al. (2004) were then used to calculate the Cohen's *d* related to the effect of dose (see Appendix S2: Kennedy et al., 2001—Tables S17–S22; Kennedy et al., 2002—Tables S33–S38) and related to the effect of time (see Appendix S2: Kennedy et al., 2001—Tables S23–S32; Kennedy et al., 2002—Tables S39–S48). In addition to calculating Cohen's *d* for Kennedy et al. (2001) and (2002), we also calculated Cohen's *d*_z _related to the effect of dose only. This effect size will allow direct comparison with the change from baseline analysis used and reported by the authors (see Appendix S3: Kennedy et al., 2001—Tables S1–S3; Kennedy et al., 2002—Tables S4–S6).

#### 3.3.2 Effect size summary

Calculation of effect size has further demonstrated considerable variability at baseline, giving further weight to our suggestion that baseline scores have not been “stabilized” prior to treatment ingestion. As a result of the variability, interpretation of any postdose effect size using Cohen's *d* becomes more difficult. To avoid over interpretation and to account for large variations at baseline, we applied the following arbitrary rule to explore the effects using Cohen's *d* (between treatment conditions at different time points) baseline Cohen's *d* < 0.1 and postdose Cohen's *d* > 0.2. For Hartley et al. (2004), the effect size calculations revealed a promising result for the Stockings of Cambridge (SoC)^31^ not observed through null‐hypothesis testing. Appendix S2, Table S4 highlights a potential benefit of treatment at 12 weeks for completing the 4‐move solution problem. Participants completed the problem using fewer moves and had a shorter subsequent thinking time (*d* = 0.37 and *d* = 0.35, respectively). For Kennedy et al. (2001) and (2002), Cohen's *d* did not allow any further exploration of the data using our specified rule.

However, Appendix S3 (Cohen's *d*_z_) demonstrates the effect size of the significant effects reported in Kennedy et al. (2001) and (2002). For example, Kennedy et al. (2001)’s statistical test show a significant benefit of the 960 mg dose Gincosan^®^ over placebo at 1 and 6 hr after ingestion. Our calculations quantify the size of the effect: *d*_z_ = 7.92 (1 hr) and 4.69 (6 hr) for quality of memory, and 15.24 (1 hr) and 12.51 (6 hr) for secondary memory. We invite our readers to further explore our effect size results (see Appendix S2 and S3) in relation to the significant results of each publication reviewed in Section 3.2.

## 4 DISCUSSION

### 4.1 Summary of studies

The body of evidence related to the physiological and psychological effects of combining *Panax ginseng* and *Ginkgo biloba* into a single treatment is small and limited to studies that have assessed one commercially available combination product—Gincosan^®^. Despite this, eight studies utilizing some of the most robust and controlled methods/experimental designs have been discussed. From our review, we conclude that there are a small amount of data that have assessed the physiological effects; however, from these data there is direct evidence of positive effects upon the circulatory/cardiovascular system. With regard to psychological effects, we conclude that a *Panax ginseng*/*Ginkgo biloba* combination treatment can modulate cognitive function with the strongest and most consistent effect being one of improved “secondary memory” performance. This memory effect has been demonstrated in healthy populations and patient populations and to be present as early as 60 min after treatment ingestion and 14 days after treatment cessation. Two publications (Kennedy et al., 2002 and Scholey et al., 2002) provide direct evidence to suggest that a combination treatment can produce a stronger and more persistent effect than either *Panax Ginseng* (G115) or *Ginkgo biloba* (GK501) ingested alone. Further research is needed to understand the impact upon more complex cognitive processes and to further understand the impact upon attentional processes. It should be noted that the most recent study to investigate the effects of a combination treatment was published in 2004 and technological advances in the last 13 years are likely to make a significant difference—for example, relevant ambulatory physiological measurements for the cardiovascular system are now easily accessed as well as newly developed brain‐imaging techniques (e.g., fNIRS) and wearable‐technology advances. The next section will systematically discuss and expand on some of the issues we raised in Section 3 and also suggest improvements for future studies.

### 4.2 Evaluation of studies

Despite the high caliber of the reviewed Gincosan^®^ studies, we have identified a number of issues we believe to be important. Here, we discuss and make recommendations for each of the issues that can also be applied more generally to psychopharmacological research studies.

#### 4.2.1 Research model

**Figure 1:**
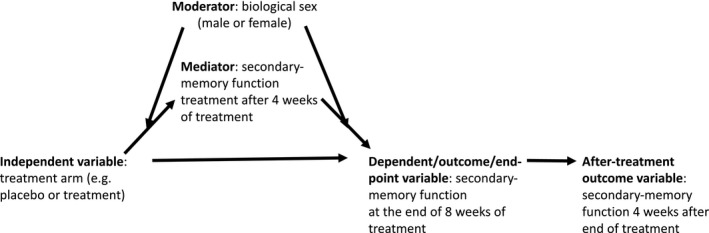
Illustrative research model

It is normally considered fundamental for any experimental research study to have an underlying research model. This is because by building and testing models, science can progress in a cumulative fashion (Jaccard & Jacoby, 2009). A research model specifies the variables that are studied and their (causal) relations. The self‐imposed discipline of developing a research model for each study forces researchers to carefully consider all the relevant variables and make explicit how these are related. These relations are then to be tested with the data that the study will produce. Such a model will at least include an independent variable (treatment) and a dependent variable (outcome). However, models can include additional variables as discussed in this section. Although none of the reviewed studies presents a research model, some studies (implicitly) indicate particular hypotheses to be tested (e.g., the potentially synergistic effect of Gincosan^®^; Scholey & Kennedy, 2002). In addition to independent and dependent variables, important variable types to consider in developing a research model include endpoint, mediator, and moderator. The model presented in Figure 1 illustrates these main concepts. According to the model, the (manipulated) independent variable treatment arm has an indirect effect on the dependent variable/endpoint secondary‐memory function at the end of 8 weeks of treatment. The effect is indirect because treatment arm has a direct effect on the mediator secondary‐memory function treatment after 4 weeks of treatment and, in turn, the mediator has a direct effect on the dependent variable. The effect of the independent variable on the mediator is moderated by biological sex. This means that the effect of treatment on the secondary‐memory function at 4 weeks differs between males and females. Moreover, the effect of the mediator on the endpoint is moderated by biological sex. Again, this means that the effect of secondary‐memory function at 4 weeks on secondary‐memory function at 8 weeks differs between males and females. Finally, secondary‐memory function at 8 weeks when treatment stops has an effect on secondary‐memory function at 12 weeks.

##### Endpoints

Some of the reviewed publications specify ultimate (primary) endpoint measures/outcomes of the effect of treatment with Gincosan^®^ on physiological and psychological outcome measures (Kennedy et al., 2001; Wesnes et al., 1997, 2000). Although this subset of three publications distinguish ultimate and other endpoint measures, the role of the other measures in a causal process (see below) is not examined and no theory is presented to support such a process.

The specification of a time endpoint is necessary to establish when a treatment effect should occur and should be measured to verify it does occur at the specified time. Otherwise, the effect of treatment cannot be established unambiguously. A model of the causal process can provide a justification for and strengthen the specification of this time endpoint. However, only one of the reviewed studies (Wesnes et al., 1997) provided a time endpoint, but without justification.

*Recommendations*. Researchers should specify one or more ultimate endpoint measures (see Figure 1) with a theoretical justification. They should also specify a time endpoint with each measure, with a (practical or theoretical) justification.

##### Mediators

A mediator is a variable that explains the effect of an independent variable on a dependent variable. MacKinnon (2008) highlights the importance of establishing the causal process (explaining why or how) an independent variable (through its effect on a mediator) influences a dependent variable. In the case of cognition‐enhancing drugs, biological (e.g., blood flow during a learning task) or behavioral (e.g., attention during a learning task) mediators may provide the explanation for the effect of treatment on ultimate endpoint measures (episodic‐memory performance). Moreover, the identification of intermediate variables can suggest additional alternative treatments on treatment elements to increase the effect on the ultimate endpoint.

Several of the reviewed studies (Kennedy et al., 2001, 2002; Scholey & Kennedy, 2002; Wesnes et al., 1997, 2000) include different time point measurements. For example, the data collected by Wesnes et al. (2000) present an opportunity to analyze mediation of the effect of treatment on a later measurement (Week 14) through the observed effect on an earlier measurement (Week 12), as treatment was not continued between the two times points. Therefore, in the reviewed research, data have been collected that could be, but have not been recognized as, mediators to provide insight into causal processes (see also MacKinnon, 2008). There are two types of theory in developing a mediation model to consider (Chen, 1990). First, action theory specifies how a treatment changes the mediating variable(s). Second, conceptual theory stipulates how the mediating variable(s) change the outcome variable.

*Recommendations*. Researchers should consider including potential intermediate endpoints (mediators; see Figure 1) in their study designs, as a vehicle for identifying causal processes underlying the treatment effect. They should justify their mediation model based on a specified action theory and a specified conceptual theory. Researchers should also consider using MacKinnon's mediation approach to developing treatment programs. Furthermore, they should consider using MacKinnon's (2008) (a) procedure for applying a mediation approach to developing treatment programs and (b) sources of ideas for mediators in treatment studies.

##### Moderators

A moderator is a variable that changes the direction and/or the size of the relationship between two variables (e.g., between treatment and outcome) and can explain when or under which conditions the relationship occurs. An important consideration is that the effect of treatment may depend on baseline scores of the dependent variable as a moderator (MacKinnon, 2008). Moderation happens frequently in treatment research, as those participants who make the biggest gain are frequently those who had the lowest baseline scores.^32^ Another example of a moderator variable is biological gender. For example, because of various genetic sex differences (Karp et al., 2017) female participants may derive more or less benefit from drug treatment than male participants. None of the reviewed publications addresses moderation to determine the conditions under which the effect of Gincosan^®^ occurs.

*Recommendations*. Researchers should consider including potential moderators (see Figure 1) in their research designs, as a vehicle for identifying conditions under which (a) a treatment effect occurs or (b) mediated effects on treatment occur.

#### 4.2.2 Research design

The internal and external validity is an essential consideration in the design of psychopharmacological experiments. This is because these types of validity restrict the soundness of the conclusions that can be drawn from these experiments.

##### Control of practice effect

Including a placebo run‐in phase in the research design allows control over potential practice effects and helps to minimize the placebo effect. Several of the reviewed studies included a placebo run‐in, while others included a practice day (in the absence of placebo treatment). However, no study provided any objective evidence to demonstrate the effectiveness of any run‐in/practice phase. In addition, even in studies utilizing the same protocol, the level of practice may have varied, as can perhaps most clearly be seen in a comparison of the baseline scores of Kennedy et al. (2001) and (2002), discussed in Section 3. This prohibits a meaningful comparison between studies in terms of treatment effect. This could be avoided by ensuring that each participant is practised at their optimal level of cognitive performance (beyond which practice does not lead to additional improvement without treatment) and then administer treatment to demonstrate any benefit beyond this level. Another limitation in one study (Hartley et al., 2004) was that practice took place immediately before baseline measurement, posing a potential threat to internal validity. In particular, baseline performance may have been subject to effects of boredom and/or fatigue.

*Recommendations*. A placebo run‐in phase should be included in each research design to control potential practice and placebo effects, which should take place on a different day before baseline testing. In addition, for meaningful comparison between studies, researchers should seriously consider practising participants to their optimal cognitive‐performance level before introducing treatment, and objectively measuring and reporting this phase.

##### Longevity of effect

Including a washout phase in the research design allows testing of longevity of a treatment effect and some studies included this during (Kennedy et al., 2001, 2002; Scholey & Kennedy, 2002) or after the treatment period (Wesnes et al., 2000). With the latter, any long‐lasting chronic effect can be tested, while with the former the continuation of an acute effect after discontinuation of treatment can be tested. With both, testing should continue until all effects have diminished. With regard to acute effects, research has tested 6 hr after the initial ingestion (but no longer) and chronic studies have tested 14 days after treatment cessation (but no longer); however, at these time points, positive effects of treatment were still demonstrated.

*Recommendations*. If the aim of the study is to demonstrate a long‐lasting chronic effect then the design should include a washout after the treatment period and testing should continue until treatment effects diminish. If the aim is to demonstrate a consistent acute effect of treatment, then the design should include a washout during the treatment period.

##### Testing of chronic effect versus chronic and acute effect

The time of testing in relation to the time of treatment (ingestion) determines the type of effect that can be measured. For those studies investigating repeated ingestion, testing before ingestion allows measuring a pure chronic effect (we assume the time of testing takes into consideration the half‐life of the compound under investigation). However, testing after ingestion allows measuring a combined chronic and acute effect (we assume the time of testing takes into consideration the biological availability of the compound under investigation).

*Recommendations*. First, if the aim is to measure a pure chronic effect, then test before ingestion. Second, if the aim is to measure the combined chronic and acute effect, then test after ingestion. In both cases, appropriate data analysis is required to achieve the aim (see below).

##### Interaction effects

Different types of interaction effect that were found in the reviewed studies have different implications for internal validity, in other words conclusions that can be drawn from treatment studies. An interaction effect between task difficulty/mental effort and treatment (Kennedy et al., 2002; Kwiecinski et al., 1997) indicates that the treatment effect may be demonstrated at some levels of task difficulty, but not at other levels. For example, Kwiecinski et al. (1997) found a treatment effect on a more difficult version of visual scanning task, but not on an easier version. For another example, Kennedy et al. (2002) found a stronger treatment effect on the more complex version of a mental‐arithmetic task. Therefore, if the “wrong” task difficulty level (e.g., too easy) is chosen then a treatment effect cannot be established.

An interaction effect between dose and task domain (Kennedy et al., 2001; Wesnes et al., 1997) indicates that the treatment effect may be demonstrated for some tasks, but not for others. For example, Wesnes et al., 1997 found that a 160 mg dose of Gincosan^®^ improved the speed of memory task performance, but a 320 mg dose improved memory accuracy. For another example, in Kennedy et al.’s (2001) results, memory performance (but not attentional processing) was enhanced by Gincosan^®^. Consequently, if the “wrong” task is selected then a treatment effect cannot be established.

The synergistic effect of Gincosan over and above the separate effects of ginkgo and ginseng may differ across outcome measures. For example, Kennedy et al. (2002) established that relative to the separate components *Ginkgo biloba* and *Panax ginseng*, Gincosan^®^ (as the combination of ginkgo and ginseng) outperformed placebo in particular for quality of memory and secondary memory. However, this was not the case for most other outcome measures. Therefore, the synergistic effect of Gincosan^®^ can be demonstrated if appropriate outcome measures are selected.

*Recommendations*. Interaction effects need to be carefully considered in the design of studies, for example by selecting an appropriate level of task difficulty in relation to treatment, task domain in relation to treatment dose, and outcome measure(s) in relation to a synergistic effect of Gincosan^®^. Hartley et al.’s (2004) research provides a further example; they failed to find any effect, perhaps because they did not consider interaction effects.

##### Measurement of cognitive function

When standardized validated measures of cognitive task performance are used the behavior that is being measured is clearly defined; examples include the CDR cognitive‐test battery (e.g., Wesnes et al., 1997; Wesnes et al., 2000) and CANTAB. However, this is not necessarily true when nonstandardized nonvalidated measures are used (e.g., Kennedy et al., 2001, 2002; Scholey & Kennedy, 2002).

Furthermore, measures of sub‐factors within higher‐order cognitive functions can be used to pinpoint specific component functions that demonstrate treatment effect. For example, Kennedy et al. (2001) showed that of two components of quality of memory (secondary memory and working memory) there was a treatment effect on the former, but not on the latter. More generally, the selection of cognitive task domain to measure is important, as a treatment effect can only be established on task domains that respond to experimental manipulation (e.g., secondary memory). For example, Hartley et al. (2004) did not measure secondary‐memory performance as an outcome although previous research showed that Gincosan^®^ enhanced this outcome.

*Recommendations*. The choice of task to measure cognitive function needs to be carefully considered in study design. In particular, validated measures and specific sensitive measures should be selected.

##### Manipulation of treatment

One way to provide more precision regarding the effect of Gincosan^®^ or other cognition‐enhancing drugs is to vary the dose and observe resulting differences in cognitive function. This research can help establish the required or optimal dose to achieve improvements in cognitive function. For example, Scholey and Kennedy (2002) studied the dose‐response effect (with different doses) of Gincosan^®^ and its two separate components, whereas Kennedy et al. (2002) studied only one dose for each drug.

*Recommendations*. Researchers who want to contribute to knowledge for guiding the selection of treatment dose should consider using a dose‐response design.

##### Time of testing (main effect)

Consistently using the same time window of testing (e.g., within a week) increases internal validity. By contrast, a lack of consistency (e.g., as in Kwiecinski, 1997) is a threat to internal validity and increases error variance, and thereby reduces statistical power.

Another consideration is experimental control over postdose time of testing on testing day. This is important because (a) post‐dose time of testing interacts with treatment and (b) postdose time interval interacts with time of day. A lack of this type of control (as in Hartley et al., 2004) presents a threat to internal validity.

*Recommendations*. For the sake of internal validity, researchers should use study designs that consistently use the same time (e.g., weekly) window of testing and postdose time of testing. Researchers should also consider conducting replication studies to establish the consistency of findings (Hornbæk, Sander, Bargas‐Avila, & Grue Simonsen, 2014), in particular if the original findings are surprising.

##### Effect of sample characteristics

Sample characteristics can influence the outcome over and above the treatment effect. For example, Hartley et al. (2004) selected post‐menopausal women as participants, but this group is known for having a reduced level of memory performance, most likely due to changing hormone levels (e.g., estrogen) and there is some evidence to suggest that ginkgo has estrogenic properties. For a second example, the variability in post‐menopausal status (early and late) in Hartley et al.’s (2004) participants creates additional variability in test outcomes. In general, sample characteristics that are not carefully controlled create confounds of the treatment effect. For third example, recent research (Karp et al., 2017) suggests that because of gender differences in genetic function, the effect of drug treatments may differ between males and females; this has potentially significant implications for psychopharmacology in terms of finding the most effective potentially sex‐specific treatment to (optimally) boost cognitive performance.

*Recommendation*. Researchers should control sample characteristics by providing a credible rationale for their selection of participants, recruiting homogeneous samples and otherwise measuring any individual‐difference variables that are associated with the outcome measure(s) and that can be used as covariates or mediators in data analysis.

#### 4.2.3 Data analysis

In addition to the choice of research design, the choice of data analysis imposes further restrictions on the conclusions that can be drawn from psychopharmacological experiments.

##### Match between research model, research design and data analysis

The research design can be considered a more detailed specification of the research model. Moreover, data analysis normally tests the model (as a whole or specific relationships in the model). None of the reviewed publications presents a research model, so consistency between research design and research model and between data analysis and research model cannot be established.

*Recommendation*. Researchers need to ensure their research design is consistent with their research model (e.g., a model with independent variables, mediators, and outcomes requires a research design in which all of these are operationalized) and their data analysis is consistent with their research model (e.g., a mediation model requires mediation analysis).

##### Inconsistency in data analysis across studies

Research designs and data analysis strategies differ across studies. This can make the comparison of results between studies problematic. For example, some studies report a priori comparisons with mean‐square terms from an omnibus test (Kennedy et al., 2001, 2002; Scholey & Kennedy, 2002; Wesnes et al., 1997), while other studies report a less powerful analysis with omnibus tests and follow‐up a priori or unplanned comparisons (Wesnes et al., 2000) or only report a *p*‐value (Kwiecinski et al., 1997).

*Recommendation*. Researchers should keep statistical analysis consistent or refer to common terms that allow some comparison (e.g., effect size; see below). They should publish their data anonymized to facilitate further or alternative data analysis. First, this will allow the data of different studies potentially to be analyzed more appropriately or consistently, and more fully than is reported in the original publications. Second, the additional or new results from this can be used to conduct meta‐analyses to go beyond the individual studies through statistical inference.

##### Between‐subjects tests versus within‐subjects tests

Between‐subjects tests analyze differences between groups after treatment, while within‐subjects tests analyze improvement within a group, even if the group performance was worse than other groups. Some publications report within‐subjects tests of improvements within a treatment group to demonstrate the effect of treatment. However, such improvements may not be meaningful if, despite such an improvement, in a between‐subjects test the treatment group's cognitive performance does not significantly differ from the placebo condition.

*Recommendation*. Within‐group improvements should be interpreted in the context of between‐group differences in cognitive performance. More generally, analysis of differences in endpoint performance should be tested (e.g., using analysis of covariance) rather than within‐group improvements to demonstrate a treatment effect (Dimitrov & Rumrill, 2003).

##### Effect size

Any meaningful assessment of treatment effect needs to include an analysis of effect size. However, none of the studies report standardized effect sizes that can be compared across studies. Some implicitly report nonstandardized effect sizes (change from baseline; Kennedy et al., 2003, 2001; Scholey & Kennedy, 2002; Wesnes et al., 1997, 2000), but do not use the term effect size and do not interpret the achieved effect size.

*Recommendation*. Researchers should report the measured effect sizes of their studies, preferably using a standardized effect size that allows comparisons across studies. A good candidate for the research studies analyzed in this review (and potentially for other psychopharmacological studies) is the standardized difference (e.g., Cohen's *d*), for instance to compare treatment with placebo (see also Section 3.3 above).

##### Alternative to null‐hypothesis significance testing

Effect size is not only important to quantify the extent of the treatment effect as measured effect size, the desired effect size should also be an integral part of inferential data analysis. In standard null‐hypothesis significance testing, the “desired” effect size against which the data are tested is an unrealistic null effect (e.g., no difference between treatment and placebo). As a result, the inference is an artifact of sample size: if sample size is large enough the inference result will be significant. We and others have proposed magnitude‐based inference as an attractive alternative (Buchheit, 2016; Hopkins & Batterham, 2016; van Schaik & Weston, 2016) that uses the smallest worthwhile positive/beneficial effect and worthwhile negative/harmful effect as part of the inference that is made. As a result, the inference from magnitude‐based inference is never an artifact of sample size. Instead, there are two possible outcomes. First, the result is clear and is then qualified as trivial/negligible, positive/beneficial, or negative/harmful, with a qualitatively described level of probability (Van Schaik et al., 2016, Tables S1–S3; Figure 1). Second the result is unclear, with the need to collect more data until a clear result is obtained. A related approach that takes into account the smallest worthwhile effect is the use of minimum‐effect tests (Murphy & Myors, 1999). Other alternatives to null‐hypothesis significance testing exist, such as Bayesian tests. However, these have their drawbacks. For example, providing believable estimates of prior beliefs that these tests require is considered a major obstacle (Bland & Altman, 1998). Moreover, there is doubt about the accessibility, comprehensibility, and usability of this approach for researchers (Hopkins, 2006).

*Recommendation*. Researchers should include the smallest worthwhile effect as an integral part of their inferential data analysis. They should consider using magnitude‐based inference as a way to achieve this.

##### Mediation analysis

Mediation analysis can be used to provide evidence for the causal process (why or how) of the treatment effect (Hayes, 2013; MacKinnon, 2008). Although the application of mediation analysis could have been appropriate (e.g., Wesnes et al., 2000), none of the reviewed publications reports the use of this.

*Recommendations*. Researchers who have identified mediators in their research model should conduct mediation analysis on their data.

##### Moderation analysis

Moderation analysis can be used to provide evidence the conditions under which (when) a treatment effect exists (Hayes, 2013). First, moderation analysis allows researchers to establish whether baseline score moderates effect of treatment on the outcome by (the conditions under which the [unmediated] effect occurs). Second, moderated mediation analysis (“conditional process analysis”; Hayes, 2017) allows them to establish whether the mediated effect of treatment is moderated by baseline score (the conditions under which mediation occurs). None of the reviewed publications reports moderation analysis or mediated‐moderation analysis. Although Hartley et al. (2004) report post‐hoc sub‐group analyses, these do not tell us whether subgroup membership moderates the effect of treatment.

*Recommendations*. Researchers who have identified moderators in their research model should conduct moderation analysis on their data. Researchers who have identified mediators whose effects are moderated should conduct moderated‐mediation analysis on their data.

## 5 CONCLUSION

Clinical trials that assess the impact of herbal supplements often suffer from poor design and heterogeneous methods making interpretation of clinical efficacy difficult. The clinical literature is replete with systematic reviews demonstrating the high volume of poorly designed trials that have not made it beyond selection criteria, but, in isolation, have been published as original research papers. The current systematic review has detailed the clinical evidence for combining *Panax ginseng* and *Ginkgo biloba* into a single treatment. All studies utilized Gincosan^®^ and we conclude that the trials are robust and well designed. With regard to physiological effects, we conclude that there is clear evidence of modulation of the circulatory/cardiovascular system in samples with nonoptimal performance of this system. With regard to psychological effects, we conclude that collectively the trials clearly show that Gincosan^®^ can improve aspects of memory, most notably secondary memory following acute and repeated ingestion in patient and healthy populations. There is evidence to show that a single dose of Gincosan^®^ can improve aspects of cognitive functioning beyond memory processes; however, this effect needs replication following repeated dosing. Finally, there is evidence to show that Gincosan^®^ produces a pattern of result indicative of a synergistic relationship between its constituent parts. Clearly, taken together this evidence suggests Gincosan^®^ may have great benefit to a healthy population and patient population that suffer memory problems and that research should further explore the benefits of combining *Panax ginseng* and *Ginkgo biloba* into a single treatment. Our review also demonstrates that in terms of research model, research design, and data analysis, the reviewed studies have various important limitations that restrict the conclusions that can be drawn. In response, we have provided guidance for creating better‐quality future psychopharmacological research studies.

## Note

1 If a body of evidence accumulates, using the same standardised extract and is homogenous in the study design to allow a more general research question to be addressed, then a meta‐analytical approach will allow a more reliable estimate of the effect size

2 The main reason being that each herb will contain numerous (>30) individual biologically active chemicals (potentially, producing more when metabolised) interacting with numerous biological pathways.

3 and perhaps the proportion and each biologically active chemical within each extract.

4 If abstracts were poorly written and did not contain enough information to assess the study against the inclusion criteria, the method section was scrutinised.

5 Mean and standard deviations (or standard errors and sample size) are necessary to calculate the effect size. A number of the papers do not report this information.

6 Made from the roots of *Panax ginseng C.A. Meyer*, adjusted to 4% ginsenosides.

7 Made from the leaves of *Ginkgo biloba* L., adjusted to 24.5% ginkgoflavon glycosides and 6% terpenes (ginkgolides, bilobalide).

8 Treatment dose absent on day of testing

9 Treatment dose present on day of testing

10 The effect was limited to this one clinical symptom outcome, from a maximum of nine (1. Dizziness, 2. Tinnitus, 3. Headache, 4. Day‐time irritability, 5. Night‐Time restlessness, 6. Lack of concentration and forgetfulness, 7. Depressive mood, felling of rejection, 8. Narrowed interests, 9. Social withdrawal).

11 Baseline placebo (526.8 s) versus baseline treatment (555.2 s): 8 week placebo (508.9 s) versus treatment (531.1 s).

12 One of the herbal extracts making Gincosan^®^. The other being *Panax ginseng.*

13 This is an important observation, as when combining multiple treatments, it is important to consider their interaction. This is particularly true of herb/herb interaction as this is an under researched area

14 The previous study by Kiesewetter et al. (1992) highlighted physiological changes following one single dose 60 min after ingestion.

15 Participants cycled on an ergometer for 8 min

16 The quality‐of‐memory index was derived from the overall percentage accuracy scores from the numeric working memory task, the immediate word recall task, the delayed word recall, the delayed word recognition, and the delayed picture recognition task (scores were summed)

17 Although the latter was evident on the accuracy score for the quality‐of‐memory index and the former on the speed of performing those computerised tasks used to generate the composite quality‐of‐memory index score.

18 Interestingly, this task does not draw heavily upon memory processes or require physical effort

19 mean placebo (13.67) versus mean treatment (13.71)

20 These outcome variables are synonymous with those reported by Wesnes et al. (2000) and Wesnes et al. (1997) albeit using slightly amended name descriptors. In particular, quality of memory was adjusted from quality‐of‐memory index; power of attention was changed to speed of attention; continuity of attention was changed to accuracy of attention.

21 Notwithstanding the obvious differences in study population and protocol across the three studies.

22
*Ginkgo biloba *and *Panax ginseng*

23 However, it should be noted that the result also show, for the first time, improved performance of the working memory sub‐factor; however, the authors state that caution should be taken with this novel finding as their results revealed a trend toward baseline differences on one of the tasks used to calculate the composite score

24 This effect is present at the same time point in patient populations too (see Wesnes et al., 1997).

25 as well as cohort specific individual differences and slight variation in the active ingredients

26 If a base level cannot be achieved because task performance will always improve with subsequent task completion, then random allocation and counterbalancing will go some way to control for this.

27 Interaction effects between task complexity and treatment efficacy are now commonly reported.

28 There was no effect on alertness or calmness.

29 Research published in the same year reported a treatment by menopausal stage interaction (e.g. Elsabagh, 2004a).

30 Hartley(2004) also acknowledge this in their own paper (P329).

31 planning task from CANTAB

32 However, it should be noted that a low baseline score may be an artefact of regression toward the mean. If it is important to rule out this possibility then two (or more) baseline measurements may be taken to establish stability.
