# Supplementary material for: A systematic review of research investigating the physiological and psychological effects of combining Ginkgo biloba and Panax ginseng into a single treatment in humans: Implications for research design and analysis
Source: Brain Behav. 2019 Feb 6;9(3):e01217. doi: 10.1002/brb3.1217 (PMC6422825; doi:10.1002/brb3.1217)
Supplement: Supplementary file 1 [file BRB3-9-e01217-s001.docx]

Appendix 2: For Hartley et al., (2004), Kennedy et al., (2001) and (2002), we report in Tables 1 to 48, the standardized effect size Cohen’s *d*. For Hartley et al., (2004) our Cohen’s *d* calculation is related to the primary statistical analysis conducted in the paper. For Kennedy et al., (2001) and (2002) our Cohen’s *d* calculation is related to ‘absolute’ scores (the original papers report change from baseline rather than absolute score – please see appendix 2 for standardized effect size calculations related to change from baseline analysis reported in Kennedy et al., 2001 and 2002). Table 1 to Table 4; 17 to 22 and 33 to 38 show Cohen’s *d* calculations for the effect of dose (Gincosan^®^ v Placebo) at different time points (Weeks 0, 6 or 12) and Table 5 to Table 16; 23 to 32 and 39 to 48 show Cohen’s *d* calculations for the effect of time (e.g. Week 6 versus Week 0) within each dose (Gincosan^®^ or Placebo). Each table reports the mean difference (labeled, Mean), the *d* value and the lower and upper confidence interval of the *d* value, at each assessment point for each outcome measure. As an example, in Table 1 we can see the mean difference between placebo and Gincosan at the baseline assessment point, for the Psychological outcome measure is 1.5. This figure is derived as follows: in the original Hartley et al., (2004) paper, the placebo score was 5.9 and the Gincosan score was 7.4 (therefore, 7.4 – 5.9 =1.5). The below tables, now allow the magnitude of difference to be observed. In the above example, this would equate to *d* =0.39.

Table 1. Hartley et al., (2004) psychological measures: comparing Placebo and Gincosan at different time points

|  | Baseline | | | | | 6 Weeks | | | | 12 weeks | | | |
| --- | --- | --- | --- | --- | --- | --- | --- | --- | --- | --- | --- | --- | --- |
|  | Mean | *d* | LCI | UCI | Mean | *d* | LCI | UCI | Mean | | *d* | LCI | UCI |
| Psychological | 1.5 | **0.39** | *-0.15* | *0.93* | 1.6 | **0.37** | *-0.17* | *0.90* | 1.9 | | **0.58** | *0.04* | *1.12* |
| Somatic | 0.4 | **0.24** | *-0.29* | *0.78* | 0.2 | **0.12** | *-0.41* | *0.65* | 0.2 | | **0.12** | *-0.41* | *0.65* |
| Vasomotor | -0.1 | **-0.09** | *-0.62* | *0.44* | -0.1 | **-0.09** | *-0.62* | *0.44* | -0.2 | | **-0.18** | *-0.72* | *0.35* |
| Sexual Dysfunction | 0.5 | **0.46** | *-0.08* | *0.99* | 0.5 | **0.46** | *-0.08* | *0.99* | 0.4 | | **0.37** | *-0.17* | *0.90* |
| HAD (anxiety) | 1.8 | **0.47** | *-0.07* | *1.01* | 0.5 | **0.11** | *-0.42* | *0.65* | 0.9 | | **0.27** | *-0.26* | *0.81* |
| HAD (depression) | 1.1 | **0.50** | *-0.04* | *1.04* | 1.1 | **0.50** | *-0.04* | *1.04* | 0.7 | | **0.43** | *-0.11* | *0.96* |
| Epworth Scale | 0.2 | **0.05** | *-0.48* | *0.58* | -0.4 | **-0.08** | *-0.61* | *0.45* | 0.4 | | **0.09** | *-0.44* | *0.62* |

Table 2. Hartley et al., (2004) psychological measures 2: comparing Placebo and Gincosan at different time points

|  |  | Baseline | | | | 6 Weeks | | | | 12 weeks | | | |
| --- | --- | --- | --- | --- | --- | --- | --- | --- | --- | --- | --- | --- | --- |
|  |  | Mean | *d* | LCI | UCI | Mean | *d* | LCI | UCI | Mean | *d* | LCI | UCI |
| Alertness | Pre | 2.6 | **0.24** | *-0.30* | *0.77* | 2.2 | **0.21** | *-0.32* | *0.74* | 0.7 | **0.07** | *-0.46* | *0.60* |
|  | Post | -2.1 | **-0.18** | *-0.72* | *0.35* | -0.3 | **-0.02** | *-0.56* | *0.51* | 1.1 | **0.08** | *-0.45* | *0.62* |
| Well-being | Pre | -1.1 | **-0.10** | *-0.63* | *0.44* | 0.7 | **0.07** | *-0.46* | *0.60* | -1.6 | **-0.19** | *-0.73* | *0.34* |
|  | Post | -2.5 | **-0.21** | *-0.74* | *0.33* | -3.8 | **-0.28** | *-0.81* | *0.26* | 1.4 | **0.11** | *-0.43* | *0.64* |
| Anxiety | Pre | -3.0 | **-0.18** | *-0.71* | *0.36* | -4.1 | **-0.28** | *-0.81* | *0.26* | -1.4 | **-0.09** | *-0.63* | *0.44* |
|  | Post | 0.6 | **0.04** | *-0.50* | *0.57* | 1.6 | **0.08** | *-0.45* | *0.61* | 1.1 | **0.05** | *-0.48* | *0.58* |
| Stanford Scale | Pre | -0.1 | **-0.09** | *-0.62* | *0.44* | 0.0 | **0.00** | *-0.53* | *0.53* | -0.2 | **-0.18** | *-0.72* | *0.35* |
|  | Post | 0.0 | **0.00** | *-0.53* | *0.53* | 0.3 | **0.27** | *-0.26* | *0.81* | 0.1 | **0.09** | *-0.44* | *0.62* |

Table 3. Hartley et al., (2004) memory and attention measures: comparing Placebo and Gincosan at different time points

|  | Baseline | | | | 6 Weeks | | | | 12 weeks | | | |
| --- | --- | --- | --- | --- | --- | --- | --- | --- | --- | --- | --- | --- |
|  | Mean | *d* | LCI | UCI | Mean | *d* | LCI | UCI | Mean | *d* | LCI | UCI |
| Picture Recall | 1.7 | **0.62** | *0.08* | *1.16* | 0.5 | **0.30** | *-0.23* | *0.84* | 1.3 | **0.79** | *0.24* | *1.34* |
| Story Recall Immediate | -0.7 | **-0.13** | *-0.66* | *0.40* | -1.5 | **-0.46** | *-0.99* | *0.08* | -1.1 | **-0.29** | *-0.82* | *0.25* |
| Story Recall Delayed | -0.9 | **-0.16** | *-0.70* | *0.37* | -1.6 | **-0.49** | *-1.03* | *0.05* | -1.9 | **-0.50** | *-1.04* | *0.04* |
| Verbal Fluency | -0.5 | **-0.13** | *-0.66* | *0.40* | -0.4 | **-0.10** | *-0.64* | *0.43* | 0.2 | **0.04** | *-0.49* | *0.57* |
| DTMS Errors | 0.9 | **0.55** | *0.01* | *1.09* | 0.3 | **0.18** | *-0.35* | *0.72* | 0.1 | **0.09** | *-0.44* | *0.62* |
| DTMS L Sim | 394.0 | **0.64** | *0.09* | *1.18* | 287.0 | **0.52** | *-0.02* | *1.06* | 294.0 | **0.41** | *-0.13* | *0.94* |
| DTMS L 0 | -16.0 | **-0.02** | *-0.55* | *0.51* | 293.0 | **0.43** | *-0.10* | *0.97* | 310.0 | **0.50** | *-0.04* | *1.04* |
| DTMS L 4 | -53.0 | **-0.05** | *-0.58* | *0.48* | 383.0 | **0.46** | *-0.08* | *1.00* | 90.0 | **0.09** | *-0.44* | *0.62* |
| DTMS L 12 | 466.0 | **0.48** | *-0.06* | *1.01* | 70.0 | **0.07** | *-0.46* | *0.61* | 201.0 | **0.17** | *-0.36* | *0.71* |
| PASAT 1.5 | -0.3 | **-0.03** | *-0.56* | *0.51* | -0.7 | **-0.07** | *-0.60* | *0.46* | -1.6 | **-0.15** | *-0.69* | *0.38* |
| PASAT 1.2 | 0.1 | **0.01** | *-0.52* | *0.54* | -0.1 | **-0.01** | *-0.54* | *0.52* | 0.2 | **0.02** | *-0.51* | *0.55* |

Table 4. Hartley et al., (2004) frontal lobe cognition measures: comparing Placebo and Gincosan at different time points

|  | Baseline | | | | 6 Weeks | | | | 12 weeks | | | |
| --- | --- | --- | --- | --- | --- | --- | --- | --- | --- | --- | --- | --- |
|  | Mean | *d* | LCI | UCI | Mean | *d* | LCI | UCI | Mean | *d* | LCI | UCI |
| Soc4 moves | -0.1 | **-0.09** | *-0.62* | *0.44* | 0.0 | **0.00** | *-0.53* | *0.53* | -0.4 | **-0.37** | *-0.90* | *0.17* |
| Soc4 iTime | 702.0 | **0.14** | *-0.40* | *0.67* | 1617.0 | **0.40** | *-0.13* | *0.94* | 323.0 | **0.10** | *-0.43* | *0.63* |
| Soc4 sTime | 116.0 | **0.07** | *-0.46* | *0.60* | 410.0 | **0.23** | *-0.31* | *0.76* | -586.0 | **-0.35** | *-0.89* | *0.18* |
| Soc5 moves | 0.5 | **0.30** | *-0.23* | *0.84* | -0.1 | **-0.06** | *-0.59* | *0.47* | -0.4 | **-0.24** | *-0.78* | *0.29* |
| Soc5 iTime | 449.0 | **0.07** | *-0.46* | *0.60* | 1123.0 | **0.19** | *-0.34* | *0.72* | -290.0 | **-0.06** | *-0.59* | *0.47* |
| Soc5 sTime | 437.0 | **0.22** | *-0.32* | *0.75* | 74.0 | **0.06** | *-0.47* | *0.60* | -287.0 | **-0.19** | *-0.72* | *0.35* |
| IDED Trials | 7.4 | **0.33** | *-0.21* | *0.86* | 3.4 | **0.17** | *-0.36* | *0.70* | 4.6 | **0.29** | *-0.24* | *0.82* |
| IDED Errors | 4.7 | **0.39** | *-0.15* | *0.93* | 0.8 | 0.06 | *-0.47* | *0.60* | 2.0 | **0.18** | *-0.35* | *0.72* |

Table 5. Hartley et al., (2004) psychological measures: comparing effect sizes between Baseline and 6 weeks

|  | Placebo | | | | Gincosan | | | |
| --- | --- | --- | --- | --- | --- | --- | --- | --- |
|  | Mean | d | LCI | UCI | Mean | d | LCI | UCI |
| Psychological | 0.3 | **0.07** | *-0.44* | *0.59* | 0.4 | **0.08** | *-0.46* | *0.63* |
| Somatic | -0.3 | **-0.18** | *-0.70* | *0.34* | -0.5 | **-0.18** | *-0.73* | *0.37* |
| Vasomotor | 0.2 | **0.18** | *-0.34* | *0.70* | 0.2 | **0.11** | *-0.44* | *0.66* |
| Sexual Dysfunction | -0.2 | **-0.18** | *-0.70* | *0.34* | -0.2 | **-0.19** | *-0.74* | *0.35* |
| HAD (anxiety) | 0.5 | **0.12** | *-0.40* | *0.64* | -0.8 | **-0.19** | *-0.74* | *0.35* |
| HAD (depression) | 0.0 | **0.00** | *-0.52* | *0.52* | 0.0 | **0.00** | *-0.55* | *0.55* |
| Epworth Scale | 0.2 | **0.05** | *-0.47* | *0.56* | -0.4 | **-0.11** | *-0.66* | *0.44* |

Table 6. Hartley et al., (2004) psychological measures: comparing effect sizes between Baseline and 12 weeks

|  | Placebo | | | | Gincosan | | | |
| --- | --- | --- | --- | --- | --- | --- | --- | --- |
|  | Mean | *d* | LCI | UCI | Mean | *d* | LCI | UCI |
| Psychological | -0.5 | **-0.14** | *-0.66* | *0.38* | -0.1 | **-0.02** | *-0.57* | *0.53* |
| Somatic | -0.4 | **-0.24** | *-0.76* | *0.28* | -0.6 | **-0.20** | *-0.75* | *0.34* |
| Vasomotor | 0.2 | **0.18** | *-0.34* | *0.70* | 0.1 | **0.06** | *-0.48* | *0.61* |
| Sexual Dysfunction | -0.1 | **-0.09** | *-0.61* | *0.43* | -0.2 | **-0.19** | *-0.74* | *0.35* |
| HAD (anxiety) | -0.3 | **-0.08** | *-0.60* | *0.43* | -1.2 | **-0.29** | *-0.84* | *0.26* |
| HAD (depression) | -0.1 | **-0.05** | *-0.57* | *0.47* | -0.5 | **-0.17** | *-0.72* | *0.37* |
| Epworth Scale | -0.4 | **-0.10** | *-0.61* | *0.42* | -0.2 | **-0.06** | *-0.61* | *0.48* |

Table 7. Hartley et al., (2004) psychological measures: comparing effect sizes between 6 weeks and 12 weeks

|  | Placebo | | | | Gincosan | | | |
| --- | --- | --- | --- | --- | --- | --- | --- | --- |
|  | Mean | *d* | LCI | UCI | Mean | *d* | LCI | UCI |
| Psychological | -0.8 | **-0.21** | *-0.72* | *0.31* | -0.5 | **-0.09** | *-0.63* | *0.46* |
| Somatic | -0.1 | **-0.06** | *-0.58* | *0.46* | -0.1 | **-0.05** | *-0.60* | *0.49* |
| Vasomotor | 0.0 | **0.00** | *-0.52* | *0.52* | -0.1 | **-0.05** | *-0.60* | *0.49* |
| Sexual Dysfunction | 0.1 | **0.09** | *-0.43* | *0.61* | 0.0 | **0.00** | *-0.55* | *0.55* |
| HAD (anxiety) | -0.8 | **-0.21** | *-0.72* | *0.31* | -0.4 | **-0.10** | *-0.64* | *0.45* |
| HAD (depression) | -0.1 | **-0.05** | *-0.57* | *0.47* | -0.5 | **-0.17** | *-0.72* | *0.37* |
| Epworth Scale | -0.6 | **-0.13** | *-0.65* | *0.39* | 0.2 | **0.05** | *-0.49* | *0.60* |

Table 8. Hartley et al., (2004) psychological measures 2: comparing effect sizes between Baseline and 6 weeks

|  | Placebo | | | | | | | | Gincosan | | | | | | | |
| --- | --- | --- | --- | --- | --- | --- | --- | --- | --- | --- | --- | --- | --- | --- | --- | --- |
|  | Pre | | | | Post | | | | Pre | | | | Post | | | |
|  | Mean | *d* | LCI | UCI | Mean | *d* | LCI | UCI | Mean | *d* | LCI | UCI | Mean | *d* | LCI | UCI |
| Alertness | -1.3 | **-0.12** | *-0.64* | *0.40* | 3.8 | **0.32** | *-0.20* | *0.84* | -1.7 | **-0.15** | *-0.70* | *0.40* | 5.6 | **0.47** | *-0.09* | *1.02* |
| Wellbeing | -1.3 | **-0.12** | *-0.64* | *0.40* | 4.4 | **0.34** | *-0.18* | *0.86* | 0.5 | **0.04** | *-0.50* | *0.59* | 3.1 | **0.21** | *-0.33* | *0.76* |
| Anxiety | -4.0 | **-0.25** | *-0.77* | *0.27* | -6.2 | **-0.33** | *-0.85* | *0.19* | -5.1 | **-0.36** | *-0.91* | *0.19* | -5.2 | **-0.32** | *-0.87* | *0.23* |
| Stanford Scale | 0.1 | **0.09** | -0.43 | 0.61 | -0.4 | **-0.37** | *-0.89* | *0.16* | 0.2 | **0.19** | *-0.35* | *0.74* | -0.1 | **-0.10** | *-0.64* | *0.45* |

Table 9. Hartley et al., (2004) psychological measures 2: comparing effect sizes between Baseline and 12 weeks

|  | Placebo | | | | | | | | Gincosan | | | | | | | |
| --- | --- | --- | --- | --- | --- | --- | --- | --- | --- | --- | --- | --- | --- | --- | --- | --- |
|  | Pre | | | | Post | | | | Pre | | | | Post | | | |
|  | Mean | *d* | LCI | UCI | Mean | *d* | LCI | UCI | Mean | *d* | LCI | UCI | Mean | *d* | LCI | UCI |
| Alertness | 1.0 | **0.09** | *-0.42* | *0.61* | 7.1 | **0.57** | *0.05* | *1.10* | -0.9 | **-0.08** | *-0.63* | *0.47* | 10.3 | **0.84** | *0.27* | *1.41* |
| Wellbeing | 2.9 | **0.29** | *-0.23* | *0.81* | 6.6 | **0.52** | *0.00* | *1.05* | 2.4 | **0.23** | *-0.32* | *0.78* | 10.5 | **0.77** | *0.21* | *1.34* |
| Anxiety | -6.5 | **-0.41** | *-0.93* | *0.11* | -8.9 | **-0.46** | *-0.98* | *0.06* | -4.9 | **-0.39** | *-0.94* | *0.16* | -8.4 | **-0.49** | *-1.04* | *0.07* |
| Stanford Scale | 0.1 | **0.09** | *-0.43* | *0.61* | -0.2 | -0.18 | *-0.70* | *0.34* | 0.0 | **0.00** | *-0.55* | *0.55* | -0.1 | **-0.10** | *-0.64* | *0.45* |

Table 10. Hartley et al., (2004) psychological measures 2: comparing effect sizes between 6 weeks and 12 weeks

|  | Placebo | | | | | | | | Gincosan | | | | | | | |
| --- | --- | --- | --- | --- | --- | --- | --- | --- | --- | --- | --- | --- | --- | --- | --- | --- |
|  | Pre | | | | Post | | | | Pre | | | | Post | | | |
|  | Mean | *d* | LCI | UCI | Mean | *d* | LCI | UCI | Mean | *d* | LCI | UCI | Mean | *d* | LCI | UCI |
| Alertness | 2.3 | **0.22** | *-0.30* | *0.74* | 3.3 | **0.26** | *-0.26* | *0.78* | 0.8 | **0.07** | *-0.48* | *0.61* | 4.7 | **0.38** | *-0.17* | *0.94* |
| Wellbeing | 4.2 | **0.46** | *-0.06* | *0.99* | 2.2 | **0.16** | *-0.35* | *0.68* | 1.9 | **0.16** | *-0.38* | *0.71* | 7.4 | **0.57** | *0.01* | *1.12* |
| Anxiety | -2.5 | **-0.17** | *-0.69* | *0.35* | -2.7 | **-0.13** | *-0.65* | *0.39* | 0.2 | **0.02** | *-0.53* | *0.56* | -3.2 | **-0.18** | *-0.73* | *0.37* |
| Stanford Scale | 0.0 | **0.00** | *-0.52* | *0.52* | 0.2 | **0.18** | *-0.34* | *0.70* | -0.2 | **-0.19** | *-0.74* | *0.35* | 0.0 | 0.00 | *-0.55* | *0.55* |

Table 11. Hartley et al., (2004) memory and attention measures: comparing effect sizes between Baseline and 6 weeks

|  | Placebo | | | | Gincosan | | | |
| --- | --- | --- | --- | --- | --- | --- | --- | --- |
|  | Mean | *d* | LCI | UCI | Mean | *d* | LCI | UCI |
| Picture Recall | 2.4 | **1.06** | *0.51* | *1.61* | 1.2 | **0.42** | *-0.13* | *0.97* |
| Story Recall Immediate | 3.0 | **0.66** | *0.13* | *1.20* | 2.2 | **0.56** | *0.01* | *1.12* |
| Story Recall Delayed | 2.4 | **0.53** | *0.01* | *1.06* | 1.7 | **0.41** | *-0.14* | *0.96* |
| Verbal Fluency | 1.3 | **0.34** | *-0.18* | *0.86* | 1.4 | **0.27** | *-0.28* | *0.82* |
| DTMS Errors | -0.1 | **-0.06** | *-0.58* | *0.46* | -0.7 | **-0.38** | *-0.93* | *0.17* |
| DTMS L Sim | -132.0 | **-0.22** | *-0.74* | *0.29* | -239.0 | **-0.24** | *-0.79* | *0.31* |
| DTMS L 0 | -450.0 | **-0.57** | *-1.10* | *-0.05* | -141.0 | **-0.14** | *-0.69* | *0.40* |
| DTMS L 4 | -498.0 | **-0.52** | *-1.05* | *0.01* | -62.0 | **-0.06** | *-0.61* | *0.48* |
| DTMS L 12 | -353.0 | **-0.37** | *-0.89* | *0.16* | -749.0 | **-0.62** | *-1.18* | *-0.06* |
| PASAT 1.5 | 3.7 | **0.34** | *-0.18* | *0.86* | 3.3 | **0.32** | *-0.23* | *0.87* |
| PASAT 1.2 | 3.6 | **0.37** | *-0.15* | *0.90* | 3.4 | **0.34** | *-0.21* | *0.89* |

Table 12. Hartley et al., (2004) memory and attention measures: comparing effect sizes between Baseline and 12 weeks

|  | Placebo | | | | Gincosan | | | |
| --- | --- | --- | --- | --- | --- | --- | --- | --- |
|  | Mean | *d* | LCI | UCI | Mean | *d* | LCI | UCI |
| Picture Recall | 2.1 | **0.93** | *0.39* | *1.47* | 1.7 | **0.59** | *0.03* | *1.15* |
| Story Recall Immediate | 1.9 | **0.40** | *-0.12* | *0.92* | 1.5 | **0.38** | *-0.17* | *0.94* |
| Story Recall Delayed | 2.0 | **0.42** | *-0.10* | *0.95* | 1.0 | **0.24** | *-0.31* | *0.79* |
| Verbal Fluency | 2.5 | **0.57** | *0.04* | *1.09* | 3.2 | **0.58** | *0.02* | *1.14* |
| DTMS Errors | 0.0 | **0.00** | *-0.52* | *0.52* | -0.8 | **-0.44** | *-0.99* | *0.12* |
| DTMS L Sim | -383.0 | **-0.57** | *-1.10* | *-0.04* | -483.0 | **-0.48** | *-1.03* | *0.08* |
| DTMS L 0 | -438.0 | **-0.57** | *-1.10* | *-0.05* | -112.0 | **-0.11** | *-0.66* | *0.44* |
| DTMS L 4 | -413.0 | **-0.40** | *-0.92* | *0.13* | -270.0 | **-0.29** | *-0.84* | *0.25* |
| DTMS L 12 | -8.0 | **-0.01** | *-0.52* | *0.51* | -273.0 | **-0.22** | *-0.77* | *0.33* |
| PASAT 1.5 | 7.0 | **0.64** | *0.11* | *1.17* | 5.7 | **0.46** | *-0.10* | *1.01* |
| PASAT 1.2 | 4.6 | **0.43** | *-0.09* | *0.95* | 4.7 | **0.41** | *-0.14* | *0.96* |

Table 13. Hartley et al., (2004) memory and attention measures: comparing effect sizes between 6 weeks and 12 weeks

|  | Placebo | | | | Gincosan | | | |
| --- | --- | --- | --- | --- | --- | --- | --- | --- |
|  | Mean | *d* | LCI | UCI | Mean | *d* | LCI | UCI |
| Picture Recall | -0.3 | **-0.18** | *-0.70* | *0.34* | 0.5 | **0.16** | *-0.39* | *0.71* |
| Story Recall Immediate | -1.1 | **-0.31** | *-0.83* | *0.21* | -0.7 | **-0.19** | *-0.74* | *0.35* |
| Story Recall Delayed | -0.4 | **-0.11** | *-0.63* | *0.41* | -0.7 | **-0.17** | *-0.72* | *0.38* |
| Verbal Fluency | 1.2 | **0.27** | *-0.25* | *0.79* | 1.8 | **0.30** | *-0.25* | *0.85* |
| DTMS Errors | 0.1 | **0.07** | *-0.45* | *0.59* | -0.1 | **-0.05** | *-0.59* | *0.50* |
| DTMS L Sim | -251.0 | **-0.39** | *-0.91* | *0.13* | -244.0 | **-0.30** | *-0.85* | *0.25* |
| DTMS L 0 | 12.0 | **0.02** | *-0.50* | *0.54* | 29.0 | **0.03** | *-0.51* | *0.58* |
| DTMS L 4 | 85.0 | **0.09** | *-0.43* | *0.61* | -208.0 | **-0.23** | *-0.78* | *0.32* |
| DTMS L 12 | 345.0 | **0.33** | *-0.19* | *0.85* | 476.0 | **0.58** | *0.03* | *1.14* |
| PASAT 1.5 | 3.3 | **0.32** | *-0.20* | *0.84* | 2.4 | **0.17** | *-0.38* | *0.71* |
| PASAT 1.2 | 1.0 | **0.10** | *-0.42* | *0.62* | 1.3 | **0.12** | *-0.43* | *0.66* |

Table 14. Hartley et al., (2004) frontal lobe cognition measures: comparing effect sizes between Baseline and 6 weeks

|  | Placebo | | | | Gincosan | | | |
| --- | --- | --- | --- | --- | --- | --- | --- | --- |
|  | Mean | *d* | LCI | UCI | Mean | *d* | LCI | UCI |
| Soc4 moves | 0.1 | **0.09** | *-0.43* | *0.61* | 0.2 | **0.19** | *-0.35* | *0.74* |
| Soc4 iTime | -831.0 | **-0.18** | *-0.70* | *0.34* | 84.0 | **0.02** | *-0.53* | *0.56* |
| Soc4 sTime | -373.0 | **-0.22** | *-0.74* | *0.30* | -79.0 | **-0.04** | *-0.58* | *0.51* |
| Soc5 moves | -0.1 | **-0.06** | *-0.58* | *0.46* | -0.7 | **-0.45** | *-1.00* | *0.10* |
| Soc5 iTime | -887.0 | **-0.14** | *-0.66* | *0.38* | -213.0 | **-0.03** | *-0.57* | *0.52* |
| Soc5 sTime | -644.0 | **-0.39** | *-0.91* | *0.13* | -1007.0 | **-0.63** | *-1.19* | *-0.07* |
| IDED Trials | -1.3 | **-0.06** | *-0.58* | *0.46* | -5.3 | **-0.22** | *-0.77* | *0.32* |
| IDED Errors | 0.2 | **0.02** | *-0.50* | *0.53* | -3.7 | -0.28 | *-0.83* | *0.26* |

Table 15. Hartley et al., (2004) frontal lobe cognition measures: comparing effect sizes between Baseline and 12 weeks

|  | Placebo | | | | Gincosan | | | |
| --- | --- | --- | --- | --- | --- | --- | --- | --- |
|  | Mean | *d* | LCI | UCI | Mean | *d* | LCI | UCI |
| Soc4 moves | -0.2 | **-0.18** | *-0.70* | *0.34* | -0.5 | **-0.61** | *-1.17* | *-0.05* |
| Soc4 iTime | -1229.0 | **-0.29** | *-0.81* | *0.23* | -1608.0 | **-0.37** | *-0.92* | *0.18* |
| Soc4 sTime | -585.0 | **-0.35** | *-0.87* | *0.17* | -1287.0 | **-0.76** | *-1.33* | *-0.20* |
| Soc5 moves | 0.4 | **0.24** | *-0.28* | *0.76* | -0.5 | **-0.32** | *-0.87* | *0.23* |
| Soc5 iTime | -228.0 | **-0.04** | *-0.56* | *0.48* | -967.0 | **-0.13** | *-0.68* | *0.41* |
| Soc5 sTime | -230.0 | **-0.13** | *-0.65* | *0.39* | -954.0 | **-0.62** | *-1.18* | *-0.06* |
| IDED Trials | -8.1 | **-0.42** | *-0.94* | *0.11* | -10.9 | **-0.53** | *-1.09* | *0.03* |
| IDED Errors | -2.9 | **-0.25** | *-0.77* | *0.27* | -5.6 | **-0.46** | *-1.01* | *0.09* |

Table 16. Hartley et al., (2004) frontal lobe cognition measures: comparing effect sizes between 6 weeks and 12 weeks

|  | Placebo | | | | Gincosan | | | |
| --- | --- | --- | --- | --- | --- | --- | --- | --- |
|  | Mean | *d* | LCI | UCI | Mean | *d* | LCI | UCI |
| Soc4 moves | -0.3 | **-0.27** | *-0.79* | *0.25* | -0.7 | **-0.85** | *-1.42* | *-0.28* |
| Soc4 iTime | -398.0 | **-0.11** | *-0.63* | *0.41* | -1692.0 | **-0.34** | *-0.89* | *0.21* |
| Soc4 sTime | -212.0 | **-0.12** | *-0.64* | *0.40* | -1208.0 | **-0.67** | *-1.23* | *-0.11* |
| Soc5 moves | 0.5 | **0.30** | *-0.22* | *0.82* | 0.2 | **0.13** | *-0.42* | *0.68* |
| Soc5 iTime | 659.0 | **0.12** | *-0.39* | *0.64* | -754.0 | **-0.12** | *-0.67* | *0.42* |
| Soc5 sTime | 414.0 | **0.30** | *-0.22* | *0.82* | 53.0 | **0.05** | *-0.50* | *0.59* |
| IDED Trials | -6.8 | **-0.37** | *-0.89* | *0.15* | -5.6 | **-0.25** | *-0.80* | *0.30* |
| IDED Errors | -3.1 | **-0.26** | *-0.78* | *0.26* | -1.9 | **-0.15** | *-0.70* | *0.40* |

Table 17. Kennedy et al., (2001): comparing effect sizes between Placebo and 320mg

|  | Baseline | | | | 1 hour | | | | 2.5 hours | | | | 4 hours | | | | 6 hours | | | |
| --- | --- | --- | --- | --- | --- | --- | --- | --- | --- | --- | --- | --- | --- | --- | --- | --- | --- | --- | --- | --- |
|  | Mean | *d* | LCI | UCI | Mean | *d* | LCI | UCI | Mean | *d* | LCI | UCI | Mean | *d* | LCI | UCI | Mean | *d* | LCI | UCI |
| Quality of Memory | -14.23 | **-0.32** | *-0.97* | *0.32* | -0.80 | **-0.02** | *-0.66* | *0.62* | -9.25 | **-0.21** | *-0.85* | *0.43* | -13.33 | **-0.30** | *-0.95* | *0.34* | 5.32 | **0.12** | *-0.52* | *0.76* |
| Secondary Memory | -12.25 | **-0.33** | *-0.98* | *0.31* | -5.11 | **-0.14** | *-0.78* | *0.50* | -4.75 | **-0.13** | *-0.77* | *0.51* | -2.29 | **-0.06** | *-0.70* | *0.58* | 6.75 | **0.18** | *-0.46* | *0.82* |
| Working Memory | -1.97 | **-0.13** | *-0.77* | *0.51* | 4.32 | **0.28** | *-0.36* | *0.93* | 25.51 | **1.68** | *0.94* | *2.42* | -10.63 | **-0.70** | *-1.36* | *-0.04* | -1.42 | **-0.09** | *-0.73* | *0.55* |
| Speed of Memory | -25.45 | **-0.06** | *-0.70* | *0.58* | -42.27 | **-0.10** | *-0.74* | *0.54* | -31.49 | **-0.08** | *-0.72* | *0.56* | 69.29 | **0.17** | *-0.47* | *0.81* | -45.36 | **-0.11** | *-0.75* | *0.53* |
| Quality of Attention | 0.15 | **0.06** | *-0.58* | *0.70* | 0.15 | **0.06** | *-0.58* | *0.70* | -0.05 | **-0.02** | *-0.66* | *0.62* | 0.55 | **0.22** | *-0.42* | *0.86* | -0.50 | **-0.20** | *-0.84* | *0.44* |
| Speed of Attention | -37.10 | **-0.31** | *-0.96* | *0.33* | -16.26 | **-0.14** | *-0.78* | *0.50* | -9.25 | **-0.08** | *-0.72* | *0.56* | 0.34 | **0.00** | *-0.64* | *0.64* | 13.05 | **0.11** | *-0.53* | *0.75* |

Table 18. Kennedy et al., (2001): comparing effect sizes between Placebo and 640mg

|  | Baseline | | | | 1 hour | | | | 2.5 hours | | | | 4 hours | | | | 6 hours | | | |
| --- | --- | --- | --- | --- | --- | --- | --- | --- | --- | --- | --- | --- | --- | --- | --- | --- | --- | --- | --- | --- |
|  | Mean | *d* | LCI | UCI | Mean | *d* | LCI | UCI | Mean | *d* | LCI | UCI | Mean | *d* | LCI | UCI | Mean | *d* | LCI | UCI |
| Quality of Memory | -29.50 | **-0.51** | *-1.16* | *0.14* | -25.64 | **-0.44** | *-1.09* | *0.21* | -12.44 | **-0.21** | *-0.86* | *0.43* | -35.15 | **-0.60** | *-1.26* | *0.05* | -7.39 | **-0.13** | *-0.77* | *0.51* |
| Secondary Memory | -20.08 | **-0.41** | *-1.06* | *0.24* | -24.75 | **-0.51** | *-1.16* | *0.15* | -3.83 | **-0.08** | *-0.72* | *0.56* | -24.87 | **-0.51** | *-1.16* | *0.14* | -1.74 | **-0.04** | *-0.68* | *0.60* |
| Working Memory | -9.41 | **-0.57** | *-1.22* | *0.08* | -0.88 | **-0.05** | *-0.69* | *0.59* | 21.40 | **1.30** | *0.60* | *2.00* | -9.88 | **-0.60** | *-1.25* | *0.05* | -5.63 | **-0.34** | *-0.99* | *0.30* |
| Speed of Memory | -15.05 | **-0.04** | *-0.68* | *0.60* | 15.93 | **0.05** | *-0.59* | *0.69* | 61.57 | **0.18** | *-0.46* | *0.82* | 13.84 | **0.04** | *-0.60* | *0.68* | -13.76 | **-0.04** | *-0.68* | *0.60* |
| Quality of Attention | -0.15 | **-0.05** | *-0.69* | *0.59* | -0.20 | **-0.07** | *-0.71* | *0.57* | -1.40 | **-0.48** | *-1.13* | *0.17* | -1.10 | **-0.38** | *-1.02* | *0.27* | -1.15 | **-0.39** | *-1.04* | *0.25* |
| Speed of Attention | 3.64 | **0.03** | *-0.61* | *0.67* | -3.66 | **-0.03** | *-0.67* | *0.61* | -6.62 | **-0.05** | *-0.69* | *0.59* | 32.10 | **0.24** | *-0.40* | *0.88* | -4.05 | **-0.03** | *-0.67* | *0.61* |

Table 19. Kennedy et al., (2001): comparing effect sizes between Placebo and 960mg

|  | Baseline | | | | 1 hour | | | | 2.5 hours | | | | 4 hours | | | | 6 hours | | | |
| --- | --- | --- | --- | --- | --- | --- | --- | --- | --- | --- | --- | --- | --- | --- | --- | --- | --- | --- | --- | --- |
|  | Mean | *d* | LCI | UCI | Mean | *d* | LCI | UCI | Mean | *d* | LCI | UCI | Mean | *d* | LCI | UCI | Mean | *d* | LCI | UCI |
| Quality of Memory | -27.27 | **-0.53** | *-1.18* | *0.12* | 13.11 | **0.25** | *-0.39* | *0.90* | -12.93 | **-0.25** | *-0.89* | *0.39* | -6.81 | **-0.13** | *-0.77* | *0.51* | 9.99 | **0.19** | *-0.45* | *0.83* |
| Secondary Memory | -25.83 | **-0.52** | *-1.17* | *0.13* | 12.33 | **0.25** | *-0.39* | *0.89* | -12.33 | **-0.25** | *-0.89* | *0.39* | -3.46 | **-0.07** | *-0.71* | *0.57* | 19.09 | **0.39** | *-0.26* | *1.03* |
| Working Memory | -1.43 | **-0.11** | *-0.75* | *0.54* | 0.78 | **0.06** | *-0.58* | *0.70* | 29.41 | **2.17** | *1.36* | *2.98* | -2.95 | **-0.22** | *-0.86* | *0.42* | -9.08 | **-0.67** | *-1.33* | *-0.01* |
| Speed of Memory | -38.28 | **-0.11** | *-0.75* | *0.53* | 4.63 | **0.01** | *-0.63* | *0.65* | 16.32 | **0.05** | *-0.59* | *0.69* | 10.17 | **0.03** | *-0.61* | *0.67* | -28.65 | **-0.08** | *-0.72* | *0.56* |
| Quality of Attention | 0.40 | **0.16** | *-0.48* | *0.80* | -0.10 | **-0.04** | *-0.68* | *0.60* | -0.90 | **-0.35** | *-1.00* | *0.29* | 0.30 | **0.12** | *-0.52* | *0.76* | -0.05 | **-0.02** | *-0.66* | *0.62* |
| Speed of Attention | -18.99 | **-0.15** | *-0.79* | *0.49* | -25.77 | **-0.20** | *-0.84* | *0.44* | -29.43 | **-0.23** | -0.87 | 0.41 | 4.24 | **0.03** | *-0.61* | *0.67* | -16.78 | **-0.13** | *-0.77* | *0.51* |

Table 20. Kennedy et al., (2001): comparing effect sizes between 320mg and 640mg

|  | Baseline | | | | 1 hour | | | | 2.5 hours | | | | 4 hours | | | | 6 hours | | | |
| --- | --- | --- | --- | --- | --- | --- | --- | --- | --- | --- | --- | --- | --- | --- | --- | --- | --- | --- | --- | --- |
|  | Mean | *d* | LCI | UCI | Mean | *d* | LCI | UCI | Mean | *d* | LCI | UCI | Mean | *d* | LCI | UCI | Mean | *d* | LCI | UCI |
| Quality of Memory | -15.27 | **-0.26** | *-0.90* | *0.38* | -24.84 | **-0.43** | *-1.07* | *0.22* | -3.19 | **-0.05** | *-0.69* | *0.59* | -21.82 | **-0.37** | *-1.02* | *0.27* | -12.71 | **-0.22** | *-0.86* | *0.42* |
| Secondary Memory | -7.83 | **-0.17** | *-0.81* | *0.47* | -19.64 | **-0.43** | *-1.07* | *0.22* | 0.92 | **0.02** | *-0.62* | *0.66* | -22.58 | **-0.49** | *-1.14* | *0.16* | -8.49 | **-0.18** | *-0.83* | *0.46* |
| Working Memory | -7.44 | **-0.38** | *-1.03* | *0.26* | -5.20 | **-0.27** | *-0.91* | *0.37* | -4.11 | **-0.21** | *-0.85* | *0.43* | 0.75 | **0.04** | *-0.60* | *0.68* | -4.21 | **-0.22** | *-0.86* | *0.42* |
| Speed of Memory | 10.40 | **0.03** | *-0.61* | *0.67* | 58.20 | **0.16** | *-0.48* | *0.80* | 93.06 | **0.26** | *-0.38* | *0.90* | -55.45 | **-0.15** | *-0.80* | *0.49* | 31.60 | **0.09** | *-0.55* | *0.73* |
| Quality of Attention | -0.30 | **-0.11** | *-0.75* | *0.53* | -0.35 | **-0.12** | *-0.76* | *0.52* | -1.35 | **-0.48** | *-1.13* | *0.17* | -1.65 | **-0.58** | *-1.24* | *0.07* | -0.65 | **-0.23** | *-0.87* | *0.41* |
| Speed of Attention | 40.74 | **0.36** | *-0.29* | *1.00* | 12.60 | **0.11** | *-0.53* | *0.75* | 2.63 | **0.02** | *-0.62* | *0.66* | 31.76 | **0.28** | *-0.36* | *0.92* | -17.10 | **-0.15** | *-0.79* | *0.49* |

Table 21. Kennedy et al., (2001): comparing effect sizes between 320mg and 960mg

|  | Baseline | | | | 1 hour | | | | 2.5 hours | | | | 4 hours | | | | 6 hours | | | |
| --- | --- | --- | --- | --- | --- | --- | --- | --- | --- | --- | --- | --- | --- | --- | --- | --- | --- | --- | --- | --- |
|  | Mean | *d* | LCI | UCI | Mean | *d* | LCI | UCI | Mean | *d* | LCI | UCI | Mean | *d* | LCI | UCI | Mean | *d* | LCI | UCI |
| Quality of Memory | -13.04 | **-0.25** | *-0.89* | *0.39* | 13.91 | **0.27** | *-0.37* | *0.91* | -3.68 | **-0.07** | *-0.71* | *0.57* | 6.52 | **0.13** | *-0.51* | *0.77* | 4.67 | **0.09** | *-0.55* | *0.73* |
| Secondary Memory | -13.58 | **-0.29** | *-0.94* | *0.35* | 17.44 | **0.37** | *-0.27* | *1.02* | -7.58 | **-0.16** | *-0.80* | *0.48* | -1.17 | **-0.03** | *-0.67* | *0.62* | 12.34 | **0.26** | *-0.38* | *0.91* |
| Working Memory | 0.54 | **0.03** | *-0.61* | *0.67* | -3.54 | **-0.21** | *-0.85* | *0.43* | 3.90 | **0.23** | *-0.41* | *0.87* | 7.68 | **0.45** | *-0.19* | *1.10* | -7.66 | **-0.45** | *-1.10* | *0.20* |
| Speed of Memory | -12.83 | **-0.03** | *-0.67* | *0.61* | 46.90 | **0.13** | *-0.51* | *0.77* | 47.81 | **0.13** | *-0.51* | *0.77* | -59.12 | **-0.16** | *-0.80* | *0.48* | 16.71 | **0.04** | *-0.60* | *0.69* |
| Quality of Attention | 0.25 | **0.10** | *-0.54* | *0.74* | -0.25 | **-0.10** | *-0.74* | *0.54* | -0.85 | **-0.35** | *-0.99* | *0.30* | -0.25 | **-0.10** | *-0.74* | *0.54* | 0.45 | **0.18** | *-0.46* | *0.83* |
| Speed of Attention | 18.11 | **0.17** | *-0.47* | *0.81* | -9.51 | **-0.09** | *-0.73* | *0.55* | -20.18 | **-0.19** | *-0.83* | *0.46* | 3.90 | **0.04** | *-0.60* | *0.68* | -29.83 | **-0.28** | *-0.92* | *0.37* |

Table 22. Kennedy et al., (2001): comparing effect sizes between 640mg and 960mg

|  | Baseline | | | | 1 hour | | | | 2.5 hours | | | | 4 hours | | | | 6 hours | | | |
| --- | --- | --- | --- | --- | --- | --- | --- | --- | --- | --- | --- | --- | --- | --- | --- | --- | --- | --- | --- | --- |
|  | Mean | *d* | LCI | UCI | Mean | *d* | LCI | UCI | Mean | *d* | LCI | UCI | Mean | *d* | LCI | UCI | Mean | *d* | LCI | UCI |
| Quality of Memory | 2.23 | **0.03** | *-0.61* | *0.67* | 38.75 | **0.60** | *-0.05* | *1.26* | -0.49 | **-0.01** | *-0.65* | *0.63* | 28.34 | **0.44** | *-0.21* | *1.09* | 17.38 | **0.27** | *-0.37* | *0.91* |
| Secondary Memory | -5.75 | **-0.10** | *-0.74* | *0.54* | 37.08 | **0.65** | *0.00* | *1.31* | -8.50 | **-0.15** | *-0.79* | *0.49* | 21.41 | **0.38** | *-0.27* | *1.02* | 20.83 | **0.37** | *-0.28* | *1.01* |
| Working Memory | 7.98 | **0.44** | *-0.21* | *1.09* | 1.66 | **0.09** | *-0.55* | *0.73* | 8.01 | **0.44** | *-0.20* | *1.09* | 6.93 | **0.38** | *-0.26* | *1.03* | -3.45 | **-0.19** | *-0.83* | *0.45* |
| Speed of Memory | -23.23 | **-0.08** | *-0.72* | *0.56* | -11.30 | **-0.04** | *-0.68* | *0.60* | -45.25 | **-0.15** | *-0.79* | *0.49* | -3.67 | **-0.01** | *-0.65* | *0.63* | -14.89 | **-0.05** | *-0.69* | *0.59* |
| Quality of Attention | 0.55 | **0.19** | *-0.45* | *0.84* | 0.10 | **0.04** | *-0.61* | *0.68* | 0.50 | **0.18** | *-0.47* | *0.82* | 1.40 | **0.49** | *-0.16* | *1.14* | 1.10 | **0.39** | *-0.26* | *1.03* |
| Speed of Attention | -22.63 | **-0.18** | *-0.82* | *0.46* | -22.11 | **-0.18** | *-0.82* | *0.46* | -22.81 | **-0.18** | *-0.83* | *0.46* | -27.86 | **-0.22** | *-0.87* | *0.42* | -12.73 | -0.10 | *-0.74* | *0.54* |

Table 23. Kennedy et al., (2001): comparing effect sizes between Baseline and 1 hour

|  | Placebo | | | | 320mg | | | | 640mg | | | | 960mg | | | |
| --- | --- | --- | --- | --- | --- | --- | --- | --- | --- | --- | --- | --- | --- | --- | --- | --- |
|  | Mean | *d* | LCI | UCI | Mean | *d* | LCI | UCI | Mean | *d* | LCI | UCI | Mean | *d* | LCI | UCI |
| Quality of Memory | -41.53 | **-0.95** | *-1.62* | *-0.27* | -28.10 | **-0.64** | *-1.30* | *0.02* | -37.67 | **-0.54** | *-1.19* | *0.11* | -1.15 | **-0.02** | *-0.66* | *0.62* |
| Secondary Memory | -35.33 | **-0.88** | *-1.55* | *-0.21* | -28.19 | **-0.85** | *-1.52* | *-0.18* | -40.00 | **-0.71** | *-1.37* | *-0.05* | 2.83 | **0.05** | *-0.59* | *0.69* |
| Working Memory | -6.20 | **-0.55** | *-1.20* | *0.10* | 0.09 | **0.00** | *-0.64* | *0.65* | 2.33 | **0.11** | *-0.53* | *0.76* | -3.99 | **-0.26** | *-0.90* | *0.38* |
| Speed of Memory | -28.27 | **-0.07** | *-0.71* | *0.57* | -45.09 | **-0.11** | *-0.75* | *0.53* | 2.71 | **0.01** | *-0.63* | *0.65* | 14.64 | **0.05** | *-0.59* | *0.69* |
| Quality of Attention | 0.35 | **0.13** | *-0.51* | *0.77* | 0.35 | **0.14** | *-0.50* | *0.79* | 0.30 | **0.09** | *-0.55* | *0.74* | -0.15 | **-0.06** | *-0.70* | *0.58* |
| Speed of Attention | 11.41 | **0.08** | *-0.56* | *0.72* | 32.25 | **0.34** | *-0.31* | *0.98* | 4.11 | **0.03** | *-0.61* | *0.67* | 4.63 | **0.04** | *-0.60* | *0.68* |

Table 24. Kennedy et al., (2001): comparing effect sizes between Baseline and 2.5 hours

|  | Placebo | | | | 320mg | | | | 640mg | | | | 960mg | | | |
| --- | --- | --- | --- | --- | --- | --- | --- | --- | --- | --- | --- | --- | --- | --- | --- | --- |
|  | Mean | *d* | LCI | UCI | Mean | *d* | LCI | UCI | Mean | *d* | LCI | UCI | Mean | *d* | LCI | UCI |
| Quality of Memory | -47.69 | **-1.09** | *-1.77* | *-0.40* | -42.71 | **-0.97** | *-1.65* | *-0.29* | -30.63 | **-0.44** | *-1.09* | *0.21* | -33.35 | **-0.57** | *-1.22* | *0.08* |
| Secondary Memory | -43.92 | **-1.09** | *-1.77* | *-0.40* | -36.42 | **-1.10** | *-1.79* | *-0.41* | -27.67 | **-0.49** | *-1.14* | *0.16* | -30.42 | **-0.53** | *-1.19* | *0.12* |
| Working Memory | -33.78 | **-2.99** | *-3.92* | *-2.05* | -6.30 | **-0.35** | *-0.99* | *0.30* | -2.97 | **-0.15** | *-0.79* | *0.50* | -2.94 | **-0.19** | *-0.83* | *0.45* |
| Speed of Memory | -94.49 | **-0.24** | *-0.89* | *0.40* | -100.53 | **-0.24** | *-0.88* | *0.41* | -17.87 | **-0.06** | *-0.70* | *0.58* | -39.89 | **-0.13** | *-0.77* | *0.51* |
| Quality of Attention | -0.05 | **-0.02** | *-0.66* | *0.62* | -0.25 | **-0.10** | *-0.74* | *0.54* | -1.30 | **-0.41** | *-1.06* | *0.24* | -1.35 | **-0.55** | *-1.20* | *0.10* |
| Speed of Attention | -0.39 | **0.00** | *-0.64* | *0.64* | 27.46 | **0.29** | *-0.36* | *0.93* | -10.65 | **-0.08** | *-0.72* | *0.56* | -10.83 | **-0.09** | *-0.73* | *0.55* |

Table 25. Kennedy et al., (2001): comparing effect sizes between Baseline and 4 hours

|  | Placebo | | | | 320mg | | | | 640mg | | | | 960mg | | | |
| --- | --- | --- | --- | --- | --- | --- | --- | --- | --- | --- | --- | --- | --- | --- | --- | --- |
|  | Mean | *d* | LCI | UCI | Mean | *d* | LCI | UCI | Mean | *d* | LCI | UCI | Mean | *d* | LCI | UCI |
| Quality of Memory | -43.61 | **-1.00** | *-1.67* | *-0.32* | -42.71 | **-0.97** | *-1.65* | *-0.29* | -49.26 | **-0.70** | *-1.36* | *-0.04* | -23.15 | **-0.40** | *-1.04* | *0.25* |
| Secondary Memory | -39.54 | **-0.98** | *-1.66* | *-0.30* | -29.58 | **-0.90** | *-1.57* | *-0.22* | -44.33 | **-0.79** | *-1.45* | *-0.12* | -17.17 | **-0.30** | *-0.95* | *0.34* |
| Working Memory | -4.46 | **-0.39** | *-1.04* | *0.25* | -13.12 | **-0.72** | *-1.38* | *-0.06* | -4.93 | **-0.24** | *-0.88* | *0.40* | -5.98 | **-0.39** | *-1.03* | *0.26* |
| Speed of Memory | -107.62 | **-0.28** | *-0.92* | *0.37* | -12.88 | **-0.03** | *-0.67* | *0.61* | -78.73 | **-0.28** | *-0.93* | *0.36* | -59.17 | **-0.19** | *-0.83* | *0.45* |
| Quality of Attention | -0.60 | **-0.23** | *-0.87* | *0.41* | -0.20 | **-0.08** | *-0.72* | *0.56* | -1.55 | **-0.49** | *-1.14* | *0.16* | -0.70 | **-0.28** | *-0.93* | *0.36* |
| Speed of Attention | -3.60 | **-0.03** | *-0.67* | *0.61* | 33.84 | **0.35** | *-0.29* | *1.00* | 24.86 | **0.19** | *-0.45* | *0.83* | 19.63 | **0.16** | *-0.48* | *0.81* |

Table 26. Kennedy et al., (2001): comparing effect sizes between Baseline and 6 hours

|  | Placebo | | | | 320mg | | | | 640mg | | | | 960mg | | | |
| --- | --- | --- | --- | --- | --- | --- | --- | --- | --- | --- | --- | --- | --- | --- | --- | --- |
|  | Mean | *d* | LCI | UCI | Mean | *d* | LCI | UCI | Mean | *d* | LCI | UCI | Mean | *d* | LCI | UCI |
| Quality of Memory | -69.90 | **-1.59** | *-2.33* | *-0.86* | -50.35 | **-1.15** | *-1.84* | *-0.45* | -47.79 | **-0.68** | *-1.34* | *-0.02* | -32.64 | **-0.56** | *-1.21* | *0.09* |
| Secondary Memory | -59.92 | **-1.49** | *-2.21* | *-0.76* | -40.92 | **-1.24** | *-1.94* | *-0.54* | -41.58 | **-0.74** | *-1.40* | *-0.08* | -15.00 | **-0.26** | *-0.91* | *0.38* |
| Working Memory | -9.99 | **-0.88** | *-1.55* | *-0.21* | -9.44 | **-0.52** | *-1.17* | *0.13* | -6.21 | **-0.31** | *-0.95* | *0.34* | -17.64 | **-1.14** | *-1.83* | *-0.45* |
| Speed of Memory | -83.07 | **-0.21** | *-0.86* | *0.43* | -102.98 | **-0.24** | *-0.89* | *0.40* | -81.78 | **-0.29** | *-0.94* | *0.35* | -73.44 | **-0.24** | *-0.88* | *0.41* |
| Quality of Attention | -1.00 | **-0.38** | *-1.02* | *0.27* | -1.65 | **-0.68** | *-1.34* | *-0.02* | -2.00 | **-0.63** | *-1.29* | *0.03* | -1.45 | **-0.59** | *-1.24* | *0.06* |
| Speed of Attention | 8.01 | **0.06** | *-0.58* | *0.70* | 58.16 | **0.61** | *-0.05* | *1.26* | 0.32 | **0.00** | *-0.64* | *0.64* | 10.22 | **0.09** | *-0.55* | *0.73* |

Table 27. Kennedy et al., (2001): comparing effect sizes between 1 hour and 2.5 hours

|  | Placebo | | | | 320mg | | | | 640mg | | | | 960mg | | | |
| --- | --- | --- | --- | --- | --- | --- | --- | --- | --- | --- | --- | --- | --- | --- | --- | --- |
|  | Mean | *d* | LCI | UCI | Mean | *d* | LCI | UCI | Mean | *d* | LCI | UCI | Mean | *d* | LCI | UCI |
| Quality of Memory | -6.16 | **-0.14** | *-0.78* | *0.50* | -14.61 | **-0.33** | *-0.98* | *0.31* | 7.04 | **0.10** | *-0.54* | *0.74* | -32.20 | **-0.55** | *-1.20* | *0.10* |
| Secondary Memory | -8.59 | **-0.21** | *-0.85* | *0.43* | -8.23 | **-0.25** | *-0.89* | *0.39* | 12.33 | **0.22** | *-0.42* | *0.86* | -33.25 | **-0.58** | *-1.24* | *0.07* |
| Working Memory | -27.58 | **-2.44** | *-3.28* | *-1.59* | -6.39 | **-0.35** | *-1.00* | *0.29* | -5.30 | **-0.26** | *-0.90* | *0.38* | 1.05 | **0.07** | *-0.57* | *0.71* |
| Speed of Memory | -66.22 | **-0.17** | *-0.81* | *0.47* | -55.44 | **-0.13** | *-0.77* | *0.51* | -20.58 | **-0.07** | *-0.71* | *0.57* | -54.53 | **-0.17** | *-0.82* | *0.47* |
| Quality of Attention | -0.40 | **-0.15** | *-0.79* | *0.49* | -0.60 | **-0.25** | *-0.89* | *0.39* | -1.60 | **-0.50** | *-1.15* | *0.15* | -1.20 | **-0.49** | *-1.14* | *0.16* |
| Speed of Attention | -11.80 | **-0.09** | *-0.73* | *0.56* | -4.79 | **-0.05** | *-0.69* | *0.59* | -14.76 | **-0.11** | *-0.76* | *0.53* | -15.46 | **-0.13** | *-0.77* | *0.51* |

Table 28. Kennedy et al., (2001): comparing effect sizes between 1 hour and 4 hours

|  | Placebo | | | | 320mg | | | | 640mg | | | | 960mg | | | |
| --- | --- | --- | --- | --- | --- | --- | --- | --- | --- | --- | --- | --- | --- | --- | --- | --- |
|  | Mean | *d* | LCI | UCI | Mean | *d* | LCI | UCI | Mean | *d* | LCI | UCI | Mean | *d* | LCI | UCI |
| Quality of Memory | -2.08 | **-0.05** | *-0.69* | *0.59* | -14.61 | **-0.33** | *-0.98* | *0.31* | -11.59 | **-0.17** | *-0.81* | *0.48* | -22.00 | **-0.38** | *-1.02* | *0.27* |
| Secondary Memory | -4.21 | **-0.10** | *-0.74* | *0.54* | -1.39 | **-0.04** | *-0.68* | *0.60* | -4.33 | **-0.08** | *-0.72* | *0.56* | -20.00 | **-0.35** | *-1.00* | *0.29* |
| Working Memory | 1.74 | **0.15** | *-0.49* | *0.79* | -13.21 | **-0.72** | *-1.38* | *-0.06* | -7.26 | **-0.36** | *-1.00* | *0.29* | -1.99 | **-0.13** | *-0.77* | *0.51* |
| Speed of Memory | -79.35 | **-0.20** | *-0.85* | *0.44* | 32.21 | **0.08** | *-0.56* | *0.72* | -81.44 | **-0.29** | *-0.94* | *0.35* | -73.81 | **-0.24** | *-0.88* | *0.41* |
| Quality of Attention | -0.95 | **-0.36** | *-1.01* | *0.29* | -0.55 | **-0.23** | *-0.87* | *0.41* | -1.85 | **-0.58** | *-1.24* | *0.07* | -0.55 | **-0.22** | *-0.87* | *0.42* |
| Speed of Attention | -15.01 | **-0.11** | *-0.75* | *0.53* | 1.59 | **0.02** | *-0.62* | *0.66* | 20.75 | **0.16** | *-0.48* | *0.80* | 15.00 | **0.13** | *-0.52* | *0.77* |

Table 29. Kennedy et al., (2001): comparing effect sizes between 1 hour and 6 hours

|  | Placebo | | | | 320mg | | | | 640mg | | | | 960mg | | | |
| --- | --- | --- | --- | --- | --- | --- | --- | --- | --- | --- | --- | --- | --- | --- | --- | --- |
|  | Mean | *d* | LCI | UCI | Mean | *d* | LCI | UCI | Mean | *d* | LCI | UCI | Mean | *d* | LCI | UCI |
| Quality of Memory | -28.37 | **-0.65** | *-1.30* | *0.01* | -22.25 | **-0.51** | *-1.16* | *0.14* | -10.12 | **-0.14** | *-0.79* | *0.50* | -31.49 | **-0.54** | *-1.19* | *0.11* |
| Secondary Memory | -24.59 | **-0.61** | *-1.26* | *0.05* | -12.73 | **-0.39** | *-1.03* | *0.26* | -1.58 | **-0.03** | *-0.67* | *0.61* | -17.83 | **-0.31** | *-0.96* | *0.33* |
| Working Memory | -3.79 | **-0.33** | *-0.98* | *0.31* | -9.53 | **-0.52** | *-1.17* | *0.13* | -8.54 | **-0.42** | *-1.07* | *0.23* | -13.65 | **-0.88** | *-1.55* | *-0.21* |
| Speed of Memory | -54.80 | **-0.14** | *-0.78* | *0.50* | -57.89 | **-0.14** | *-0.78* | *0.50* | -84.49 | **-0.30** | *-0.95* | *0.34* | -88.08 | **-0.28** | *-0.93* | *0.36* |
| Quality of Attention | -1.35 | **-0.51** | *-1.16* | *0.14* | -2.00 | **-0.83** | *-1.50* | *-0.16* | -2.30 | **-0.72** | *-1.39* | *-0.06* | -1.30 | **-0.53** | *-1.18* | *0.12* |
| Speed of Attention | -3.40 | **-0.02** | *-0.66* | *0.62* | 25.91 | **0.27** | *-0.37* | *0.91* | -3.79 | **-0.03** | *-0.67* | *0.61* | 5.59 | **0.05** | *-0.59* | *0.69* |

Table 30. Kennedy et al., (2001): comparing effect sizes between 2.5 hours and 4 hours

|  | Placebo | | | | 320mg | | | | 640mg | | | | 960mg | | | |
| --- | --- | --- | --- | --- | --- | --- | --- | --- | --- | --- | --- | --- | --- | --- | --- | --- |
|  | Mean | *d* | LCI | UCI | Mean | *d* | LCI | UCI | Mean | *d* | LCI | UCI | Mean | *d* | LCI | UCI |
| Quality of Memory | 4.08 | **0.09** | *-0.55* | *0.73* | 0.00 | **0.00** | *-0.64* | *0.64* | -18.63 | **-0.27** | *-0.91* | *0.38* | 10.20 | **0.17** | *-0.47* | *0.82* |
| Secondary Memory | 4.38 | **0.11** | *-0.53* | *0.75* | 6.84 | **0.21** | *-0.43* | *0.85* | -16.66 | **-0.30** | *-0.94* | *0.35* | 13.25 | **0.23** | *-0.41* | *0.87* |
| Working Memory | 29.32 | **2.59** | *1.72* | *3.46* | -6.82 | **-0.37** | *-1.02* | *0.27* | -1.96 | **-0.10** | *-0.74* | *0.54* | -3.04 | **-0.20** | *-0.84* | *0.45* |
| Speed of Memory | -13.13 | **-0.03** | *-0.67* | *0.61* | 87.65 | **0.21** | *-0.44* | *0.85* | -60.86 | **-0.22** | *-0.86* | *0.42* | -19.28 | **-0.06** | *-0.70* | *0.58* |
| Quality of Attention | -0.55 | **-0.21** | *-0.85* | *0.43* | 0.05 | **0.02** | *-0.62* | *0.66* | -0.25 | **-0.08** | *-0.72* | *0.56* | 0.65 | **0.26** | *-0.38* | *0.91* |
| Speed of Attention | -3.21 | **-0.02** | *-0.66* | *0.62* | 6.38 | **0.07** | *-0.57* | *0.71* | 35.51 | **0.28** | *-0.37* | *0.92* | 30.46 | **0.25** | *-0.39* | *0.90* |

Table 31. Kennedy et al., (2001): comparing effect sizes between 2.5 hours and 6 hours

|  | Placebo | | | | 320mg | | | | 640mg | | | | 960mg | | | |
| --- | --- | --- | --- | --- | --- | --- | --- | --- | --- | --- | --- | --- | --- | --- | --- | --- |
|  | Mean | *d* | LCI | UCI | Mean | *d* | LCI | UCI | Mean | *d* | LCI | UCI | Mean | *d* | LCI | UCI |
| Quality of Memory | -22.21 | **-0.51** | *-1.16* | *0.14* | -7.64 | **-0.17** | *-0.82* | *0.47* | -17.16 | **-0.25** | *-0.89* | *0.40* | 0.71 | **0.01** | *-0.63* | *0.65* |
| Secondary Memory | -16.00 | **-0.40** | *-1.04* | *0.25* | -4.50 | **-0.14** | *-0.78* | *0.50* | -13.91 | **-0.25** | *-0.89* | *0.40* | 15.42 | **0.27** | *-0.37* | *0.91* |
| Working Memory | 23.79 | **2.10** | *1.30* | *2.90* | -3.14 | **-0.17** | *-0.81* | *0.47* | -3.24 | **-0.16** | *-0.80* | *0.48* | -14.70 | **-0.95** | *-1.63* | *-0.27* |
| Speed of Memory | 11.42 | **0.03** | *-0.61* | *0.67* | -2.45 | **-0.01** | *-0.65* | *0.63* | -63.91 | **-0.23** | *-0.87* | *0.41* | -33.55 | **-0.11** | *-0.75* | *0.53* |
| Quality of Attention | -0.95 | **-0.36** | *-1.01* | *0.29* | -1.40 | **-0.58** | *-1.23* | *0.07* | -0.70 | **-0.22** | *-0.86* | *0.42* | -0.10 | **-0.04** | *-0.68* | *0.60* |
| Speed of Attention | 8.40 | **0.06** | *-0.58* | *0.70* | 30.70 | **0.32** | *-0.32* | *0.96* | 10.97 | **0.09** | *-0.56* | *0.73* | 21.05 | **0.18** | *-0.47* | *0.82* |

Table 32. Kennedy et al., (2001): comparing effect sizes between 4 hours and 6 hours

|  | Placebo | | | | 320mg | | | | 640mg | | | | 960mg | | | |
| --- | --- | --- | --- | --- | --- | --- | --- | --- | --- | --- | --- | --- | --- | --- | --- | --- |
|  | Mean | *d* | LCI | UCI | Mean | *d* | LCI | UCI | Mean | *d* | LCI | UCI | Mean | *d* | LCI | UCI |
| Quality of Memory | -26.29 | **-0.60** | *-1.25* | *0.05* | -7.64 | **-0.17** | *-0.82* | *0.47* | 1.47 | **0.02** | *-0.62* | *0.66* | -9.49 | **-0.16** | *-0.80* | *0.48* |
| Secondary Memory | -20.38 | **-0.51** | *-1.16* | *0.15* | -11.34 | **-0.34** | *-0.99* | *0.30* | 2.75 | **0.05** | *-0.59* | *0.69* | 2.17 | **0.04** | *-0.60* | *0.68* |
| Working Memory | -5.53 | **-0.49** | *-1.14* | *0.16* | 3.68 | **0.20** | *-0.44* | *0.84* | -1.28 | **-0.06** | *-0.70* | *0.58* | -11.66 | **-0.75** | *-1.42* | *-0.09* |
| Speed of Memory | 24.55 | **0.06** | *-0.58* | *0.70* | -90.10 | **-0.21** | *-0.85* | *0.43* | -3.05 | **-0.01** | *-0.65* | *0.63* | -14.27 | **-0.05** | *-0.69* | *0.59* |
| Quality of Attention | -0.40 | **-0.15** | *-0.79* | *0.49* | -1.45 | **-0.60** | *-1.25* | *0.05* | -0.45 | **-0.14** | *-0.78* | *0.50* | -0.75 | **-0.30** | *-0.95* | *0.34* |
| Speed of Attention | 11.61 | **0.08** | *-0.56* | *0.72* | 24.32 | **0.25** | *-0.39* | *0.90* | -24.54 | **-0.19** | *-0.83* | *0.45* | -9.41 | **-0.08** | *-0.72* | *0.56* |

Table 33. Kennedy et al., (2002): comparing effect sizes between Placebo and Ginkgo

|  | Baseline | | | | 1 hour | | | | 2.5 hours | | | | 4 hours | | | | 6 hours | | | |
| --- | --- | --- | --- | --- | --- | --- | --- | --- | --- | --- | --- | --- | --- | --- | --- | --- | --- | --- | --- | --- |
|  | Mean | *d* | LCI | UCI | Mean | *d* | LCI | UCI | Mean | *d* | LCI | UCI | Mean | *d* | LCI | UCI | Mean | *d* | LCI | UCI |
| Quality of Memory | -10.70 | **-0.15** | *-0.79* | *0.49* | 7.88 | **0.11** | *-0.53* | *0.75* | 10.50 | **0.15** | *-0.49* | *0.79* | 3.20 | **0.05** | *-0.60* | *0.69* | 18.67 | **0.26** | *-0.38* | *0.91* |
| Secondary Memory | -12.84 | **-0.22** | *-0.86* | *0.43* | 10.49 | **0.18** | *-0.47* | *0.82* | 6.72 | **0.11** | *-0.53* | *0.75* | 2.49 | **0.04** | *-0.60* | *0.68* | 14.83 | **0.25** | *-0.39* | *0.89* |
| Working Memory | 2.13 | **0.09** | *-0.55* | *0.73* | -2.63 | **-0.11** | *-0.76* | *0.53* | 3.77 | **0.16** | *-0.48* | *0.81* | 0.69 | **0.03** | *-0.61* | *0.67* | 3.83 | **0.17** | *-0.47* | *0.81* |
| Speed of Memory | -2.73 | **-0.01** | *-0.65* | *0.63* | 27.49 | **0.07** | *-0.57* | *0.71* | -15.50 | **-0.04** | *-0.68* | *0.60* | 52.32 | **0.13** | *-0.51* | *0.77* | 49.35 | **0.13** | *-0.51* | *0.77* |
| Quality of Attention | -0.45 | **-0.12** | *-0.76* | *0.52* | 0.80 | **0.21** | *-0.43* | *0.86* | 1.20 | **0.32** | *-0.32* | *0.96* | -0.60 | **-0.16** | *-0.80* | *0.48* | -1.10 | **-0.29** | *-0.94* | *0.35* |
| Speed of Attention | 5.98 | **0.05** | *-0.60* | *0.69* | -8.19 | **-0.06** | *-0.70* | *0.58* | 13.96 | **0.11** | *-0.54* | *0.75* | 25.62 | **0.19** | *-0.45* | *0.83* | -16.77 | **-0.13** | *-0.77* | *0.51* |
| Alert | -4.90 | **-0.28** | *-0.92* | *0.36* | 0.74 | **0.04** | *-0.60* | *0.68* | 0.79 | **0.05** | *-0.59* | *0.69* | 2.25 | **0.13** | *-0.51* | *0.77* | 3.46 | **0.20** | *-0.44* | *0.84* |
| Content | -7.59 | **-0.49** | *-1.14* | *0.16* | -1.12 | **-0.07** | *-0.71* | *0.57* | -3.20 | **-0.21** | *-0.85* | *0.43* | 0.44 | **0.03** | *-0.61* | *0.67* | 0.08 | **0.01** | *-0.63* | *0.65* |
| Calm | 2.40 | **0.16** | *-0.48* | *0.80* | 0.77 | **0.05** | *-0.59* | *0.69* | -0.02 | **0.00** | *-0.64* | *0.64* | 2.02 | **0.13** | *-0.51* | *0.77* | -6.96 | **-0.46** | *-1.11* | *0.19* |
| Serial3Responses | -1.17 | **-0.04** | *-0.68* | *0.60* | -2.28 | **-0.08** | *-0.72* | *0.56* | -1.00 | **-0.04** | *-0.68* | *0.60* | 1.89 | **0.07** | *-0.57* | *0.71* | 2.89 | **0.10** | *-0.54* | *0.74* |
| Serial3Errors | 1.00 | **0.26** | *-0.38* | *0.91* | 1.50 | **0.40** | *-0.25* | *1.04* | 0.29 | **0.08** | *-0.56* | *0.72* | -1.66 | **-0.44** | *-1.09* | *0.21* | -1.22 | **-0.32** | *-0.97* | *0.32* |
| Serial7Responses | -1.14 | **-0.06** | *-0.70* | *0.58* | 1.23 | **0.06** | *-0.58* | *0.70* | 0.07 | **0.00** | *-0.64* | *0.64* | 2.23 | **0.11** | *-0.53* | *0.75* | 3.70 | **0.19** | *-0.46* | *0.83* |
| Serial7Errors | 0.09 | **0.03** | *-0.61* | *0.67* | 0.72 | **0.24** | *-0.41* | *0.88* | 0.45 | **0.15** | *-0.49* | *0.79* | -1.17 | **-0.38** | *-1.03* | *0.26* | 0.40 | 0.13 | *-0.51* | *0.77* |

Table 34. Kennedy et al., (2002): comparing effect sizes between Placebo and Ginseng

|  | Baseline | | | | 1 hour | | | | 2.5 hours | | | | 4 hours | | | | 6 hours | | | |
| --- | --- | --- | --- | --- | --- | --- | --- | --- | --- | --- | --- | --- | --- | --- | --- | --- | --- | --- | --- | --- |
|  | Mean | *d* | LCI | UCI | Mean | *d* | LCI | UCI | Mean | *d* | LCI | UCI | Mean | *d* | LCI | UCI | Mean | *d* | LCI | UCI |
| Quality of Memory | -4.29 | **-0.06** | *-0.70* | *0.58* | -6.93 | **-0.10** | *-0.74* | *0.54* | 10.86 | **0.16** | *-0.48* | *0.80* | 22.65 | **0.33** | *-0.31* | *0.98* | 14.12 | **0.21** | *-0.43* | *0.85* |
| Secondary Memory | -6.34 | **-0.11** | *-0.76* | *0.53* | 0.91 | **0.02** | *-0.62* | *0.66* | 11.62 | **0.21** | *-0.43* | *0.85* | 26.24 | **0.48** | *-0.17* | *1.12* | 19.08 | **0.35** | *-0.30* | *0.99* |
| Working Memory | 2.04 | **0.10** | *-0.54* | *0.74* | -7.86 | **-0.40** | *-1.04* | *0.25* | -0.77 | **-0.04** | *-0.68* | *0.60* | -3.61 | **-0.18** | *-0.82* | *0.46* | -4.97 | **-0.25** | *-0.89* | *0.39* |
| Speed of Memory | 42.15 | **0.11** | *-0.53* | *0.75* | 13.93 | **0.04** | *-0.61* | *0.68* | -38.38 | **-0.10** | *-0.74* | *0.54* | -51.38 | **-0.13** | *-0.77* | *0.51* | 33.28 | **0.08** | *-0.56* | *0.72* |
| Quality of Attention | -2.10 | **-0.53** | *-1.18* | *0.12* | -0.80 | **-0.20** | *-0.84* | *0.44* | 0.45 | **0.11** | *-0.53* | *0.75* | -0.50 | **-0.13** | *-0.77* | *0.51* | -1.20 | **-0.30** | *-0.95* | *0.34* |
| Speed of Attention | 4.06 | **0.03** | *-0.61* | *0.67* | -6.98 | **-0.05** | *-0.69* | *0.59* | 1.13 | **0.01** | *-0.63* | *0.65* | 21.43 | **0.17** | *-0.47* | *0.81* | -13.52 | **-0.11** | *-0.75* | *0.53* |
| Alert | -3.09 | **-0.18** | *-0.82* | *0.47* | -0.90 | **-0.05** | *-0.69* | *0.59* | 0.20 | **0.01** | *-0.63* | *0.65* | 1.38 | **0.08** | *-0.56* | *0.72* | -1.51 | **-0.09** | *-0.73* | *0.55* |
| Content | -4.76 | **-0.34** | *-0.99* | *0.30* | -2.62 | **-0.19** | *-0.83* | *0.45* | -0.76 | **-0.05** | *-0.70* | *0.59* | -1.70 | **-0.12** | *-0.76* | *0.52* | -1.68 | **-0.12** | *-0.76* | *0.52* |
| Calm | 0.45 | **0.02** | *-0.62* | *0.67* | -3.11 | **-0.17** | *-0.81* | *0.47* | -1.97 | **-0.11** | *-0.75* | *0.53* | 3.03 | **0.17** | *-0.47* | *0.81* | -1.53 | **-0.08** | *-0.73* | *0.56* |
| Serial3Responses | 0.00 | **0.00** | *-0.64* | *0.64* | 1.23 | **0.05** | *-0.59* | *0.69* | -0.36 | **-0.01** | *-0.65* | *0.63* | 0.17 | **0.01** | *-0.63* | *0.65* | 4.28 | **0.16** | *-0.48* | *0.80* |
| Serial3Errors | 0.22 | **0.07** | *-0.57* | *0.71* | -0.89 | **-0.29** | *-0.93* | *0.36* | -0.50 | **-0.16** | *-0.80* | *0.48* | -0.06 | **-0.02** | *-0.66* | *0.62* | -0.23 | **-0.07** | *-0.71* | *0.57* |
| Serial7Responses | 5.40 | **0.30** | *-0.34* | *0.94* | 5.50 | **0.31** | *-0.34* | *0.95* | 6.30 | **0.35** | *-0.30* | *0.99* | 7.56 | **0.42** | *-0.23* | *1.07* | 7.82 | **0.43** | *-0.21* | *1.08* |
| Serial7Errors | 0.49 | **0.15** | *-0.50* | *0.79* | 0.54 | **0.16** | *-0.48* | *0.80* | 0.27 | **0.08** | *-0.56* | *0.72* | -0.93 | **-0.28** | *-0.92* | *0.37* | -0.83 | **-0.25** | -0.89 | 0.40 |

Table 35. Kennedy et al., (2002): comparing effect sizes between Placebo and Ginkgo/Ginseng

|  | Baseline | | | | 1 hour | | | | 2.5 hours | | | | 4 hours | | | | 6 hours | | | |
| --- | --- | --- | --- | --- | --- | --- | --- | --- | --- | --- | --- | --- | --- | --- | --- | --- | --- | --- | --- | --- |
|  | Mean | *d* | LCI | UCI | Mean | *d* | LCI | UCI | Mean | *d* | LCI | UCI | Mean | *d* | LCI | UCI | Mean | *d* | LCI | UCI |
| Quality of Memory | -19.04 | **-0.24** | *-0.88* | *0.40* | 18.13 | **0.23** | *-0.41* | *0.87* | 24.99 | **0.32** | *-0.33* | *0.96* | 10.28 | **0.13** | *-0.51* | *0.77* | -13.39 | **-0.17** | *-0.81* | *0.47* |
| Secondary Memory | -7.67 | **-0.13** | *-0.77* | *0.51* | 18.62 | **0.32** | *-0.32* | *0.97* | 29.04 | **0.50** | *-0.15* | *1.15* | 11.25 | **0.19** | *-0.45* | *0.84* | -15.50 | **-0.27** | *-0.91* | *0.38* |
| Working Memory | -11.37 | **-0.39** | *-1.04* | *0.25* | -0.50 | **-0.02** | *-0.66* | *0.62* | -4.05 | **-0.14** | *-0.78* | *0.50* | -0.98 | **-0.03** | *-0.67* | *0.61* | 2.11 | **0.07** | *-0.57* | *0.71* |
| Speed of Memory | 6.41 | **0.02** | *-0.62* | *0.66* | 15.32 | **0.04** | *-0.60* | *0.68* | 8.29 | **0.02** | *-0.62* | *0.66* | -27.42 | **-0.07** | *-0.71* | *0.57* | 32.49 | **0.08** | *-0.56* | *0.72* |
| Quality of Attention | -0.50 | **-0.14** | *-0.79* | *0.50* | 0.40 | **0.12** | *-0.52* | *0.76* | 0.65 | **0.19** | *-0.45* | *0.83* | -0.40 | **-0.12** | *-0.76* | *0.52* | 0.00 | **0.00** | *-0.64* | *0.64* |
| Speed of Attention | 6.43 | **0.00** | *-0.64* | *0.64* | 12.40 | **0.00** | *-0.64* | *0.64* | 12.10 | **0.00** | *-0.64* | *0.64* | 36.09 | **0.01** | *-0.63* | *0.65* | -7.22 | **0.00** | *-0.64* | *0.64* |
| Alert | -0.43 | **-0.02** | *-0.67* | *0.62* | -2.74 | **-0.16** | *-0.80* | *0.48* | -2.93 | **-0.17** | *-0.81* | *0.47* | 0.70 | **0.04** | *-0.60* | *0.68* | -0.31 | **-0.02** | *-0.66* | *0.62* |
| Content | -4.89 | **-0.30** | *-0.95* | *0.34* | -1.80 | **-0.11** | *-0.75* | *0.53* | 2.27 | **0.14** | *-0.50* | *0.78* | 1.06 | **0.07** | *-0.57* | *0.71* | 3.01 | **0.19** | *-0.45* | *0.83* |
| Calm | 1.45 | **0.09** | *-0.55* | *0.73* | 0.25 | **0.02** | *-0.62* | *0.66* | 3.56 | **0.23** | *-0.42* | *0.87* | 2.25 | **0.14** | *-0.50* | *0.78* | -1.38 | **-0.09** | *-0.73* | *0.55* |
| Serial3Responses | -1.45 | **-0.05** | *-0.69* | *0.59* | -1.56 | **-0.06** | *-0.70* | *0.58* | -2.09 | **-0.08** | *-0.72* | *0.56* | 1.61 | **0.06** | *-0.58* | *0.70* | 4.72 | **0.18** | *-0.46* | *0.82* |
| Serial3Errors | -0.11 | **-0.03** | *-0.67* | *0.61* | -0.27 | **-0.08** | *-0.72* | *0.56* | -0.67 | **-0.19** | *-0.83* | *0.45* | -0.16 | **-0.05** | *-0.69* | *0.59* | -1.39 | **-0.40** | *-1.04* | *0.25* |
| Serial7Responses | 1.14 | **0.06** | *-0.58* | *0.70* | -0.13 | **-0.01** | *-0.65* | *0.63* | 2.20 | **0.12** | *-0.52* | *0.76* | 4.62 | **0.25** | *-0.40* | *0.89* | 1.66 | **0.09** | *-0.55* | *0.73* |
| Serial7Errors | 0.31 | **0.09** | *-0.55* | *0.73* | 0.47 | **0.14** | *-0.50* | *0.78* | 0.88 | **0.26** | *-0.39* | *0.90* | -0.21 | **-0.06** | *-0.70* | *0.58* | -1.48 | **-0.43** | *-1.08* | *0.22* |

Table 36. Kennedy et al., (2002): comparing effect sizes between Ginkgo and Ginseng

|  | Baseline | | | | 1 hour | | | | 2.5 hours | | | | 4 hours | | | | 6 hours | | | |
| --- | --- | --- | --- | --- | --- | --- | --- | --- | --- | --- | --- | --- | --- | --- | --- | --- | --- | --- | --- | --- |
|  | Mean | *d* | LCI | UCI | Mean | *d* | LCI | UCI | Mean | *d* | LCI | UCI | Mean | *d* | LCI | UCI | Mean | *d* | LCI | UCI |
| Quality of Memory | 6.41 | **0.09** | *-0.55* | *0.73* | -14.81 | **-0.21** | *-0.85* | *0.43* | 0.36 | **0.01** | *-0.64* | *0.65* | 19.45 | **0.28** | *-0.37* | *0.92* | -4.55 | **-0.06** | *-0.71* | *0.58* |
| Secondary Memory | 6.50 | **0.11** | *-0.53* | *0.75* | -9.58 | **-0.16** | *-0.80* | *0.48* | 4.90 | **0.08** | *-0.56* | *0.72* | 23.75 | **0.40** | *-0.25* | *1.04* | 4.25 | **0.07** | *-0.57* | *0.71* |
| Working Memory | -0.09 | **0.00** | *-0.64* | *0.64* | -5.23 | **-0.25** | *-0.90* | *0.39* | -4.54 | **-0.22** | *-0.86* | *0.42* | -4.30 | **-0.21** | *-0.85* | *0.43* | -8.80 | **-0.42** | *-1.07* | *0.22* |
| Speed of Memory | 44.88 | **0.12** | *-0.52* | *0.76* | -13.56 | **-0.04** | *-0.68* | *0.61* | -22.88 | **-0.06** | *-0.70* | *0.58* | -103.70 | **-0.27** | *-0.91* | *0.38* | -16.07 | **-0.04** | *-0.68* | *0.60* |
| Quality of Attention | -1.65 | **-0.38** | *-1.02* | *0.27* | -1.60 | **-0.36** | *-1.01* | *0.28* | -0.75 | **-0.17** | *-0.81* | *0.47* | 0.10 | **0.02** | *-0.62* | *0.66* | -0.10 | **-0.02** | *-0.66* | *0.62* |
| Speed of Attention | -1.92 | **-0.01** | *-0.65* | *0.63* | 1.21 | **0.01** | *-0.63* | *0.65* | -12.83 | **-0.10** | *-0.74* | *0.54* | -4.19 | **-0.03** | *-0.67* | *0.61* | 3.25 | **0.02** | *-0.62* | *0.66* |
| Alert | 1.81 | **0.10** | *-0.54* | *0.74* | -1.64 | **-0.09** | *-0.73* | *0.55* | -0.59 | **-0.03** | *-0.67* | *0.61* | -0.87 | **-0.05** | *-0.69* | *0.59* | -4.97 | **-0.28** | *-0.92* | *0.36* |
| Content | 2.83 | **0.18** | *-0.46* | *0.82* | -1.50 | **-0.10** | *-0.74* | *0.54* | 2.44 | **0.16** | *-0.48* | *0.80* | -2.14 | **-0.14** | *-0.78* | *0.50* | -1.76 | **-0.11** | *-0.75* | *0.53* |
| Calm | -1.95 | **-0.12** | *-0.76* | *0.53* | -3.88 | **-0.23** | *-0.87* | *0.41* | -1.95 | **-0.12** | *-0.76* | *0.53* | 1.01 | **0.06** | *-0.58* | *0.70* | 5.43 | **0.32** | *-0.32* | *0.96* |
| Serial3Responses | 1.17 | **0.04** | *-0.60* | *0.68* | 3.51 | **0.13** | *-0.51* | *0.77* | 0.64 | **0.02** | *-0.62* | *0.66* | -1.72 | **-0.06** | *-0.70* | *0.58* | 1.39 | **0.05** | *-0.59* | *0.69* |
| Serial3Errors | -0.78 | **-0.24** | *-0.88* | *0.40* | -2.39 | **-0.73** | *-1.39* | *-0.07* | -0.79 | **-0.24** | *-0.88* | *0.40* | 1.60 | **0.49** | *-0.16* | *1.14* | 0.99 | **0.30** | *-0.34* | *0.95* |
| Serial7Responses | 6.54 | **0.33** | *-0.31* | *0.98* | 4.27 | **0.22** | *-0.43* | *0.86* | 6.23 | **0.31** | *-0.33* | *0.96* | 5.33 | **0.27** | *-0.37* | *0.91* | 4.12 | **0.21** | *-0.43* | *0.85* |
| Serial7Errors | 0.40 | **0.14** | *-0.50* | *0.78* | -0.18 | **-0.06** | *-0.70* | *0.58* | -0.18 | **-0.06** | *-0.70* | *0.58* | 0.24 | **0.09** | *-0.55* | *0.73* | -1.23 | **-0.44** | *-1.09* | *0.21* |

Table 37. Kennedy et al., (2002): comparing effect sizes between Ginkgo and Ginkgo/Ginseng

|  | Baseline | | | | 1 hour | | | | 2.5 hours | | | | 4 hours | | | | 6 hours | | | |
| --- | --- | --- | --- | --- | --- | --- | --- | --- | --- | --- | --- | --- | --- | --- | --- | --- | --- | --- | --- | --- |
|  | Mean | *d* | LCI | UCI | Mean | *d* | LCI | UCI | Mean | *d* | LCI | UCI | Mean | *d* | LCI | UCI | Mean | *d* | LCI | UCI |
| Quality of Memory | -8.34 | **-0.10** | *-0.74* | *0.54* | 10.25 | **0.13** | *-0.51* | *0.77* | 14.49 | **0.18** | *-0.46* | *0.82* | 7.08 | **0.09** | *-0.55* | *0.73* | -32.06 | **-0.40** | *-1.04* | *0.25* |
| Secondary Memory | 5.17 | **0.08** | *-0.56* | *0.72* | 8.13 | **0.13** | *-0.51* | *0.77* | 22.32 | **0.36** | *-0.29* | *1.00* | 8.76 | **0.14** | *-0.50* | *0.78* | -30.33 | **-0.49** | *-1.14* | *0.16* |
| Working Memory | -13.50 | **-0.46** | *-1.11* | *0.19* | 2.13 | **0.07** | *-0.57* | *0.71* | -7.82 | **-0.26** | *-0.91* | *0.38* | -1.67 | **-0.06** | *-0.70* | *0.58* | -1.72 | **-0.06** | *-0.70* | *0.58* |
| Speed of Memory | 9.14 | **0.02** | *-0.62* | *0.66* | -12.17 | **-0.03** | *-0.67* | *0.61* | 23.79 | **0.06** | *-0.58* | *0.70* | -79.74 | **-0.21** | *-0.85* | *0.43* | -16.86 | **-0.04** | *-0.68* | *0.60* |
| Quality of Attention | -0.05 | **-0.01** | *-0.65* | *0.63* | -0.40 | **-0.10** | *-0.74* | *0.54* | -0.55 | **-0.14** | *-0.78* | *0.50* | 0.20 | **0.05** | *-0.59* | *0.69* | 1.10 | **0.28** | *-0.36* | *0.92* |
| Speed of Attention | 0.45 | **0.00** | *-0.64* | *0.64* | 20.59 | **0.01** | *-0.63* | *0.65* | -1.86 | **0.00** | *-0.64* | *0.64* | 10.47 | **0.00** | *-0.64* | *0.64* | 9.55 | **0.00** | *-0.64* | *0.64* |
| Alert | 4.47 | **0.26** | *-0.39* | *0.90* | -3.48 | **-0.20** | *-0.84* | *0.44* | -3.72 | **-0.21** | *-0.86* | *0.43* | -1.55 | **-0.09** | *-0.73* | *0.55* | -3.77 | **-0.22** | *-0.86* | *0.43* |
| Content | 2.70 | **0.15** | *-0.49* | *0.79* | -0.68 | **-0.04** | *-0.68* | *0.60* | 5.47 | **0.31** | *-0.33* | *0.96* | 0.62 | **0.04** | *-0.60* | *0.68* | 2.93 | **0.17** | *-0.47* | *0.81* |
| Calm | -0.95 | **-0.07** | *-0.71* | *0.57* | -0.52 | **-0.04** | *-0.68* | *0.60* | 3.58 | **0.25** | *-0.39* | *0.89* | 0.23 | **0.02** | *-0.62* | *0.66* | 5.58 | **0.39** | *-0.26* | *1.03* |
| Serial3Responses | -0.28 | **-0.01** | *-0.65* | *0.63* | 0.72 | **0.03** | *-0.61* | *0.67* | -1.09 | **-0.04** | *-0.68* | *0.60* | -0.28 | **-0.01** | *-0.65* | *0.63* | 1.83 | **0.07** | *-0.57* | *0.71* |
| Serial3Errors | -1.11 | **-0.30** | *-0.95* | *0.34* | -1.77 | **-0.48** | *-1.13* | *0.17* | -0.96 | **-0.26** | *-0.91* | *0.38* | 1.50 | **0.41** | *-0.24* | *1.06* | -0.17 | **-0.05** | *-0.69* | *0.59* |
| Serial7Responses | 2.28 | **0.11** | *-0.53* | *0.75* | -1.36 | **-0.07** | *-0.71* | *0.57* | 2.13 | **0.10** | *-0.54* | *0.74* | 2.39 | **0.12** | *-0.52* | *0.76* | -2.04 | **-0.10** | *-0.74* | *0.54* |
| Serial7Errors | 0.22 | **0.08** | *-0.56* | *0.72* | -0.25 | **-0.09** | *-0.73* | *0.55* | 0.43 | **0.15** | *-0.49* | *0.79* | 0.96 | **0.33** | *-0.31* | *0.98* | -1.88 | -0.65 | *-1.31* | *0.01* |

Table 38. Kennedy et al., (2002): comparing effect sizes between Ginseng and Ginkgo/Ginseng

|  | Baseline | | | | 1 hour | | | | 2.5 hours | | | | 4 hours | | | | 6 hours | | | |
| --- | --- | --- | --- | --- | --- | --- | --- | --- | --- | --- | --- | --- | --- | --- | --- | --- | --- | --- | --- | --- |
|  | Mean | *d* | LCI | UCI | Mean | *d* | LCI | UCI | Mean | *d* | LCI | UCI | Mean | *d* | LCI | UCI | Mean | *d* | LCI | UCI |
| Quality of Memory | -14.75 | **-0.19** | *-0.83* | *0.45* | 25.06 | **0.32** | *-0.32* | *0.96* | 14.13 | **0.18** | *-0.46* | *0.82* | -12.37 | **-0.16** | *-0.80* | *0.48* | -27.51 | **-0.35** | *-1.00* | *0.29* |
| Secondary Memory | -1.33 | **-0.02** | *-0.66* | *0.62* | 17.71 | **0.30** | *-0.34* | *0.95* | 17.42 | **0.30** | *-0.34* | *0.94* | -14.99 | **-0.26** | *-0.90* | *0.38* | -34.58 | **-0.59** | *-1.25* | *0.06* |
| Working Memory | -13.41 | **-0.49** | *-1.14* | *0.16* | 7.36 | **0.27** | *-0.37* | *0.91* | -3.28 | **-0.12** | *-0.76* | *0.52* | 2.63 | **0.10** | *-0.54* | *0.74* | 7.08 | **0.26** | *-0.38* | *0.90* |
| Speed of Memory | -35.74 | **-0.09** | *-0.73* | *0.55* | 1.39 | **0.00** | *-0.64* | *0.64* | 46.67 | **0.12** | *-0.52* | *0.76* | 23.96 | **0.06** | *-0.58* | *0.70* | -0.79 | **0.00** | *-0.64* | *0.64* |
| Quality of Attention | 1.60 | **0.39** | *-0.26* | *1.03* | 1.20 | **0.29** | *-0.35* | *0.93* | 0.20 | **0.05** | *-0.59* | *0.69* | 0.10 | **0.02** | *-0.62* | *0.66* | 1.20 | **0.29** | *-0.35* | *0.93* |
| Speed of Attention | 2.37 | **0.00** | *-0.64* | *0.64* | 19.38 | **0.01** | *-0.63* | *0.65* | 10.97 | **0.00** | *-0.64* | *0.64* | 14.66 | **0.00** | *-0.64* | *0.64* | 6.30 | **0.00** | *-0.64* | *0.64* |
| Alert | 2.66 | **0.15** | *-0.49* | *0.79* | -1.84 | **-0.10** | *-0.75* | *0.54* | -3.13 | **-0.18** | *-0.82* | *0.46* | -0.68 | **-0.04** | *-0.68* | *0.60* | 1.20 | **0.07** | *-0.57* | *0.71* |
| Content | -0.13 | **-0.01** | *-0.65* | *0.63* | 0.82 | **0.05** | *-0.59* | *0.69* | 3.03 | **0.19** | *-0.45* | *0.83* | 2.76 | **0.17** | *-0.47* | *0.81* | 4.69 | **0.29** | *-0.35* | *0.93* |
| Calm | 1.00 | **0.06** | *-0.58* | *0.70* | 3.36 | **0.19** | *-0.45* | *0.83* | 5.53 | **0.32** | *-0.33* | *0.96* | -0.78 | **-0.04** | *-0.68* | *0.60* | 0.15 | **0.01** | *-0.63* | *0.65* |
| Serial3Responses | -1.45 | **-0.06** | *-0.70* | *0.58* | -2.79 | **-0.11** | *-0.75* | *0.53* | -1.73 | **-0.07** | *-0.71* | *0.57* | 1.44 | **0.06** | *-0.59* | *0.70* | 0.44 | **0.02** | *-0.62* | *0.66* |
| Serial3Errors | -0.33 | **-0.11** | *-0.75* | *0.53* | 0.62 | **0.21** | *-0.43* | *0.85* | -0.17 | **-0.06** | *-0.70* | *0.58* | -0.10 | **-0.03** | *-0.67* | *0.61* | -1.16 | **-0.39** | *-1.04* | *0.25* |
| Serial7Responses | -4.26 | **-0.23** | *-0.87* | *0.41* | -5.63 | **-0.30** | *-0.95* | *0.34* | -4.10 | **-0.22** | *-0.86* | *0.42* | -2.94 | **-0.16** | *-0.80* | *0.48* | -6.16 | **-0.33** | *-0.97* | *0.31* |
| Serial7Errors | -0.18 | **-0.06** | *-0.70* | *0.58* | -0.07 | **-0.02** | *-0.66* | *0.62* | 0.61 | **0.19** | *-0.45* | *0.83* | 0.72 | **0.22** | *-0.42* | *0.87* | -0.65 | **-0.20** | *-0.84* | *0.44* |

Table 39. Kennedy et al., (2002): comparing effect sizes between Baseline and 1 hour

|  | Placebo | | | | Ginkgo | | | | Ginseng | | | | Gink_Gin | | | |
| --- | --- | --- | --- | --- | --- | --- | --- | --- | --- | --- | --- | --- | --- | --- | --- | --- |
|  | Mean | *d* | LCI | UCI | Mean | *d* | LCI | UCI | Mean | *d* | LCI | UCI | Mean | *d* | LCI | UCI |
| Quality of Memory | -13.39 | **-0.20** | *-0.84* | *0.45* | 5.19 | **0.07** | *-0.57* | *0.71* | -16.03 | **-0.24** | *-0.88* | *0.40* | 23.78 | **0.27** | *-0.37* | *0.91* |
| Secondary Memory | -15.33 | **-0.28** | *-0.92* | *0.36* | 8.00 | **0.13** | *-0.52* | *0.77* | -8.08 | **-0.15** | *-0.79* | *0.50* | 10.96 | **0.18** | *-0.46* | *0.82* |
| Working Memory | 1.95 | **0.09** | *-0.55* | *0.73* | -2.81 | **-0.12** | *-0.76* | *0.52* | -7.95 | **-0.46** | *-1.11* | *0.18* | 12.82 | **0.37** | *-0.27* | *1.02* |
| Speed of Memory | 11.32 | **0.03** | *-0.61* | *0.67* | 41.54 | **0.11** | *-0.53* | *0.75* | -16.90 | **-0.04** | *-0.68* | *0.60* | 20.23 | **0.05** | *-0.59* | *0.69* |
| Quality of Attention | -1.10 | **-0.34** | *-0.99* | *0.30* | 0.15 | **0.04** | *-0.60* | *0.68* | 0.20 | **0.04** | *-0.60* | *0.68* | -0.20 | **-0.05** | *-0.69* | *0.59* |
| Speed of Attention | 11.63 | **0.09** | *-0.55* | *0.73* | -2.54 | **-0.02** | *-0.66* | *0.62* | 0.59 | **0.00** | *-0.64* | *0.64* | 17.60 | **0.00** | *-0.64* | *0.64* |
| Alert | 3.74 | **0.22** | *-0.42* | *0.86* | 9.38 | **0.53** | *-0.12* | *1.18* | 5.93 | **0.33** | *-0.31* | *0.98* | 1.43 | **0.08** | *-0.56* | *0.72* |
| Content | -1.73 | **-0.13** | *-0.77* | *0.51* | 4.74 | **0.28** | *-0.36* | *0.92* | 0.41 | **0.03** | *-0.61* | *0.67* | 1.36 | **0.08** | *-0.57* | *0.72* |
| Calm | 2.38 | **0.15** | *-0.50* | *0.79* | 0.75 | **0.05** | *-0.59* | *0.69* | -1.18 | **-0.06** | *-0.70* | *0.58* | 1.18 | **0.08** | *-0.56* | *0.72* |
| Serial3Responses | 4.44 | **0.16** | *-0.48* | *0.80* | 3.33 | **0.12** | *-0.52* | *0.76* | 5.67 | **0.22** | *-0.43* | *0.86* | 4.33 | **0.17** | *-0.47* | *0.81* |
| Serial3Errors | 0.83 | **0.23** | *-0.41* | *0.87* | 1.33 | **0.34** | *-0.31* | *0.98* | -0.28 | **-0.11** | *-0.75* | *0.53* | 0.67 | **0.20** | *-0.44* | *0.84* |
| Serial7Responses | 2.74 | **0.15** | *-0.49* | *0.79* | 5.11 | **0.24** | *-0.41* | *0.88* | 2.84 | **0.16** | *-0.48* | *0.80* | 1.47 | **0.08** | *-0.56* | *0.72* |
| Serial7Errors | -0.05 | **-0.01** | *-0.65* | *0.63* | 0.58 | **0.24** | *-0.40* | *0.88* | 0.00 | **0.00** | *-0.64* | *0.64* | 0.11 | **0.03** | *-0.61* | *0.67* |

Table 40. Kennedy et al., (2002): comparing effect sizes between Baseline and 2.5 hours

|  | Placebo | | | | Ginkgo | | | | Ginseng | | | | Gink_Gin | | | |
| --- | --- | --- | --- | --- | --- | --- | --- | --- | --- | --- | --- | --- | --- | --- | --- | --- |
|  | Mean | *d* | LCI | UCI | Mean | *d* | LCI | UCI | Mean | *d* | LCI | UCI | Mean | *d* | LCI | UCI |
| Quality of Memory | -27.90 | **-0.41** | *-1.05* | *0.24* | -6.70 | **-0.09** | *-0.73* | *0.55* | -12.75 | **-0.19** | *-0.83* | *0.45* | 16.13 | **0.18** | *-0.46* | *0.82* |
| Secondary Memory | -25.04 | **-0.45** | *-1.10* | *0.19* | -5.48 | **-0.09** | *-0.73* | *0.55* | -7.08 | **-0.13** | *-0.77* | *0.51* | 11.67 | **0.19** | *-0.45* | *0.83* |
| Working Memory | -2.86 | **-0.13** | *-0.77* | *0.51* | -1.22 | **-0.05** | *-0.69* | *0.59* | -5.67 | **-0.33** | *-0.98* | *0.31* | 4.46 | **0.13** | *-0.51* | *0.77* |
| Speed of Memory | 33.86 | **0.08** | *-0.56* | *0.73* | 21.09 | **0.06** | *-0.58* | *0.70* | -46.67 | **-0.12** | *-0.76* | *0.52* | 35.74 | **0.09** | *-0.55* | *0.73* |
| Quality of Attention | -1.70 | **-0.53** | *-1.18* | *0.12* | -0.05 | **-0.01** | *-0.65* | *0.63* | 0.85 | **0.19** | *-0.46* | *0.83* | -0.55 | **-0.15** | *-0.79* | *0.49* |
| Speed of Attention | 8.03 | **0.06** | *-0.58* | *0.70* | 16.01 | **0.12** | *-0.52* | *0.76* | 5.10 | **0.04** | *-0.60* | *0.68* | 13.70 | **0.00** | *-0.64* | *0.64* |
| Alert | 3.02 | **0.18** | *-0.47* | *0.82* | 8.71 | **0.49** | *-0.16* | *1.14* | 6.31 | **0.35** | *-0.29* | *1.00* | 0.52 | **0.03** | *-0.61* | *0.67* |
| Content | -3.91 | **-0.28** | *-0.93* | *0.36* | 0.48 | **0.03** | *-0.61* | *0.67* | 0.09 | **0.01** | *-0.63* | *0.65* | 3.25 | **0.18** | *-0.46* | *0.82* |
| Calm | -0.08 | **0.00** | *-0.65* | *0.64* | -2.50 | **-0.18** | *-0.82* | *0.46* | -2.50 | **-0.13** | *-0.77* | *0.51* | 2.03 | **0.14** | *-0.51* | *0.78* |
| Serial3Responses | 6.42 | **0.23** | *-0.41* | *0.88* | 6.59 | **0.24** | *-0.41* | *0.88* | 6.06 | **0.23** | *-0.41* | *0.87* | 5.78 | **0.22** | *-0.42* | *0.86* |
| Serial3Errors | 0.89 | **0.25** | *-0.40* | *0.89* | 0.18 | **0.05** | *-0.59* | *0.69* | 0.17 | **0.07** | *-0.57* | *0.71* | 0.33 | **0.10** | *-0.54* | *0.74* |
| Serial7Responses | 2.68 | **0.15** | *-0.49* | *0.79* | 3.89 | **0.18** | *-0.46* | *0.82* | 3.58 | **0.20** | *-0.44* | *0.84* | 3.74 | **0.19** | *-0.45* | *0.83* |
| Serial7Errors | 0.11 | **0.03** | *-0.61* | *0.67* | 0.47 | **0.19** | *-0.45* | *0.84* | -0.11 | **-0.04** | *-0.68* | *0.61* | 0.68 | **0.21** | *-0.44* | *0.85* |

Table 41. Kennedy et al., (2002): comparing effect sizes between Baseline and 4 hours

|  | Placebo | | | | Ginkgo | | | | Ginseng | | | | Gink_Gin | | | |
| --- | --- | --- | --- | --- | --- | --- | --- | --- | --- | --- | --- | --- | --- | --- | --- | --- |
|  | Mean | *d* | LCI | UCI | Mean | *d* | LCI | UCI | Mean | *d* | LCI | UCI | Mean | *d* | LCI | UCI |
| Quality of Memory | -36.27 | **-0.53** | *-1.18* | *0.12* | -22.37 | **-0.31** | *-0.95* | *0.34* | -9.33 | **-0.14** | *-0.78* | *0.50* | -6.95 | **-0.08** | *-0.72* | *0.56* |
| Secondary Memory | -30.75 | **-0.56** | *-1.21* | *0.09* | -15.42 | **-0.24** | *-0.88* | *0.40* | 1.83 | **0.03** | *-0.61* | *0.67* | -11.83 | **-0.19** | *-0.84* | *0.45* |
| Working Memory | -5.51 | **-0.25** | *-0.89* | *0.39* | -6.95 | **-0.29** | *-0.94* | *0.35* | -11.16 | **-0.65** | *-1.31* | *0.01* | 4.88 | **0.14** | *-0.50* | *0.78* |
| Speed of Memory | 53.37 | **0.13** | *-0.51* | *0.77* | 108.42 | **0.29** | *-0.36* | *0.93* | -40.16 | **-0.10** | *-0.74* | *0.54* | 19.54 | **0.05** | *-0.59* | *0.69* |
| Quality of Attention | -1.15 | **-0.36** | *-1.00* | *0.29* | -1.30 | **-0.31** | *-0.95* | *0.33* | 0.45 | **0.10** | *-0.54* | *0.74* | -1.05 | **-0.29** | *-0.93* | *0.36* |
| Speed of Attention | 16.74 | **0.13** | *-0.51* | *0.77* | 36.38 | **0.27** | *-0.38* | *0.91* | 34.11 | **0.27** | *-0.38* | *0.91* | 46.40 | **0.01** | *-0.63* | *0.65* |
| Alert | 2.21 | **0.13** | *-0.51* | *0.77* | 9.36 | **0.53** | *-0.12* | *1.18* | 6.68 | **0.37** | *-0.27* | *1.02* | 3.34 | **0.19** | *-0.45* | *0.84* |
| Content | -3.53 | **-0.26** | *-0.90* | *0.39* | 4.50 | **0.26** | *-0.38* | *0.91* | -0.47 | **-0.03** | *-0.67* | *0.61* | 2.42 | **0.13** | *-0.51* | *0.77* |
| Calm | -1.20 | **-0.07** | *-0.71* | *0.57* | -1.58 | **-0.11** | *-0.75* | *0.53* | 1.38 | **0.07** | *-0.57* | *0.71* | -0.40 | **-0.03** | *-0.67* | *0.61* |
| Serial3Responses | 3.22 | **0.12** | *-0.52* | *0.76* | 6.28 | **0.22** | *-0.42* | *0.87* | 3.39 | **0.13** | *-0.51* | *0.77* | 6.28 | **0.24** | *-0.40* | *0.88* |
| Serial3Errors | 2.22 | **0.61** | *-0.04* | *1.27* | -0.44 | **-0.11** | *-0.75* | *0.53* | 1.94 | **0.79** | *0.12* | *1.45* | 2.17 | **0.65** | *-0.01* | *1.30* |
| Serial7Responses | 0.84 | **0.05** | *-0.59* | *0.69* | 4.21 | **0.20** | *-0.45* | *0.84* | 3.00 | **0.17** | *-0.47* | *0.81* | 4.32 | **0.22** | *-0.42* | *0.86* |
| Serial7Errors | 1.26 | **0.35** | *-0.29* | *1.00* | 0.00 | **0.00** | *-0.64* | *0.64* | -0.16 | **-0.05** | *-0.69* | *0.59* | 0.74 | **0.22** | *-0.42* | *0.87* |

Table 42. Kennedy et al., (2002): comparing effect sizes between Baseline and 6 hours

|  | Placebo | | | | Ginkgo | | | | Ginseng | | | | Gink_Gin | | | |
| --- | --- | --- | --- | --- | --- | --- | --- | --- | --- | --- | --- | --- | --- | --- | --- | --- |
|  | Mean | *d* | LCI | UCI | Mean | *d* | LCI | UCI | Mean | *d* | LCI | UCI | Mean | *d* | LCI | UCI |
| Quality of Memory | -30.68 | **-0.45** | *-1.10* | *0.20* | -1.31 | **-0.02** | *-0.66* | *0.62* | -12.27 | **-0.18** | *-0.82* | *0.46* | -25.03 | **-0.28** | *-0.93* | *0.36* |
| Secondary Memory | -21.50 | **-0.39** | *-1.04* | *0.26* | 6.17 | **0.10** | *-0.54* | *0.74* | 3.92 | **0.07** | *-0.57* | *0.71* | -29.33 | **-0.48** | *-1.13* | *0.17* |
| Working Memory | -9.18 | **-0.41** | *-1.06* | *0.23* | -7.48 | **-0.31** | *-0.96* | *0.33* | -16.19 | **-0.95** | *-1.62* | *-0.27* | 4.30 | **0.13** | *-0.52* | *0.77* |
| Speed of Memory | -41.54 | **-0.10** | *-0.74* | *0.54* | 10.54 | **0.03** | *-0.61* | *0.67* | -50.41 | **-0.13** | *-0.77* | *0.51* | -15.46 | **-0.04** | *-0.68* | *0.60* |
| Quality of Attention | -0.35 | **-0.11** | *-0.75* | *0.53* | -1.00 | **-0.24** | *-0.88* | *0.40* | 0.55 | **0.12** | *-0.52* | *0.76* | 0.15 | **0.04** | *-0.60* | *0.68* |
| Speed of Attention | 36.52 | **0.29** | *-0.36* | *0.93* | 13.77 | **0.10** | *-0.54* | *0.74* | 18.94 | **0.15** | *-0.49* | *0.79* | 22.87 | **0.00** | *-0.64* | *0.65* |
| Alert | 2.41 | **0.14** | *-0.50* | *0.78* | 10.77 | **0.61** | *-0.04* | *1.27* | 3.99 | **0.22** | *-0.42* | *0.87* | 2.53 | **0.15** | *-0.49* | *0.79* |
| Content | -4.98 | **-0.36** | *-1.01* | *0.28* | 2.69 | **0.16** | *-0.48* | *0.80* | -1.90 | **-0.14** | *-0.78* | *0.51* | 2.92 | **0.16** | *-0.48* | *0.80* |
| Calm | 2.23 | **0.14** | *-0.50* | *0.78* | -7.13 | **-0.51** | *-1.16* | *0.14* | 0.25 | **0.01** | *-0.63* | *0.65* | -0.60 | **-0.04** | *-0.68* | *0.60* |
| Serial3Responses | 1.72 | **0.06** | *-0.58* | *0.70* | 5.78 | **0.21** | *-0.44* | *0.85* | 6.00 | **0.23** | *-0.41* | *0.87* | 7.89 | **0.30** | *-0.34* | *0.95* |
| Serial3Errors | 1.89 | **0.52** | *-0.13* | *1.17* | -0.33 | **-0.08** | *-0.72* | *0.56* | 1.44 | **0.59** | *-0.07* | *1.24* | 0.61 | **0.18** | *-0.46* | *0.82* |
| Serial7Responses | 1.53 | **0.08** | *-0.56* | *0.72* | 6.37 | **0.30** | *-0.35* | *0.94* | 3.95 | **0.22** | *-0.42* | *0.86* | 2.05 | **0.11** | *-0.54* | *0.75* |
| Serial7Errors | 1.32 | **0.37** | *-0.28* | *1.01* | 1.63 | **0.67** | *0.02* | *1.33* | 0.00 | **0.00** | *-0.64* | *0.64* | -0.47 | **-0.14** | *-0.78* | *0.50* |

Table 43. Kennedy et al., (2002): comparing effect sizes between 1 hour and 2.5 hours

|  | Placebo | | | | Ginkgo | | | | Ginseng | | | | Gink_Gin | | | |
| --- | --- | --- | --- | --- | --- | --- | --- | --- | --- | --- | --- | --- | --- | --- | --- | --- |
|  | Mean | *d* | LCI | UCI | Mean | *d* | LCI | UCI | Mean | *d* | LCI | UCI | Mean | *d* | LCI | UCI |
| Quality of Memory | -14.51 | **-0.21** | *-0.85* | *0.43* | -11.89 | **-0.16** | *-0.80* | *0.48* | 3.28 | **0.05** | *-0.59* | *0.69* | -7.65 | **-0.09** | *-0.73* | *0.55* |
| Secondary Memory | -9.71 | **-0.18** | *-0.82* | *0.47* | -13.48 | **-0.21** | *-0.85* | *0.43* | 1.00 | **0.02** | *-0.62* | *0.66* | 0.71 | **0.01** | *-0.63* | *0.65* |
| Working Memory | -4.81 | **-0.22** | *-0.86* | *0.42* | 1.59 | **0.07** | *-0.57* | *0.71* | 2.28 | **0.13** | *-0.51* | *0.77* | -8.36 | **-0.24** | *-0.89* | *0.40* |
| Speed of Memory | 22.54 | **0.06** | *-0.58* | *0.70* | -20.45 | **-0.05** | *-0.69* | *0.59* | -29.77 | **-0.08** | *-0.72* | *0.56* | 15.51 | **0.04** | *-0.60* | *0.68* |
| Quality of Attention | -0.60 | **-0.19** | *-0.83* | *0.46* | -0.20 | **-0.05** | *-0.69* | *0.59* | 0.65 | **0.14** | *-0.50* | *0.78* | -0.35 | **-0.10** | *-0.74* | *0.55* |
| Speed of Attention | -3.60 | **-0.03** | *-0.67* | *0.61* | 18.55 | **0.14** | *-0.51* | *0.78* | 4.51 | **0.04** | *-0.60* | *0.68* | -3.90 | **0.00** | *-0.64* | *0.64* |
| Alert | -0.72 | **-0.04** | *-0.68* | *0.60* | -0.67 | **-0.04** | *-0.68* | *0.60* | 0.38 | **0.02** | *-0.62* | *0.66* | -0.91 | **-0.05** | *-0.69* | *0.59* |
| Content | -2.18 | **-0.16** | *-0.80* | *0.48* | -4.26 | **-0.25** | *-0.89* | *0.39* | -0.32 | **-0.02** | *-0.66* | *0.62* | 1.89 | **0.10** | *-0.54* | *0.74* |
| Calm | -2.46 | **-0.15** | *-0.79* | *0.49* | -3.25 | **-0.23** | *-0.88* | *0.41* | -1.32 | **-0.07** | *-0.71* | *0.57* | 0.85 | **0.06** | *-0.58* | *0.70* |
| Serial3Responses | 1.98 | **0.07** | *-0.57* | *0.71* | 3.26 | **0.12** | *-0.52* | *0.76* | 0.39 | **0.01** | *-0.63* | *0.66* | 1.45 | **0.06** | *-0.58* | *0.70* |
| Serial3Errors | 0.06 | **0.02** | *-0.62* | *0.66* | -1.15 | **-0.29** | *-0.94* | *0.35* | 0.45 | **0.18** | *-0.46* | *0.82* | -0.34 | **-0.10** | *-0.74* | *0.54* |
| Serial7Responses | -0.06 | **0.00** | *-0.64* | *0.64* | -1.22 | **-0.06** | *-0.70* | *0.58* | 0.74 | **0.04** | *-0.60* | *0.68* | 2.27 | **0.12** | *-0.52* | *0.76* |
| Serial7Errors | 0.16 | **0.04** | *-0.60* | *0.68* | -0.11 | **-0.05** | *-0.69* | *0.59* | -0.11 | **-0.04** | *-0.68* | *0.61* | 0.57 | **0.17** | -0.47 | 0.81 |

Table 44. Kennedy et al., (2002): comparing effect sizes between 1 hour and 4 hours

|  | Placebo | | | | Ginkgo | | | | Ginseng | | | | Gink_Gin | | | |
| --- | --- | --- | --- | --- | --- | --- | --- | --- | --- | --- | --- | --- | --- | --- | --- | --- |
|  | Mean | *d* | LCI | UCI | Mean | *d* | LCI | UCI | Mean | *d* | LCI | UCI | Mean | *d* | LCI | UCI |
| Quality of Memory | -22.88 | **-0.33** | *-0.98* | *0.31* | -27.56 | **-0.38** | *-1.02* | *0.27* | 6.70 | **0.10** | *-0.54* | *0.74* | -30.73 | **-0.35** | *-0.99* | *0.30* |
| Secondary Memory | -15.42 | **-0.28** | *-0.92* | *0.36* | -23.42 | **-0.37** | *-1.01* | *0.28* | 9.91 | **0.18** | *-0.46* | *0.82* | -22.79 | **-0.38** | *-1.02* | *0.27* |
| Working Memory | -7.46 | **-0.34** | *-0.98* | *0.31* | -4.14 | **-0.17** | *-0.82* | *0.47* | -3.21 | **-0.19** | *-0.83* | *0.45* | -7.94 | **-0.23** | *-0.87* | *0.41* |
| Speed of Memory | 42.05 | **0.11** | *-0.54* | *0.75* | 66.88 | **0.18** | *-0.47* | *0.82* | -23.26 | **-0.06** | *-0.70* | *0.58* | -0.69 | **0.00** | *-0.64* | *0.64* |
| Quality of Attention | -0.05 | **-0.02** | *-0.66* | *0.62* | -1.45 | **-0.34** | *-0.99* | *0.30* | 0.25 | **0.05** | *-0.59* | *0.70* | -0.85 | **-0.23** | *-0.87* | *0.41* |
| Speed of Attention | 5.11 | **0.04** | *-0.60* | *0.68* | 38.92 | **0.28** | *-0.36* | *0.93* | 33.52 | **0.26** | *-0.38* | *0.91* | 28.80 | **0.01** | *-0.63* | *0.65* |
| Alert | -1.53 | **-0.09** | *-0.73* | *0.55* | -0.02 | **0.00** | *-0.64* | *0.64* | 0.75 | **0.04** | *-0.60* | *0.68* | 1.91 | **0.11** | *-0.53* | *0.75* |
| Content | -1.80 | **-0.13** | *-0.77* | *0.51* | -0.24 | **-0.01** | *-0.65* | *0.63* | -0.88 | **-0.06** | *-0.70* | *0.58* | 1.06 | **0.06** | *-0.58* | *0.70* |
| Calm | -3.58 | **-0.22** | *-0.86* | *0.42* | -2.33 | **-0.17** | *-0.81* | *0.47* | 2.56 | **0.13** | *-0.51* | *0.77* | -1.58 | **-0.11** | *-0.75* | *0.54* |
| Serial3Responses | -1.22 | **-0.04** | *-0.68* | *0.60* | 2.95 | **0.11** | *-0.54* | *0.75* | -2.28 | **-0.09** | *-0.73* | *0.55* | 1.95 | **0.07** | *-0.57* | *0.72* |
| Serial3Errors | 1.39 | **0.38** | *-0.26* | *1.03* | -1.77 | **-0.45** | *-1.10* | *0.20* | 2.22 | **0.90** | *0.23* | *1.57* | 1.50 | **0.45** | *-0.20* | *1.10* |
| Serial7Responses | -1.90 | **-0.10** | *-0.74* | *0.54* | -0.90 | **-0.04** | *-0.68* | *0.60* | 0.16 | **0.01** | *-0.63* | *0.65* | 2.85 | **0.15** | *-0.49* | *0.79* |
| Serial7Errors | 1.31 | **0.37** | *-0.28* | *1.01* | -0.58 | **-0.24** | *-0.88* | *0.40* | -0.16 | **-0.05** | *-0.69* | *0.59* | 0.63 | **0.19** | *-0.45* | *0.83* |

Table 45. Kennedy et al., (2002): comparing effect sizes between 1 hour and 6 hours

|  | Placebo | | | | Ginkgo | | | | Ginseng | | | | Gink_Gin | | | |
| --- | --- | --- | --- | --- | --- | --- | --- | --- | --- | --- | --- | --- | --- | --- | --- | --- |
|  | Mean | *d* | LCI | UCI | Mean | *d* | LCI | UCI | Mean | *d* | LCI | UCI | Mean | *d* | LCI | UCI |
| Quality of Memory | -17.29 | **-0.25** | *-0.90* | *0.39* | -6.50 | **-0.09** | *-0.73* | *0.55* | 3.76 | **0.06** | *-0.58* | *0.70* | -48.81 | **-0.55** | *-1.21* | *0.10* |
| Secondary Memory | -6.17 | **-0.11** | *-0.75* | *0.53* | -1.83 | **-0.03** | *-0.67* | *0.61* | 12.00 | **0.22** | *-0.43* | *0.86* | -40.29 | **-0.66** | *-1.32* | *-0.01* |
| Working Memory | -11.13 | **-0.50** | *-1.15* | *0.15* | -4.67 | **-0.20** | *-0.84* | *0.45* | -8.24 | **-0.48** | *-1.13* | *0.17* | -8.52 | **-0.25** | *-0.89* | *0.39* |
| Speed of Memory | -52.86 | **-0.13** | *-0.77* | *0.51* | -31.00 | **-0.08** | *-0.72* | *0.56* | -33.51 | **-0.09** | *-0.73* | *0.56* | -35.69 | **-0.09** | *-0.73* | *0.55* |
| Quality of Attention | 0.75 | **0.23** | *-0.41* | *0.88* | -1.15 | **-0.27** | *-0.92* | *0.37* | 0.35 | **0.08** | *-0.56* | *0.72* | 0.35 | **0.10** | *-0.55* | *0.74* |
| Speed of Attention | 24.89 | **0.19** | *-0.45* | *0.84* | 16.31 | **0.12** | *-0.52* | *0.76* | 18.35 | **0.14** | *-0.50* | *0.78* | 5.27 | **0.00** | *-0.64* | *0.64* |
| Alert | -1.33 | **-0.08** | *-0.72* | *0.56* | 1.39 | **0.08** | *-0.56* | *0.72* | -1.94 | **-0.11** | *-0.75* | *0.53* | 1.10 | **0.06** | *-0.58* | *0.70* |
| Content | -3.25 | **-0.24** | *-0.88* | *0.41* | -2.05 | **-0.12** | *-0.76* | *0.52* | -2.31 | **-0.16** | *-0.81* | *0.48* | 1.56 | **0.09** | *-0.55* | *0.73* |
| Calm | -0.15 | **-0.01** | *-0.65* | *0.63* | -7.88 | **-0.57** | *-1.22* | *0.08* | 1.43 | **0.07** | *-0.57* | *0.71* | -1.78 | **-0.12** | *-0.76* | *0.52* |
| Serial3Responses | -2.72 | **-0.10** | *-0.74* | *0.54* | 2.45 | **0.09** | *-0.55* | *0.73* | 0.33 | **0.01** | *-0.63* | *0.65* | 3.56 | **0.14** | *-0.50* | *0.78* |
| Serial3Errors | 1.06 | **0.29** | *-0.35* | *0.94* | -1.66 | **-0.42** | *-1.07* | *0.23* | 1.72 | **0.70** | *0.04* | *1.36* | -0.06 | **-0.02** | *-0.66* | *0.62* |
| Serial7Responses | -1.21 | **-0.07** | *-0.71* | *0.57* | 1.26 | **0.06** | *-0.58* | *0.70* | 1.11 | **0.06** | *-0.58* | *0.70* | 0.58 | **0.03** | *-0.61* | *0.67* |
| Serial7Errors | 1.37 | **0.38** | *-0.26* | *1.03* | 1.05 | **0.43** | *-0.21* | *1.08* | 0.00 | **0.00** | *-0.64* | *0.64* | -0.58 | **-0.18** | *-0.82* | *0.47* |

Table 46. Kennedy et al., (2002): comparing effect sizes between 2.5 and 4 hours

|  | Placebo | | | | Ginkgo | | | | Ginseng | | | | Gink_Gin | | | |
| --- | --- | --- | --- | --- | --- | --- | --- | --- | --- | --- | --- | --- | --- | --- | --- | --- |
|  | Mean | *d* | LCI | UCI | Mean | *d* | LCI | UCI | Mean | *d* | LCI | UCI | Mean | *d* | LCI | UCI |
| Quality of Memory | -8.37 | **-0.12** | *-0.76* | *0.52* | -15.67 | **-0.21** | *-0.86* | *0.43* | 3.42 | **0.05** | *-0.59* | *0.69* | -23.08 | **-0.26** | *-0.90* | *0.38* |
| Secondary Memory | -5.71 | **-0.10** | *-0.74* | *0.54* | -9.94 | **-0.16** | *-0.80* | *0.49* | 8.91 | **0.16** | *-0.48* | *0.80* | -23.50 | **-0.39** | *-1.03* | *0.26* |
| Working Memory | -2.65 | **-0.12** | *-0.76* | *0.52* | -5.73 | **-0.24** | *-0.88* | *0.40* | -5.49 | **-0.32** | *-0.96* | *0.32* | 0.42 | **0.01** | *-0.63* | *0.65* |
| Speed of Memory | 19.51 | **0.05** | *-0.59* | *0.69* | 87.33 | **0.23** | *-0.41* | *0.87* | 6.51 | **0.02** | *-0.62* | *0.66* | -16.20 | **-0.04** | *-0.68* | *0.60* |
| Quality of Attention | 0.55 | **0.17** | *-0.47* | *0.81* | -1.25 | **-0.30** | *-0.94* | *0.35* | -0.40 | **-0.09** | *-0.73* | *0.55* | -0.50 | **-0.14** | *-0.78* | *0.50* |
| Speed of Attention | 8.71 | **0.07** | *-0.57* | *0.71* | 20.37 | **0.15** | *-0.49* | *0.79* | 29.01 | **0.23** | *-0.41* | *0.87* | 32.70 | **0.01** | *-0.63* | *0.65* |
| Alert | -0.81 | **-0.05** | *-0.69* | *0.59* | 0.65 | **0.04** | *-0.60* | *0.68* | 0.37 | **0.02** | *-0.62* | *0.66* | 2.82 | **0.16** | *-0.48* | *0.80* |
| Content | 0.38 | **0.03** | *-0.61* | *0.67* | 4.02 | **0.24** | *-0.41* | *0.88* | -0.56 | **-0.04** | *-0.68* | *0.60* | -0.83 | **-0.05** | *-0.69* | *0.59* |
| Calm | -1.12 | **-0.07** | *-0.71* | *0.57* | 0.92 | **0.07** | *-0.57* | *0.71* | 3.88 | **0.20** | *-0.44* | *0.84* | -2.43 | **-0.16** | *-0.80* | *0.48* |
| Serial3Responses | -3.20 | **-0.12** | *-0.76* | *0.52* | -0.31 | **-0.01** | *-0.65* | *0.63* | -2.67 | **-0.10** | *-0.74* | *0.54* | 0.50 | **0.02** | *-0.62* | *0.66* |
| Serial3Errors | 1.33 | **0.37** | *-0.28* | *1.01* | -0.62 | **-0.16** | *-0.80* | *0.48* | 1.77 | **0.72** | *0.06* | *1.38* | 1.84 | **0.55** | *-0.10* | *1.20* |
| Serial7Responses | -1.84 | **-0.10** | *-0.74* | *0.54* | 0.32 | **0.01** | *-0.63* | *0.66* | -0.58 | **-0.03** | *-0.67* | *0.61* | 0.58 | **0.03** | *-0.61* | *0.67* |
| Serial7Errors | 1.15 | **0.32** | *-0.32* | *0.97* | -0.47 | **-0.19** | *-0.84* | *0.45* | -0.05 | **-0.02** | *-0.66* | *0.62* | 0.06 | **0.02** | *-0.62* | *0.66* |

Table 47. Kennedy et al., (2002): comparing effect sizes between 2.5 and 6 hours

|  | Placebo | | | | Ginkgo | | | | Ginseng | | | | Gink_Gin | | | |
| --- | --- | --- | --- | --- | --- | --- | --- | --- | --- | --- | --- | --- | --- | --- | --- | --- |
|  | Mean | *d* | LCI | UCI | Mean | *d* | LCI | UCI | Mean | *d* | LCI | UCI | Mean | *d* | LCI | UCI |
| Quality of Memory | -2.78 | **-0.04** | *-0.68* | *0.60* | 5.39 | **0.07** | *-0.57* | *0.71* | 0.48 | **0.01** | *-0.63* | *0.65* | -41.16 | **-0.47** | *-1.12* | *0.18* |
| Secondary Memory | 3.54 | **0.06** | *-0.58* | *0.70* | 11.65 | **0.18** | *-0.46* | *0.82* | 11.00 | **0.20** | *-0.44* | *0.84* | -41.00 | **-0.68** | *-1.33* | *-0.02* |
| Working Memory | -6.32 | **-0.29** | *-0.93* | *0.36* | -6.26 | **-0.26** | *-0.91* | *0.38* | -10.52 | **-0.61** | *-1.27* | *0.04* | -0.16 | **0.00** | *-0.64* | *0.64* |
| Speed of Memory | -75.40 | **-0.19** | *-0.83* | *0.45* | -10.55 | **-0.03** | *-0.67* | *0.61* | -3.74 | **-0.01** | *-0.65* | *0.63* | -51.20 | **-0.13** | *-0.77* | *0.51* |
| Quality of Attention | 1.35 | **0.42** | *-0.23* | *1.07* | -0.95 | **-0.23** | *-0.87* | *0.42* | -0.30 | **-0.07** | *-0.71* | *0.57* | 0.70 | **0.19** | *-0.45* | *0.83* |
| Speed of Attention | 28.49 | **0.22** | *-0.42* | *0.86* | -2.24 | **-0.02** | *-0.66* | *0.62* | 13.84 | **0.11** | *-0.53* | *0.75* | 9.17 | **0.00** | *-0.64* | *0.64* |
| Alert | -0.61 | **-0.04** | *-0.68* | *0.60* | 2.06 | **0.12** | *-0.52* | *0.76* | -2.32 | **-0.13** | *-0.77* | *0.51* | 2.01 | **0.12** | *-0.52* | *0.76* |
| Content | -1.07 | **-0.08** | *-0.72* | *0.56* | 2.21 | **0.13** | *-0.51* | *0.77* | -1.99 | **-0.14** | *-0.78* | *0.50* | -0.33 | **-0.02** | *-0.66* | *0.62* |
| Calm | 2.31 | **0.14** | *-0.50* | *0.78* | -4.63 | **-0.33** | *-0.98* | *0.31* | 2.75 | **0.14** | *-0.50* | *0.78* | -2.63 | **-0.18** | *-0.82* | *0.47* |
| Serial3Responses | -4.70 | **-0.17** | *-0.81* | *0.47* | -0.81 | **-0.03** | *-0.67* | *0.61* | -0.06 | **0.00** | *-0.64* | *0.64* | 2.11 | **0.08** | *-0.56* | *0.72* |
| Serial3Errors | 1.00 | **0.28** | *-0.37* | *0.92* | -0.51 | **-0.13** | *-0.77* | *0.51* | 1.27 | **0.52** | *-0.13* | *1.17* | 0.28 | **0.08** | *-0.56* | *0.72* |
| Serial7Responses | -1.15 | **-0.06** | *-0.70* | *0.58* | 2.48 | **0.12** | *-0.53* | *0.76* | 0.37 | **0.02** | *-0.62* | *0.66* | -1.69 | **-0.09** | *-0.73* | *0.55* |
| Serial7Errors | 1.21 | **0.34** | *-0.31* | *0.98* | 1.16 | **0.48** | *-0.17* | *1.13* | 0.11 | **0.04** | *-0.61* | *0.68* | -1.15 | **-0.35** | *-0.99* | *0.30* |

Table 48. Kennedy et al., (2002): comparing effect sizes between 4 hours and 6 hours

|  | Placebo | | | | Ginkgo | | | | Ginseng | | | | Gink_Gin | | | |
| --- | --- | --- | --- | --- | --- | --- | --- | --- | --- | --- | --- | --- | --- | --- | --- | --- |
|  | Mean | *d* | LCI | UCI | Mean | *d* | LCI | UCI | Mean | *d* | LCI | UCI | Mean | *d* | LCI | UCI |
| Quality of Memory | 5.59 | **0.08** | *-0.56* | *0.72* | 21.06 | **0.29** | *-0.36* | *0.93* | -2.94 | **-0.04** | *-0.68* | *0.60* | -18.08 | **-0.21** | *-0.85* | *0.44* |
| Secondary Memory | 9.25 | **0.17** | *-0.47* | *0.81* | 21.59 | **0.34** | *-0.31* | *0.98* | 2.09 | **0.04** | *-0.60* | *0.68* | -17.50 | **-0.29** | *-0.93* | *0.36* |
| Working Memory | -3.67 | **-0.17** | *-0.81* | *0.48* | -0.53 | **-0.02** | *-0.66* | *0.62* | -5.03 | **-0.29** | *-0.94* | *0.35* | -0.58 | **-0.02** | *-0.66* | *0.62* |
| Speed of Memory | -94.91 | **-0.24** | *-0.88* | *0.41* | -97.88 | **-0.26** | *-0.90* | *0.39* | -10.25 | **-0.03** | *-0.67* | *0.61* | -35.00 | **-0.09** | *-0.73* | *0.55* |
| Quality of Attention | 0.80 | **0.25** | *-0.39* | *0.89* | 0.30 | **0.07** | *-0.57* | *0.71* | 0.10 | **0.02** | *-0.62* | *0.66* | 1.20 | **0.33** | *-0.32* | *0.97* |
| Speed of Attention | 19.78 | **0.15** | *-0.49* | *0.80* | -22.61 | **-0.17** | *-0.81* | *0.48* | -15.17 | **-0.12** | *-0.76* | *0.52* | -23.53 | **-0.01** | *-0.65* | *0.64* |
| Alert | 0.20 | **0.01** | *-0.63* | *0.65* | 1.41 | **0.08** | *-0.56* | *0.72* | -2.69 | **-0.15** | *-0.79* | *0.49* | -0.81 | **-0.05** | *-0.69* | *0.59* |
| Content | -1.45 | **-0.11** | *-0.75* | *0.54* | -1.81 | **-0.11** | *-0.75* | *0.53* | -1.43 | **-0.10** | *-0.74* | *0.54* | 0.50 | **0.03** | *-0.61* | *0.67* |
| Calm | 3.43 | **0.21** | *-0.43* | *0.85* | -5.55 | **-0.40** | *-1.05* | *0.25* | -1.13 | **-0.06** | *-0.70* | *0.58* | -0.20 | **-0.01** | *-0.65* | *0.63* |
| Serial3Responses | -1.50 | **-0.05** | *-0.70* | *0.59* | -0.50 | **-0.02** | *-0.66* | *0.62* | 2.61 | **0.10** | *-0.54* | *0.74* | 1.61 | **0.06** | *-0.58* | *0.70* |
| Serial3Errors | -0.33 | **-0.09** | *-0.73* | *0.55* | 0.11 | **0.03** | *-0.61* | *0.67* | -0.50 | **-0.20** | *-0.85* | *0.44* | -1.56 | **-0.47** | *-1.11* | *0.18* |
| Serial7Responses | 0.69 | **0.04** | *-0.60* | *0.68* | 2.16 | **0.10** | *-0.54* | *0.74* | 0.95 | **0.05** | *-0.59* | *0.69* | -2.27 | **-0.12** | *-0.76* | *0.52* |
| Serial7Errors | 0.06 | **0.02** | *-0.62* | *0.66* | 1.63 | **0.67** | *0.02* | *1.33* | 0.16 | **0.05** | *-0.59* | *0.69* | -1.21 | **-0.37** | *-1.01* | *0.28* |
