# Supplementary material for: A systematic review of research investigating the physiological and psychological effects of combining Ginkgo biloba and Panax ginseng into a single treatment in humans: Implications for research design and analysis
Source: Brain Behav. 2019 Feb 6;9(3):e01217. doi: 10.1002/brb3.1217 (PMC6422825; doi:10.1002/brb3.1217)
Supplement: Supplementary file 2 [file BRB3-9-e01217-s002.docx]

Appendix 1: Summary of papers investigating the physiological and psychological effect of a *Panax ginseng* / *Ginkgo biloba* combination treatment. All studies investigated a proprietary product – Gincosan^®^. All results report combination vs placebo and relate to statistical significance results only. * denotes a primary outcome measure defined by authors in their paper. ** denotes a secondary outcome defined by authors in their paper. T denotes treatment; P denotes placebo; DB denotes double blind; PC denotes placebo control; RM denotes repeated measures; R denotes randomised; BS denotes between subjects.

| **Authors/Aim** | **Participants** | **Design** | **Treatment** | **Behavioural Concept** | **Measurement Tool** | **Results Behavioural** | **Physiological Measurements** | **Results Physiological** |
| --- | --- | --- | --- | --- | --- | --- | --- | --- |
| Kiesewetter et al. (1992)  Test the hemorrheological and circulatory effects of Gincosan in an acute trial | N= 10 (8 male)  Mean age 25.7 SD 4.4yrs  All suffered at least one pathological abnormality:  1. plasma  viscosity   > 1.29mPas  2. erythrocyte   aggregation  > 17.3  3. erythrocyte  rigidity   > 1.14 4. constantly  increased  platelet   aggregation | Study 1 An open label study. Efficacy measured at 1hr, 2hr and 3 hr after dose  Study 2 DB, PC, RM design. Efficacy measured at 1hr after dose  Study 2: omnibus test: Friedman Pairwise comparisons: Wilcoxon  Significance level: 0.05 ‘Trend level’: 0.05 < *p* < 0.10  Effect size not reported | Study 1 Gincosan  Study 2 Placebo  Gincosan  Study 1 320 mg   Study 2 160 mg 320 mg  Treatment duration:  single dose |  |  |  | Blood Pressure | T (160 mg) ↑ BP (reduced systolic BP) at 1hr after dose T (320 mg) ↑ BP (reduced systolic BP and reduced diastolic BP) at 1hr after dose |
|  |  |  |  |  |  |  | Heart Rate | T (320 mg) ↑ HR (reduced HR) at 1hr after dose |
|  |  |  |  |  |  |  | Haematocrit |  |
|  |  |  |  |  |  |  | Plasma Viscosity |  |
|  |  |  |  |  |  |  | Erythrocyte Aggregation |  |
|  |  |  |  |  |  |  | Erythrocyte Rigidity |  |
|  |  |  |  |  |  |  | Spontaneous Platelet  Aggregation | T (160 mg) ↑ platelet aggregation (reduced spontaneous aggregation) at 1hr after dose T (320 mg) ↑ platelet aggregation (reduced spontaneous aggregation) at 1hr after dose |
|  |  |  |  |  |  |  | Cutaneous Erythrocyte  Velocity in capillaries | T (320 mg) ↑ velocity (increased velocity) at 1hr after dose |
|  |  |  |  |  |  |  | Capillary Diameter |  |
| Kwiecinski et al. (1997)  Investigate the efficacy and safety of Gincosan in the treatment of (chronic) cerebrovascular disorders (CCDs) | N= 85 enrolled N = 72 completed   n(T) = 35 Mean age 57.5 Range 43-72  n(P) = 37 Mean age 57 Range 44-70   Participants display at least one symptom of cerebrovascular disorders | DB,R,PC,BS.  12 week trial (4wks placebo run-in and 8 weeks of treatment v placebo.) adverse event measured at 0,2,4,6,8 wks. Efficacy measured at minus4,0,4 and 8wks. TCD measured at minus4,0 and 8wks) Chi square test for improvement of symptoms  Wilcoxon signed rank test for repeated measurements within treatments  Rank sum test for comparisons between groups  Significance level: 0.05  Effect size not reported | Placebo  Gincosan   160 mg b.i.d.  Treatment duration :  8 weeks | Dizziness | Concepts assessed by clinician clinical assessment of symptom severity (3=severe; 2 = moderate; 1 = slight; 0=absent) |  | Mean flow velocity LMCA | T (160 mg) ↑ Mean blood flow velocity in the Middle Cerebral Artery |
|  |  |  |  | Tinnitus |  |  |  |  |
|  |  |  |  | Headache |  |  |  |  |
|  |  |  |  | Day time irritability |  |  | Wave amplitude LMCA |  |
|  |  |  |  | Night-time restlessness |  |  | Pulsatility LMCA |  |
|  |  |  |  | Lack of concentration |  | T ↑ concentration (% of patients showing improvements) week 8 only |  |  |
|  |  |  |  | Forgetfulness |  |  |  |  |
|  |  |  |  | Depressive mood |  |  |  |  |
|  |  |  |  | Feelings of rejection |  |  |  |  |
|  |  |  |  | Narrowed interest |  |  |  |  |
|  |  |  |  | Social withdrawal |  |  |  |  |
|  |  |  |  | Memory | Digit span |  |  |  |
|  |  |  |  | Memory | Number recall |  |  |  |
|  |  |  |  | Visual Scanning  (concentration) | Letter cancellation | T (dose mg) ↑ letter cancellation (concentration) |  |  |
| Wesnes et al. (1997)  Primary purpose’: “explore the potential of Gincosan to alter the quality of cognitive function” (p. 677) | N=64 (20 male) Range 40-65 yrs Mean age 54.7 SD 5.9   All participants reported neurasthenic complaints identified using SCL-90-R questionnaire and the ICD-10 F48.0 diagnostic guidelines | DB,PC,BS. 90 Day trial. Efficacy measured at day 0 (baseline) and after dose at day 1, day 30 and day 90. Testing was conducted 1 hour after a morning dose and 1 hour after an afternoon dose. Adverse event were measured. Primary end points were defined at 1hr after the morning dose on day 90 for 4 of the outcome measures | Placebo  Gincosan   1. 80 mg b.i.d. (30 mg of GB and 50 mg of PG)  2. 160 mg b.i.d. (60 mg of GB and 100 mg of PG)  3. 320 mg b.i.d. (120 mg of GB and 200 mg of PG)   Treatment duration: 90 days  3 (study day)-by-4 (dose) ANOVA. N.b.: baseline scores subtracted from scores on Days 1, 30 and 90  Planned comparisons between placebo and (other) doses  Significance level: 0.05  Effect size not reported | Memory | Immediate Word Recall |  | Heart rate * Heart Rate at Maximum exercise load (8 minute cycle) HR measured at 2,4,6 and 8 minutes | T(80 mg) ↑ HR (reduced HR) on D90 at maximum load 1hr after morning |
|  |  |  |  | Memory | Delayed Picture Recognition |  |  |  |
|  |  |  |  | Memory | Numeric Working Memory |  |  |  |
|  |  |  |  | Memory | Delayed Word Recall |  |  |  |
|  |  |  |  | Memory | Delayed word recognition |  |  |  |
|  |  |  |  | Memory* | quality-of-memory index  (accuracy) | T (320 mg)↑on D1, D30 and D90 1 hr after morning dose  T (160 mg)↑on D1, D30 1 hr after morning dose T (80 mg) ↑on D1, D30 1 hr after morning dose  T (320 mg)↓ on D1 and D90 1hr after afternoon dose  T (160 mg)↓ on D90 1hr after afternoon dose |  |  |
|  |  |  |  | Memory* | quality-of-memory index  (speed RT) | T (160 mg)↑ (quicker) on D1, D30 and D90 1 hr after morning dose T (80 mg, 160 mg and 320 mg) ↓ (slower) on D1 1hr after afternoon dose |  |  |
|  |  |  |  | Attention | Simple Reaction Time |  |  |  |
|  |  |  |  | Attention | Choice Reaction Time |  |  |  |
|  |  |  |  | Attention | Digit vigilance test |  |  |  |
|  |  |  |  | Multiple constructs* | Vienna Determination Test | No effect on primary end point (No of correct responses)  T (80 mg) ↑ (No of correct reactions in time) 1hr after afternoon dose D90  T (160 mg and 320 mg) ↑ No of correct reactions in time) 1hr after morning dose D90 |  |  |
|  |  |  |  | Alertness | Bond Lader Mood Scale |  |  |  |
|  |  |  |  | Calmness | Bond Lader Mood Scale |  |  |  |
|  |  |  |  | Content | Bond Lader Mood Scale |  |  |  |
|  |  |  |  | Neurasthenic Symptoms | SCL-90-R Symptom Checklist | no effect on any individual symptoms T (320 mg) ↑ totalSCL-90-R score (greater fall in the total score) |  |  |
| Wesnes et al. (2000)  Aim: “identify [1] whether the combination has the beneficial effects on memory function seen in the previous study in middle-aged healthy individuals and [2] also to identify whether any evidence of a biphasic effect could be seen” (p. 354). | N=256 (93male) Range 38 - 66 yrs Mean age 56.07  SD 6.87) recruited from 7 health centres  (5UK; 2 Swenden)  Healthy Volunteers Only | Multi centred trial utilising DB,PC,BS. 16 week trial. 2 week placebo run-in. 12 week treatment phase. 2 week washout period. Efficacy measured at week 0 (baseline) and after dose at week 4, week 8, week 12 and week 14. testing conducted -1hr predose and 1hr, 3hrs and 6hrs after dose on each visit day. Adverse events measured.  Cognitive tasks and mood (Bond-Lader VAS scale): 2 ([dosing] regimen: 160 or 320 mg)-by-2 ([dosing] condition: placebo, verum)-by-(4) (week: 4, 8, 12, 14)-by-(4) (time of day: 1 hrs before dose, 1/3/6 hrs after dose) ANOVA. N.b.: baseline scores subtracted from scores on Weeks 4, 8, 12 and 14)  Questionnaire data: 2 ([dosing] regimen: 160 or 320 mg)-by-2 ([dosing] condition: placebo, verum)-by-(4) (week: 4, 8, 12, 14) ANOVA  Significance level not reported  Effect size not reported | Placebo Gincosan  1. 160 mg b.i.d. (60 mg GB, 100 mg PG)   2. 320 mg morning dose  Treatment duration:12weeks | Memory* | quality-of-memory index | ANOVA revealed significant main effect of treatment condition  T = ↑ quality of memory index  Authors did not explore main effect further with post-hoc. |  |  |
|  |  |  |  | Memory | Quality of working memory factor |  |  |  |
|  |  |  |  | Memory | Quality of episodic secondary memory factor |  |  |  |
|  |  |  |  | Memory** | Speed of Memory process factor |  |  |  |
|  |  |  |  | Attention ** | Power of attention factor |  |  |  |
|  |  |  |  | Attention ** | Continuity of attention factor |  |  |  |
|  |  |  |  | Memory | immediate word recall |  |  |  |
|  |  |  |  | Memory | Spatial Working Memory |  |  |  |
|  |  |  |  | Memory | Numeric Working Memory |  |  |  |
|  |  |  |  | Memory | Delayed Word recall |  |  |  |
|  |  |  |  | Memory | Word recognition |  |  |  |
|  |  |  |  | Memory | Picture Recognition |  |  |  |
|  |  |  |  | Attention | Simple Reaction Time |  |  |  |
|  |  |  |  | Attention | Choice Reaction time |  |  |  |
|  |  |  |  | Attention | Digit vigilance Task |  |  |  |
|  |  |  |  | Attention | Joystick tracking task |  |  |  |
|  |  |  |  | Alert | Bond Lader Mood Scale |  |  |  |
|  |  |  |  | Calm | Bond Lader Mood Scale | higher order interaction: not-interpretable |  |  |
|  |  |  |  | Content | Bond Lader Mood Scale |  |  |  |
|  |  |  |  | Anger | Profile of Mood States |  |  |  |
|  |  |  |  | Confusion | Profile of Mood States |  |  |  |
|  |  |  |  | Depression | Profile of Mood States |  |  |  |
|  |  |  |  | Fatigue | Profile of Mood States |  |  |  |
|  |  |  |  | Tension | Profile of Mood States |  |  |  |
|  |  |  |  | Vigour | Profile of Mood States |  |  |  |
|  |  |  |  | General Well-being | General Well-being Schedule |  |  |  |
|  |  |  |  | Psychiatric Well-being | The Hopkins Symptom Checklist |  |  |  |
|  |  |  |  | Sleep Quality | St Mary's Hospital Sleep  Questionnaire |  |  |  |
| Kennedy et al. (2001)  Aim: investigate the effects of acute administration of Gincosan on cognitive performance | 20 (10 male) Mean age 20.6yrs  SD 4.2yrs  Healthy Young Volunteers Only | Single-centre trial utilising PC, DB, B, CO design. single acute ingestion (each ingestion was spaced with a 7 day washout period). Efficacy measured at baseline and after dose at 1hr, 2.5hrs, 4hrs and 6hrs after dose on each visit day. Adverse events were measured. | Placebo Gincosan  Three Treatment Doses  1. 320 mg (2 capsules)  2. 640 mg (4 capsules)  3. 960 mg (6 capsules)   Each capsule contained 60 mg GB and 100 mg PG  treatment duration: one single acute dose taken in the morning following an overnight fast  *t*-tests for planned comparisons of placebo with the verum conditions with MSE from ANOVA  Effect size not reported | Memory* | quality-of-memory factor | T (960 mg) ↑ performance at 1hr and 6hrs after dose |  |  |
|  |  |  |  | Memory* | Secondary memory sub-factor | T (960 mg) ↑ performance at 1hr and 6hrs after dose |  |  |
|  |  |  |  | Memory* | Working memory sub-factor |  |  |  |
|  |  |  |  | Memory* | Speed of memory factor |  |  |  |
|  |  |  |  | Attention* | Speed of attention factor | T (320 mg) ↓ performance at 4h and 6h after dose T (640 mg) ↓performance at 4h after dose |  |  |
|  |  |  |  | Attention* | Accuracy of Attention Factor |  |  |  |
|  |  |  |  | Memory | Immediate Word recall | T (320 mg) ↑ performance at 1h, 4h and 6h after dose T (640 mg) ↑ performance at 2.5h, 4h and 6h after dose T (960 mg) ↑performance at 1h, 4h and 6hr after dose |  |  |
|  |  |  |  | Memory | spatial working memory | T (640 mg) ↑ performance at 1hr after dose |  |  |
|  |  |  |  | Memory | numeric working memory | T (960 mg) ↑ performance of the speed of this task at 4h after dose |  |  |
|  |  |  |  | Memory | delayed word recall | T (320 mg) ↑ performance at 4h and 6h after dose T (960 mg) ↑ performance at 6h after dose |  |  |
|  |  |  |  | Memory | delayed word recognition |  |  |  |
|  |  |  |  | Memory | delayed picture recognition | T (960 mg) ↑ performance at 4h and 6h after dose T (320 mg and 640 mg) ↓performance at 1h |  |  |
|  |  |  |  | Attention | simple reaction time | T (640 mg) ↓ performance at 4h after dose |  |  |
|  |  |  |  | Attention | digit vigilance task | T (320 mg) ↓ performance at 1, 2.5 4h and 6h after dose T (640 mg) ↓ performance at 4h after dose |  |  |
|  |  |  |  | Attention | choice reaction time | T (640 mg) ↑ performance (speed) at 2.5h and 6h after dose T (320 mg)↓ performance (accuracy) at 2.5h and 6h T (960 mg) ↓performance (accuracy) at 2.5h after dose |  |  |
|  |  |  |  | Alert | Bond Lader mood Scale |  |  |  |
|  |  |  |  | Calm | Bond Lader mood Scale |  |  |  |
|  |  |  |  | Content | Bond Lader mood Scale |  |  |  |
| Kennedy et al. (2002)  Aim (implicit): directly investigate the comparative cognitive effects of a single dose of Ginkgo biloba, ginseng and Gincosan | 20 (5 male)  mean age = 21.2  SD = 3.9  Healthy Young Volunteers Only | Single-centre trial utilising PC, DB, B, CO design. single acute ingestion (each ingestion was spaced with a 7 day washout period). Efficacy measured at baseline and after dose at 1hr, 2.5hrs, 4hrs and 6hrs after dose on each visit day. Adverse events were measured. | Placebo  Ginkgo biloba Ginseng  Gincosan  Each participant ingested 6 capsules at each visit  Three treatment doses  1. 360 mg Ginkgo  2. 400 mg Ginseng  3. 960 mg Gincosan  one single acute dose taken in the morning following an overnight fast  *t*-tests for planned comparisons of placebo with the verum conditions with MSE from ANOVA  Effect size not reported | Memory* | quality-of-memory factor | T (960 mg) ↑ performance at 1hr, 2.5hrs and 4hrs after dose |  |  |
|  |  |  |  | Memory* | Secondary memory sub-factor | T (960 mg) ↑ performance at 1hr and 2.5hr after dose |  |  |
|  |  |  |  | Memory* | Working memory sub-factor | T (960 mg) ↑ performance at 1hr and 6hrs after dose |  |  |
|  |  |  |  | Memory* | Speed of memory factor |  |  |  |
|  |  |  |  | Attention* | Speed of attention factor | T (960 mg) ↓ performance at 4hrs after dose |  |  |
|  |  |  |  | Attention* | Accuracy of Attention Factor |  |  |  |
|  |  |  |  | Memory | Immediate Word recall | T (960 mg) ↑ performance at 4hrs after dose |  |  |
|  |  |  |  | Memory | spatial working memory | T (960 mg) ↑ performance (accuracy) at 4hr and 6hrs after dose  T (960 mg) no effect of performance (speed) |  |  |
|  |  |  |  | Memory | numeric working memory |  |  |  |
|  |  |  |  | Memory | delayed word recall | T (960 mg) ↑ performance at 1hr, 2.5hr and 4hrs after dose |  |  |
|  |  |  |  | Memory | delayed word recognition |  |  |  |
|  |  |  |  | Memory | delayed picture recognition | T (960 mg) ↓ performance at 2.5hrs after dose |  |  |
|  |  |  |  | Attention | simple reaction time | T (960 mg) ↓ performance at 4hrs after dose |  |  |
|  |  |  |  | Attention | digit vigilance task |  |  |  |
|  |  |  |  | Attention | choice reaction time |  |  |  |
|  |  |  |  | Alert | Bond Lader mood Scale |  |  |  |
|  |  |  |  | Calm | Bond Lader mood Scale |  |  |  |
|  |  |  |  | Content | Bond Lader mood Scale | T (960 mg) ↑ content ratings at 2.5hr, 4hrs and 6hrs after dose |  |  |
|  |  |  |  | mental arithmetic | Serial Seven Subtractions | T (960 mg) ↑ performance (number) at 4hrs after dose and ↑ performance (fewer error) at 6hrs after dose |  |  |
|  |  |  |  | mental arithmetic | Serial three Subtractions | T (960 mg) ↑ performance (number) at 6hrs after dose |  |  |
| Scholey et al. (2002)  Aim (implicit): investigate the differential effects of Ginkgo biloba, ginseng and Gincosan on cognitive performance in healthy young adults | Study 1 N=20 (2male)  Mean age 19.9  SD 1.47   Study 2 N=20 (6male)  Mean age 21.3  SD 2.64   Study 3 N=20 (10male)  Mean age 20.6  SD 4.20  Healthy Young Volunteers only | Single-centre trial (reporting 3 studies) all utilising PC, DB, B, CO design. single acute ingestion (each ingestion was spaced with a 7 day washout period). Efficacy measured at baseline and after dose at 1hr, 2.5hrs, 4hrs and 6hrs after dose on each visit day. Adverse events measured | Study 1 Placebo Ginkgo Bilbo Study 2 Placebo  Panax ginseng  Study 3 Placebo Gincosan  Study 1 = 120 mg, 240 mg and 360 mg Ginkgo biloba. Each capsule contained 60 mg GB Study 2 = 200, 400 mg and 600 mg of Panax ginseng. Each capsule contained 100 mg of PG Study 3 = 320 mg, 640 mg and 960 mg of Gincosan. Each capsule contained 60 mg GB and 100 mg PG.  Treatment duration: one single acute dose taken in the morning following an overnight fast  *t*-tests for planned comparisons of placebo with the verum conditions with MSE from ANOVA  Effect size not reported | Mental arithmetic | Serial Three Subtraction  for clarity of focus we report the results of study 3 (Gincosan) in the results column and report the results of study 1 (Ginkgo biloba) and 2 (Panax ginseng) in the footnotes^[[1]](#footnote-1)^ | Study 3 (Gincosan)  T (320 mg) ↑ performance (number) at 4hrs after dose T (640 mg) ↑ performance (fewer errors) at 2.5hrs after dose T (960mb) ↑ performance (fewer errors) at1hr, 2.5hrs, 4hr and 6hr after dose |  |  |
|  |  |  |  | Mental arithmetic | Serial seven subtraction   for clarity of focus we report the results of study 3 (Gincosan) in the results column and report the results of study 1 (Ginkgo biloba) and 2 (Panax ginseng) in the footnote^[[2]](#footnote-2)^ | Study 3 (Gincosan) T (320 mg) ↑ performance (number) at 1hr, 2.5hr, 4hrs and 6hr after dose T (640 mg) ↑ performance (number) at 4hrs after dose  T (320 mg) ↑ performance (fewer error) at 2.5 and 6hrs after dose  T (640 mg) ↑ performance (fewer error) at2.5hr, 4hrs and 6hr after dose T (960 mg) ↑ performance (fewer error) at 2.5hrs and 6 hrs after dose |  |  |
| Harley et al.  (2004)  Investigate the effect of chronic treatment with Gincosan over 12 weeks | N = 70  Range 51-66yrs  13 withdraw  healthy but defined as post-menopausal (not menstruated in past 12 months) | PC, BS design. Efficacy measured at baseline, week 6 and week 12. testing on wk 6 and 12 took place between 2-4hrs after morning dose, with breakfast. | Placebo  Gincosan   320 mg daily  Treatment Duration: 12weeks  2(dose)-by-(3) (study day) ANOVA on cognitive performance  2(dose)-by-(3)(time of testing) MANOVA on memory tests  2(dose)-by-(3)(study day)-by-(2)(time of testing) ANOVA on mood and sleepiness  2(dose)-by-(3)(study day)-by-(2)(time of testing) MANOVA on somatic anxiety  2(dose)-by-(3)(study day)-by-2(menopausal stage) ANOVA on cognitive performance  Significance level not reported  Effect size not reported | Alertness | Bond Lader Mood Scale |  |  |  |
|  |  |  |  | Well-being | Bond Lader Mood Scale |  |  |  |
|  |  |  |  | Anxiety | Bond Lader Mood Scale |  |  |  |
|  |  |  |  | Attention | Paced Serial Addition Test (PASAT) |  |  |  |
|  |  |  |  | Memory | Immediate paragraph recall |  |  |  |
|  |  |  |  | Memory | delayed paragraph recall |  |  |  |
|  |  |  |  | Memory (non-verbal) | Delayed matching to samples test (DMTS) |  |  |  |
|  |  |  |  | Memory | Delayed picture recognition |  |  |  |
|  |  |  |  | Semantic Verbal Fluency | Category generation task |  |  |  |
|  |  |  |  | Mental flexibility | set shifting task |  |  |  |
|  |  |  |  | planning ability | Stockings of Cambridge |  |  |  |
|  |  |  |  | Anxiety and Depression | The Hospital Anxiety and Depression Scale |  |  |  |
|  |  |  |  | Menopausal Symptoms | The Greene Climacteric Scale |  |  |  |
|  |  |  |  | Sleepiness | Stanford Sleepiness Scale and Epworth Sleepiness scale |  |  |  |

1. Serial Threes: Study 1: T (GB 120 mg) ↑ performance (number) at 4hr after dose but ↓ performance (more error) at 4hr after dose. T (GB 240 mg) ↑ performance (number) 4hr and 6hr after dose. T (GB 360 mg) ↑ performance (number) 4hr after dose. Study 2: T (PG) no significant effects. [↑](#footnote-ref-1)
2. Serial Sevens: Study 1 (Ginkgo Bilbo): T (GB 120 mg) ↑ performance (fewer errors) at 2.5hr after dose. T (GB 240 mg) ↑ performance (fewer errors) at 2.5hr after dose. T (GB 360 mg) ↑ performance (fewer errors) at 2.5hr after dose. Study 2 (Panax ginseng): T (PG 200 mg)↓ performance (number) at 1, 2.5 and 6 hrs after dose, ↑ performance (fewer errors) at 4hr after dose; T (PG 400 mg) ↑ performance (fewer error) at 4hrs and 6 hrs after dose. [↑](#footnote-ref-2)
