# Supplementary material for: A systematic review of research investigating the physiological and psychological effects of combining Ginkgo biloba and Panax ginseng into a single treatment in humans: Implications for research design and analysis
Source: Brain Behav. 2019 Feb 6;9(3):e01217. doi: 10.1002/brb3.1217 (PMC6422825; doi:10.1002/brb3.1217)
Supplement: Supplementary file 3 [file BRB3-9-e01217-s003.docx]

Appendix 3: For Kennedy et al., (2001) and (2002), we report in Tables 1 to 6, the standardized effect size (Cohen’s *d*_z_) related to the primary statistical analysis conducted in the original papers (i.e. change from baseline). Each table reports the mean difference (labeled, Mean) between the two treatments of comparison, the *d*_z_ value and the lower and upper confidence interval of the *d*_z_ value, at each time point for each primary outcome measure reported in the original paper. As an example, in Table 3 we can see the mean difference between placebo and 960 mg at the 1 hour time point for the Quality of Memory outcome measure is 40.38. This figure is derived as follows: in the original Kennedy et al., (2001) paper, the change from baseline score for placebo was -1.15 and for 960 mg was -41.53.10. The difference between these two change scores is 40.38. The original paper reported a significant difference between placebo and 960 mg at this time point (see figure 2 in Kennedy et al., (2001). Using the below tables, we can now see the magnitude of difference related to the significant change from baseline effects reported in the original papers. In the above example, this would equate to *d*_z_ =7.92.

Table 1. Kennedy et al., (2001): Placebo vs 320mg

|  | 1 hour | | | | 2.5 hours | | | | 4 hours | | | | 6 hours | | | |
| --- | --- | --- | --- | --- | --- | --- | --- | --- | --- | --- | --- | --- | --- | --- | --- | --- |
|  | Mean | *d_z_* | LCI | UCI | Mean | *d_z_* | LCI | UCI | Mean | *d_z_* | LCI | UCI | Mean | *d_z_* | LCI | UCI |
| Quality of Memory | 13.43 | **2.19** | *1.38* | *3.00* | 4.98 | **0.46** | *-0.19* | *1.11* | 0.90 | **0.09** | *-0.55* | *0.73* | 19.55 | **2.65** | *1.77* | *3.53* |
| Secondary Memory | 7.14 | **0.91** | *0.23* | *1.58* | 7.50 | **0.72** | *0.06* | *1.38* | 9.96 | **0.84** | *0.17* | *1.50* | 19.00 | **2.25** | *1.43* | *3.06* |
| Working Memory | 6.29 | **1.67** | *0.93* | *2.41* | 27.48 | **5.80** | *4.34* | *7.26* | -8.66 | **3.34** | *-4.33* | *-2.35* | 0.55 | **0.06** | *-0.58* | *0.70* |
| Speed of Memory | -16.82 | **0.09** | *-0.73* | *0.55* | -6.04 | **0.04** | *-0.68* | *0.60* | 94.74 | **0.71** | *0.05* | *1.37* | -19.91 | **0.09** | *-0.73* | *0.55* |
| Quality of Attention | 0.00 | **0.00** | *-0.64* | *0.64* | -0.20 | **0.45** | *-1.10* | *0.20* | 0.40 | **0.32** | *-0.32* | *0.97* | -0.65 | **0.31** | *-0.96* | *0.33* |
| Speed of Attention | 20.84 | **0.37** | *-0.28* | *1.01* | 27.85 | **0.52** | *-0.13* | *1.17* | 37.44 | **0.68** | *0.02* | *1.34* | 50.15 | **0.89** | *0.21* | *1.56* |

Table 2. Kennedy et al., (2001): Placebo vs 640mg

|  | 1 hour | | | | 2.5 hours | | | | 4 hours | | | | 6 hours | | | |
| --- | --- | --- | --- | --- | --- | --- | --- | --- | --- | --- | --- | --- | --- | --- | --- | --- |
|  | Mean | *d_z_* | LCI | UCI | Mean | *d_z_* | LCI | UCI | Mean | *d_z_* | LCI | UCI | Mean | *d_z_* | LCI | UCI |
| Quality of Memory | 3.86 | **0.49** | *-0.16* | *1.14* | 17.06 | **1.40** | *0.68* | *2.11* | -5.65 | **0.77** | *-1.43* | *-0.10* | 22.11 | **2.28** | *1.46* | *3.10* |
| Secondary Memory | -4.67 | **2.44** | *-3.28* | *-1.59* | 16.25 | **1.45** | *0.73* | *2.17* | -4.79 | **0.95** | *-1.62* | *-0.27* | 18.34 | **6.55** | *4.94* | *8.16* |
| Working Memory | 8.53 | **1.28** | *0.58* | *1.99* | 30.81 | **25.36** | *19.58* | *31.14* | -0.47 | **0.16** | *-0.80* | *0.48* | 3.78 | **1.20** | *0.50* | *1.90* |
| Speed of Memory | 30.98 | **0.22** | *-0.42* | *0.87* | 76.62 | **0.63** | *-0.02* | *1.29* | 28.89 | **0.19** | *-0.45* | *0.83* | 1.29 | **0.01** | *-0.63* | *0.65* |
| Quality of Attention | -0.05 | **0.05** | *-0.69* | *0.59* | -1.25 | **2.38** | *-3.21* | *-1.54* | -0.95 | **1.08** | *-1.76* | *-0.39* | -1.00 | **0.50** | *-1.15* | *0.15* |
| Speed of Attention | -7.30 | **0.10** | *-0.74* | *0.54* | -10.26 | **0.15** | *-0.79* | *0.49* | 28.46 | **0.40** | *-0.25* | *1.05* | -7.69 | **0.11** | *-0.75* | *0.53* |

Table 3. Kennedy et al., (2001): Placebo vs 960mg

|  | 1 hour | | | | 2.5 hours | | | | 4 hours | | | | 6 hours | | | |
| --- | --- | --- | --- | --- | --- | --- | --- | --- | --- | --- | --- | --- | --- | --- | --- | --- |
|  | Mean | *d_z_* | LCI | UCI | Mean | *d_z_* | LCI | UCI | Mean | *d_z_* | LCI | UCI | Mean | *d_z_* | LCI | UCI |
| Quality of Memory | 40.38 | **7.92** | *6.01* | *9.82* | 14.34 | **1.21** | *0.51* | *1.90* | 20.46 | **3.50** | *2.48* | *4.52* | 37.26 | **4.69** | *3.45* | *5.93* |
| Secondary Memory | 38.16 | **15.24** | *11.73* | *18.74* | 13.50 | **1.04** | *0.36* | *1.72* | 22.37 | **3.89** | *2.80* | *4.98* | 44.92 | **12.51** | *9.61* | *15.42* |
| Working Memory | 2.21 | **0.58** | *-0.07* | *1.23* | 30.84 | **39.78** | *30.75* | *48.81* | -1.52 | **1.28** | *-1.98* | *-0.58* | -7.65 | **0.69** | *-1.35* | *-0.03* |
| Speed of Memory | 42.91 | **0.26** | *-0.38* | *0.91* | 54.60 | **0.36** | *-0.29* | *1.01* | 48.45 | **0.26** | *-0.38* | *0.90* | 9.63 | **0.07** | *-0.57* | *0.71* |
| Quality of Attention | -0.50 | **0.47** | *-1.11* | *0.18* | -1.30 | **2.91** | *-3.82* | *-1.99* | -0.10 | **0.07** | *-0.71* | *0.57* | -0.45 | **0.25** | *-0.89* | *0.39* |
| Speed of Attention | -6.78 | **0.10** | *-0.74* | *0.54* | -10.44 | **0.15** | *-0.79* | *0.49* | 23.23 | **0.36** | *-0.29* | *1.00* | 2.21 | **0.03** | *-0.61* | *0.67* |

Table 4. Kennedy et al., (2002): Placebo vs Ginkgo

|  | 1 hour | | | | 2.5 hours | | | | 4 hours | | | | 6 hours | | | |
| --- | --- | --- | --- | --- | --- | --- | --- | --- | --- | --- | --- | --- | --- | --- | --- | --- |
| Measure | Mean | *d_z_* | LCI | UCI | Mean | *d_z_* | LCI | UCI | Mean | *d_z_* | LCI | UCI | Mean_ | *d_z_* | LCI | UCI |
| Quality of Memory | 18.58 | **0.97** | *0.29* | *1.65* | 21.20 | **1.35** | *0.64* | *2.06* | 13.90 | **1.39** | *0.68* | *2.10* | 29.37 | **1.48** | *0.75* | *2.20* |
| Secondary Memory | 23.33 | **1.81** | *1.05* | *2.57* | 19.56 | **1.18** | *0.49* | *1.87* | 15.33 | **1.57** | *0.84* | *2.30* | 27.67 | **1.98** | *1.19* | *2.76* |
| Working Memory | -4.76 | **1.02** | *-1.70* | *-0.34* | 1.64 | **0.23** | *-0.41* | *0.87* | -1.44 | **0.24** | *-0.89* | *0.40* | 1.70 | **0.17** | *-0.47* | *0.81* |
| Speed of Memory | 30.22 | **0.14** | *-0.50* | *0.78* | -12.77 | **0.07** | *-0.71* | *0.57* | 55.05 | **0.41** | *-0.24* | *1.06* | 52.08 | **0.32** | *-0.33* | *0.96* |
| Quality of Attention | 1.25 | **1.55** | *0.82* | *2.28* | 1.65 | **2.72** | *1.83* | *3.60* | -0.15 | **0.12** | *-0.76* | *0.52* | -0.65 | **20.55** | *-25.25* | *-15.86* |
| Speed of Attention | -14.17 | **0.18** | *-0.82* | *0.46* | 7.98 | **0.12** | *-0.52* | *0.76* | 19.64 | **0.40** | *-0.24* | *1.05* | -22.75 | **0.39** | *-1.04* | *0.26* |
| Alert | 5.64 | **0.93** | *0.26* | *1.60* | 5.69 | **2.27** | *1.45* | *3.10* | 7.15 | **2.58** | *1.71* | *3.44* | 8.36 | **11.20** | *8.59* | *13.82* |
| Content | 6.47 | **1.21** | *0.51* | *1.91* | 4.39 | **0.91** | *0.23* | *1.58* | 8.03 | **1.68** | *0.93* | *2.42* | 7.67 | **2.17** | *1.36* | *2.97* |
| Calm | -1.63 | **0.54** | *-1.19* | *0.11* | -2.42 | **3.10** | *-4.05* | *-2.15* | -0.38 | **0.13** | *-0.77* | *0.51* | -9.36 | **4.42** | *-5.60* | *-3.23* |
| Serial3 | -1.11 | **0.06** | *-0.70* | *0.58* | 0.17 | **0.01** | *-0.63* | *0.65* | 3.06 | **0.17** | *-0.47* | *0.81* | 4.06 | **0.30** | *-0.35* | *0.94* |
| Serial3Errors | 0.50 | **0.55** | *-0.10* | *1.20* | -0.71 | **3.85** | *-4.93* | *-2.77* | -2.66 | **26.60** | *-32.65* | *-20.55* | -2.22 | **2.94** | *-3.87* | *-2.02* |
| Serial7 | 2.37 | **0.18** | *-0.46* | *0.82* | 1.21 | **0.08** | *-0.56* | *0.72* | 3.37 | **0.23** | *-0.41* | *0.87* | 4.84 | **0.37** | *-0.27* | *1.02* |
| Serial7Errors | 0.63 | **0.82** | *0.16* | *1.49* | 0.36 | **0.47** | *-0.18* | *1.12* | -1.26 | **1.13** | *-1.82* | *-0.44* | 0.31 | **0.26** | *-0.38* | *0.90* |

Table 5. Kennedy et al., (2002): Placebo vs Ginseng

|  | 1 hour | | | | 2.5 hours | | | | 4 hours | | | | 6 hours | | | |
| --- | --- | --- | --- | --- | --- | --- | --- | --- | --- | --- | --- | --- | --- | --- | --- | --- |
|  | Mean | *d_z_* | LCI | UCI | Mean | *d_z_* | LCI | UCI | Mean | *d_z_* | LCI | UCI | Mean | *d_z_* | LCI | UCI |
| Quality of Memory | -2.64 | **0.12** | *-0.76* | *0.52* | 15.15 | **1.17** | *0.47* | *1.86* | 26.94 | **7.01** | *5.30* | *8.72* | 18.41 | **1.14** | *0.45* | *1.83* |
| Secondary Memory | 7.25 | **0.52** | *-0.13* | *1.17* | 17.96 | **1.36** | *0.65* | *2.07* | 32.58 | **3.96** | *2.86* | *5.06* | 25.42 | **1.94** | *1.16* | *2.72* |
| Working Memory | -9.90 | **2.28** | *-3.10* | *-1.46* | -2.81 | **0.41** | *-1.06* | *0.23* | -5.65 | **0.47** | *-1.11* | *0.18* | -7.01 | **0.41** | *-1.06* | *0.23* |
| Speed of Memory | -28.22 | **0.14** | *-0.78* | *0.51* | -80.53 | **0.42** | *-1.07* | *0.22* | -93.53 | **0.71** | *-1.37* | *-0.05* | -8.87 | **0.06** | *-0.70* | *0.58* |
| Quality of Attention | 1.30 | **1.95** | *1.17* | *2.73* | 2.55 | **4.56** | *3.34* | *5.77* | 1.60 | **3.53** | *2.51* | *4.56* | 0.90 | **3.53** | *2.51* | *4.55* |
| Speed of Attention | -11.04 | **0.15** | *-0.79* | *0.49* | -2.93 | **0.05** | *-0.69* | *0.59* | 17.37 | **0.37** | *-0.28* | *1.01* | -17.58 | **0.41** | *-1.06* | *0.24* |
| Alert | 2.19 | **0.33** | *-0.32* | *0.97* | 3.29 | **2.00** | *1.22* | *2.79* | 4.47 | **4.30** | *3.13* | *5.46* | 1.58 | **0.73** | *0.07* | *1.39* |
| Content | 2.14 | **0.41** | *-0.24* | *1.06* | 4.00 | **1.79** | *1.03* | *2.55* | 3.06 | **2.19** | *1.38* | *3.00* | 3.08 | **0.69** | *0.03* | *1.35* |
| Calm | -3.56 | **0.59** | *-1.24* | *0.07* | -2.42 | **0.95** | *-1.63* | *-0.28* | 2.58 | **0.67** | *0.01* | *1.33* | -1.98 | **0.41** | *-1.06* | *0.24* |
| Serial3 | 1.23 | **0.07** | *-0.57* | *0.71* | -0.36 | **0.02** | *-0.66* | *0.62* | 0.17 | **0.01** | *-0.63* | *0.65* | 4.28 | **0.34** | *-0.31* | *0.98* |
| Serial3Errors | -1.11 | **1.25** | *-1.95* | *-0.55* | -0.72 | **0.78** | *-1.45* | *-0.12* | -0.28 | **0.16** | *-0.80* | *0.49* | -0.45 | **0.19** | *-0.83* | *0.45* |
| Serial7 | 0.10 | **0.01** | *-0.63* | *0.65* | 0.90 | **0.07** | *-0.57* | *0.71* | 2.16 | **0.18** | *-0.46* | *0.82* | 2.42 | **0.20** | *-0.44* | *0.84* |
| Serial7Errors | 0.05 | **0.07** | *-0.57* | *0.71* | -0.22 | **0.42** | *-1.07* | *0.22* | -1.42 | **1.22** | *-1.92* | *-0.53* | -1.32 | **2.42** | *-3.26* | *-1.58* |

Table 6. Kennedy et al., (2002): Placebo vs Ginkgo/Ginseng

|  | 1 hour | | | | 2.5 hours | | | | 4 hours | | | | 6 hours | | | |
| --- | --- | --- | --- | --- | --- | --- | --- | --- | --- | --- | --- | --- | --- | --- | --- | --- |
|  | Mean | *d_z_* | LCI | UCI | Mean | *d_z_* | LCI | UCI | Mean | *d_z_* | LCI | UCI | Mean | *d_z_* | LCI | UCI |
| Quality of Memory | 37.17 | **1.95** | *1.17* | *2.72* | 44.03 | **2.24** | *1.42* | *3.05* | 29.32 | **1.65** | *0.91* | *2.39* | 5.65 | **0.30** | *-0.35* | *0.94* |
| Secondary Memory | 26.29 | **2.28** | *1.45* | *3.10* | 36.71 | **2.33** | *1.50* | *3.16* | 18.92 | **2.39** | *1.55* | *3.23* | -7.83 | **0.65** | *-1.31* | *0.00* |
| Working Memory | 10.87 | **1.67** | *0.92* | *2.41* | 7.32 | **1.10** | *0.41* | *1.79* | 10.39 | **1.37** | *0.66* | *2.08* | 13.48 | **2.50** | *1.65* | *3.36* |
| Speed of Memory | 8.91 | **0.04** | *-0.60* | *0.68* | 1.88 | **0.01** | *-0.63* | *0.65* | -33.83 | **0.21** | *-0.85* | *0.43* | 26.08 | **0.14** | *-0.50* | *0.78* |
| Quality of Attention | 0.90 | **2.58** | *1.71* | *3.44* | 1.15 | **1.89** | *1.12* | *2.66* | 0.10 | **0.18** | *-0.46* | *0.82* | 0.50 | **1.57** | *0.84* | *2.31* |
| Speed of Attention | 5.97 | **0.00** | *-0.64* | *0.64* | 5.67 | **0.00** | *-0.64* | *0.64* | 29.66 | **0.01** | *-0.63* | *0.65* | -13.65 | **0.00** | *-0.64* | *0.64* |
| Alert | -2.31 | **0.33** | *-0.97* | *0.32* | -2.50 | **0.38** | *-1.03* | *0.26* | 1.13 | **0.24** | *-0.40* | *0.88* | 0.12 | **0.03** | *-0.61* | *0.67* |
| Content | 3.09 | **0.54** | *-0.12* | *1.19* | 7.16 | **1.26** | *0.56* | *1.96* | 5.95 | **2.07** | *1.27* | *2.86* | 7.90 | **1.63** | *0.90* | *2.37* |
| Calm | -1.20 | **0.44** | *-1.08* | *0.21* | 2.11 | **0.97** | *0.29* | *1.64* | 0.80 | **0.26** | *-0.38* | *0.91* | -2.83 | **0.76** | *-1.42* | *-0.09* |
| Serial3 | -0.11 | **0.01** | *-0.65* | *0.63* | -0.64 | **0.03** | *-0.67* | *0.61* | 3.06 | **0.18** | *-0.46* | *0.82* | 6.17 | **0.50** | *-0.15* | *1.15* |
| Serial3Errors | -0.16 | **0.13** | *-0.77* | *0.51* | -0.56 | **4.17** | *-5.32* | *-3.03* | -0.05 | **0.02** | *-0.66* | *0.62* | -1.28 | **0.81** | *-1.47* | *-0.14* |
| Serial7 | -1.27 | **0.09** | *-0.74* | *0.55* | 1.06 | **0.08** | *-0.56* | *0.72* | 3.48 | **0.27** | *-0.37* | *0.92* | 0.52 | **0.04** | *-0.60* | *0.68* |
| Serial7Errors | 0.16 | **0.24** | *-0.40* | *0.88* | 0.57 | **1.34** | *0.63* | *2.05* | -0.52 | **0.47** | *-1.12* | *0.18* | -1.79 | **1.73** | *-2.48* | *-0.98* |
